# Supplementary material for: Interpretable network propagation with application to expanding the repertoire of human proteins that interact with SARS-CoV-2
Source: Gigascience. 2021 Dec 29;10(12):giab082. doi: 10.1093/gigascience/giab082 (PMC8716363; doi:10.1093/gigascience/giab082)
Supplement: giab082_GIGA-D-21-00100_Revision_1 [file giab082_giga-d-21-00100_revision_1.pdf]

GigaScience

Interpretable Network Propagation with Application to Expanding the Repertoire of Human Proteins that Interact with SARS-CoV-2

--Manuscript Draft--

Manuscript Number:	GIGA-D-21-00100R1
Full Title:	Interpretable Network Propagation with Application to Expanding the Repertoire of Human Proteins that Interact with SARS-CoV-2
Article Type:	Research

Funding Information:

Funder	Recipient
Division of Biological Infrastructure (DBI-17598)	Prof. T. M. Murali
Division of Molecular and Cellular Biosciences (MCB-1817736)	Prof. T. M. Murali
Division of Computer and Network Systems (CNS-1618207)	Prof. Mark Crovella
Division of Computing and Communication Foundations (CCF-2029543)	Prof. Judith Klein-Seetharaman
Division of Chemical, Bioengineering, Environmental, and Transport Systems (CBET-1510920)	Prof. Padmavathy Rajagopalan
National Institute of Food and Agriculture (2018-07578)	Prof. Padmavathy Rajagopalan

Abstract:

Background: Network propagation has been widely used for nearly 20 years to predict gene functions and phenotypes. Despite the popularity of this approach, little attention has been paid to the question of provenance tracing in this context, e.g., determining how much any experimental observation in the input contributes to the score of every prediction.

Results: We design a network propagation framework with two novel components and apply it to predict human proteins that directly or indirectly interact with SARS-CoV-2 proteins. First, we trace the provenance of each prediction to its experimentally validated sources, which in our case are human proteins experimentally determined to interact with viral proteins. Second, we design a technique that helps to reduce the manual adjustment of parameters by users. We find that for every top-ranking prediction, the highest contribution to its score arises from a direct neighbor in a human protein-protein interaction network. We further analyze these results to develop functional insights on SARS-CoV-2 that expand on known biology such as the connection between endoplasmic reticulum stress, HSPA5, and anti-clotting agents.

Conclusions: We examine how our provenance tracing method can be generalized to a broad class of network-based algorithms. We provide a useful resource for the SARS-CoV-2 community that implicates many previously undocumented proteins with putative functional relationships to viral infection. This resource includes potential drugs that can be opportunistically repositioned to target these proteins. We also discuss how our overall framework can be extended to other, newly-emerging viruses.

Corresponding Author:	T. M. Murali<br>Virginia Tech: Virginia Polytechnic Institute and State University<br>Blacksburg, VA UNITED STATES
Corresponding Author Secondary Information:	
Corresponding Author's Institution:	Virginia Tech: Virginia Polytechnic Institute and State University
Corresponding Author's Secondary Institution:	
First Author:	Jeffrey N. Law
First Author Secondary Information:	

Powered by Editorial Manager® and ProduXion Manager® from Aries Systems Corporation

**Order of Authors:**	Jeffrey N. Law
	Kyle Akers
	Nure Tasnina
	Catherine M. Della-Santina
	Shay Deutsch
	Meghana Kshirsagar
	Judith Klein-Seetharaman
	Mark Crovella
	Padmavathy Rajagopalan
	Simon Kasif
	T. M. Murali
**Order of Authors Secondary Information:**	
**Response to Reviewers:**	Response to Reviewers<br>Interpretable Network Propagation with Application to Expanding the Repertoire of Human Proteins that Interact with SARS-CoV-2<br><br>We thank the reviewers for their insightful comments and suggestions. We made a few changes to the manuscript and added several analyses to the supplement. We highlight these changes in the manuscript using a different color.<br><br>The changes in the main manuscript include restructuring “Analysis” to give more importance to our main contributions on provenance tracing:<br>1. We shortened the subsection “Prioritization of Potential SARS-CoV-2 Interactors” by moving the cross validation results to the supplementary results. Consequently, this subsection contains the evaluations of predictions made by RL in terms of overlap with other datasets and functional enrichment. We retained these analyses in the main text since they confirm that our predictions are biologically meaningful and relevant to the virus.<br>2. We reordered the section “Tracing the Provenance of Top-Ranking Proteins” by moving the analysis of alpha below the main results for our provenance tracing approach.<br><br>The analyses we added are as follows:<br>1. Results for Random Walk with Restarts (RWR) in the sections on cross-validation (CV), parameter selection, and overlap among algorithms.<br>2. Comparison to PPI networks (BioGRID, BioGRID-Y2H, HI-union) for the effective diffusion results.<br>3. Overlap of our top-ranking proteins with two more experimentally determined sets<br>a. Differential protein abundance in SARS-CoV-2–infected iAT2 cells.<br>b. Differential gene expression in upper airway samples in SARS– CoV-2–infected patients<br><br>Finally, we divided the Supplementary Information into three parts: Supplementary Methods, Supplementary Results, and Supplementary Figures.<br><br>We address each reviewer’s comments in detail below.<br><br>Reviewer 1<br>The article provides information, useful and verifiable; presenting an adequate methodological approach applied to the study of the most important public health problem that now urges us. However, unlike what the authors argue in some sections, I do not consider it very original, as it is only a refinement of what has been done in propagation studies in networks for some time, but without a doubt its results are valuable, which more than justifies its publication. On the other hand, the mathematical description of the algorithm is quite clear, much clearer than what is described in the text of the document, which I suggest to review, as it is difficult in some sections.

Powered by Editorial Manager® and ProduXion Manager® from Aries Systems Corporation

We appreciate the reviewer's viewpoint. We hope that the reviewer finds that our restructuring of the text and edits have improved the clarity of the presentation.

Reviewer 2
The authors designed a network propagation framework with two novel components and applied it to predict human proteins that directly or indirectly interact with SARS-CoV-2 proteins. There are several major problems:
1. There have been several similar studies which identified covid-19 infection-related human genes based on a random walk model in a virus-human protein interaction network (PMID: 32685484). The authors need to compare with previous works.

Thank you for this suggestion. Since the cited manuscript uses RWR as the primary algorithmic component, we also executed RWR using the same source nodes (i.e., human proteins that interact with SARS-CoV-2 proteins), and found that it produced similar results to RL. We added a citation to this specific paper suggested by the reviewer in a paragraph on related work in "Discussion".

2. The authors used network-based method to predict SARS-CoV-2 interaction protein. But there is plenty of gene expression data. The authors should compare the network genes with expression genes, such as PMID: 33505977 and 33519902.

We appreciate the recommendation to compare network-based predictions with differential gene expression data. We have added a paragraph to "Analyses" discussing the overlap between our top-ranking predictions and experimental data sets identifying differential protein abundance and gene expression in response to SARS-CoV-2 infection. We have updated Figure 2(a) with the results for protein abundance and added Supplementary Figure S8 for the overlap with differential gene expression data. We have also cited the relevant papers that published the original experimental data.

3. Within the network methods, there are network embedding (PMID: 31455874), network diffusion (PMID: 30069494), random walk with restart (PMID: 28787010, 28932904), shortest path (PMID: 28076954, 27434024, 26209080, 28974058, 23762832, 24056857, 22496748) and guilt-by-association (doi: 10.1016/j.neucom.2015.09.136). These methods should be introduced.

Thank you for this idea. Please note that the first seven papers that we have already cited in our manuscript are among the earliest in the field of network biology (published between 2003 and 2008) that use ideas in network diffusion, random walks, and guilt by association. We also cite an early review in the field that covers these topics, including shortest paths. In addition, the first paper we cite in "Analyses" is a more recent review that covers the topics mentioned by the reviewer. Our perspective is that it is important to cite such papers that may have influenced the field.

In this work, we compared a specific, representative set of network methods, including network embedding (deepNF), network diffusion (RL, GeneMANIA, and RWR; note that RWR is in fact a network diffusion method), and guilt-by-association (Local). We chose not to compare with shortest path approaches since network propagation methods implicitly consider all paths in the network, with parameters (e.g., the restart parameter for RWR) controlling for the relative importance of short vs long paths.

4. The supplementary materials are different from the descriptions of File S1 … Figure S4. Please check them carefully.

Thank you for pointing out this error. We have checked and corrected the supplementary files. Please note that there are only three files now.

Reviewer 3
In their manuscript, Law and colleagues use network propagation to predict potential SARS-CoV-2-interacting human proteins. On the methods front, they propose a means

Powered by Editorial Manager® and ProduXion Manager® from Aries Systems Corporation

to (1) track down the prioritization contributions of individual seed proteins experimentally documented to interact with the virus; (2) select the main parameter of their network propagation method of choice (regularized Laplacian - RL) in an unbiased manner by relating it to the expected shortest path length of the random walk.

Overall, the individual sections of the paper are clear and well-written. However, taken together, the two main analyses somewhat lack a unifying narrative and feel disconnected from each other. While the methodological contributions of the paper seem interesting, the core choice of methods in the application to SARS-CoV-2 seems inadequately justified. Below are my two major concerns pertaining to each part of the analysis.

We very much appreciate the reviewer's careful inspection of our work and considerable effort put into their review. We hope that the substantial restructuring of the manuscript and additional analyses and rationale we have provided address the concerns raised by the reviewer.

Major comments
1) In general, in the first (prioritization) part of the analysis, it is not clear to me what the actual goal is. RL is benchmarked against several types of network propagation-based and "traditional" prioritization approaches, then SVM is taken as a representative of the latter group. Proteins are then prioritized between these two methods and validated in silico by their overlap with literature and their functional annotations. As it stands, the point of discussing and comparing different methods is not clear. What makes RL stand out is not directly evident the way these results are presented. In fact, the entire first part of the results, in my view, does not make a sufficient case for the use of network propagation as the prioritization method of choice. It rather looks like other off-the-shelf methods perform comparably (Fig. 2a), and yield similar biological insights (Fig 2c). It seems to me that if comparison of methods was performed, the authors should at least focus on the unique insights provided by RL (e.g. in terms of GO terms), if any, compared to SVM.

We acknowledge the reviewer's point. Our main goal was to apply the RL and the provenance tracing method we developed to predict potential interactors of SARS-CoV-2. Rather than use RL directly, we first compared it to many different approaches to solve the same problem. Based on the reviewer's comments, we have statistically compared the distributions of AUROC, AUPRC, and precision at 0.3 recall of all pairs of algorithms in "Comparison of Cross-Validation Results" in the Supplementary Results. RWR and deepNF had slightly higher AUPRC and precision at 0.3 recall than RL for the 1:5 and 1:10 positive:negative ratios but not for the 1:1 ratio. Nevertheless, we selected RL (and SVM) for subsequent analyses as we explain in the main manuscript:

"To decide which methods to select for subsequent analyses,we compared them using 5-fold cross validation ("Comparison of Cross-Validation Results" in the supplementary text and Figure S1). RL, random walk with restarts (RWR) [23], and deepNF [26] had the highest values of area under the precision-recall curve followed by SVM and logistic regression. RL achieved marginally worse values of area under the precision-recall curve than RWR and deepNF. We selected one network propagation method (RL) and one supervised classifier (SVM) for the following reasons. We preferred RL over deepNF because the provenance tracing method we developed for RL enabled its results to be more easily interpreted than those for deepNF. Since RL and RWR produced highly similar predictions with a very high Spearman's correlation for the ranking of all proteins ("Overlap among algorithms" in the supplementary text and Figure S2), we selected RL as representative of the two methods. We chose SVM among the two off-the-shelf classifiers since it also had very good performance in cross-validation."

This reasoning also addresses the question that the reviewer raises about the choice of RL in the next comment. We acknowledge that using RWR instead of RL may have yielded similar results for provenance tracing. We intend to perform this analysis in a subsequent project.

Powered by Editorial Manager® and ProduXion Manager® from Aries Systems Corporation

2) Provenance tracing explored in the second part is very interesting as a premise, and the fact that it is relatively uninvestigated makes it an appealing topic. As the authors note, it is based on a simple principle: once the regularized Laplacian kernel is calculated, the values of the kernel matrix are row-sorted for each protein. While this is still, in a sense, a steady-state view, and the name "provenance tracing" inspires in the reader's mind a sequence of connections between seeds and targets, I think the authors did a good job showcasing how it simplifies the subnetworks related to each biological process of interest, improving interpretability. I am convinced by its utility in that sense. What I have been having trouble convincing myself about is the particular choice of RL, some claims around the novelty of the analytical results, and why \alpha matters in the first place. To elaborate:

2a) The rationale presented for putting emphasis on \alpha seems not so well-justified to me. The authors state that they looked for a different way to determine alpha than looking at AUROC/AUPRC values since they varied little over a wide range of alpha. Does this not mean that the choice of alpha does not impact the prediction results in the case of RL? I guess I don't fully understand the point of the derivation other than a purely mathematical exercise (whose precedents seem to exist - see below). This also bears asking why RL was chosen in the first place. It sounds in the paper as if RL is the only choice of kernel that can be utilized this way to compute the contribution of the seed nodes, whereas many other types of kernels should work in a similar manner (see Fouss et al. cited by the authors as [18]). It seems that for provenance tracing, any type of kernel could work as the authors simply use the values corresponding to the seeds at the steady state kernel matrix to represent the contribution of each seed on the candidate protein's score. For example, RL is indeed similar to RWR, a widely used propagation method in biology (see Köhler, Sebastian, et al. "Walking the interactome for prioritization of candidate disease genes." The American Journal of Human Genetics 82.4 (2008): 949-958), in terms of the form of its kernel. deepNF, one of the methods considered in the benchmark that does comparably or better than RL, is also based on RWR. Could the authors comment on the above points?

We agree with the reviewer that the choice of alpha does not impact the predictions in the case of RL. It was important for us to have tested different values of alpha before reaching this conclusion. Although varying alpha did not have a major effect on the predictions for RL in this particular application, it may have a larger impact for other prediction problems.

We also agree that the RL is not the only kernel to which this type of provenance tracing is applicable. We mention this point at two places in the manuscript:
(i) Abstract: “We examine how our provenance tracing method can be generalized to a broad class of network-based algorithms.”
(ii) Discussion: “Our strategy for tracing provenance extends to any algorithm that makes predictions using a linear combination of evidence such as logistic regression and GeneMania [61]. In particular, it is applicable to the large number of random-walk-based methods that have been developed for predicting disease genes or annotations to GO terms [62, 63, 64, 65].”

Although deepNF is based on RWR, it uses an autoencoder to learn a low-dimensional embedding of the input network and an SVM with an RBF kernel trained on this embedding to solve the node labeling problem. Developing a provenance tracing strategy for deepNF is non-trivial and will require a substantial research effort.

2b) The authors take a mean-field approach relating the teleportation/damping parameter (\alpha) to the expected value of path lengths given a network. The authors then use the actual network to determine the median path length between seeds and candidates and find the \alpha that corresponds to this value from their precomputed lookup table. In a way, we can see this parameter selection process as the tuning of the random walk according to the network at hand. Regarding the analytical results on average path lengths in random walks/diffusion processes, similar results seem to exist:
Yazdani, Majid, and Andrei Popescu-Belis. "A random walk framework to compute textual semantic similarity: a unified model for three benchmark tasks." 2010 IEEE Fourth International Conference on Semantic Computing. IEEE, 2010.
Ghosh, Rumi, et al. "Non-conservative diffusion and its application to social network

Powered by Editorial Manager® and ProduXion Manager® from Aries Systems Corporation

analysis." arXiv preprint arXiv:1102.4639 (2011).
Ghosh, Rumi, and Kristina Lerman. "Rethinking centrality: the role of dynamical processes in social network analysis." arXiv preprint arXiv:1209.4616 (2012).
Stojmirović, Aleksandar, and Yi-Kuo Yu. "Information flow in interaction networks." Journal of Computational Biology 14.8 (2007): 1115-1143.
Masuda, Naoki, Mason A. Porter, and Renaud Lambiotte. "Random walks and diffusion on networks." Physics reports 716 (2017): 1-58.
Could the authors elaborate on the difference of their approach and scope?

We appreciate the referee's attention to this aspect of our work, which encourages us to expand on the motivation and novelty of our analysis.

The use of diffusion and random walks (broadly, "stochastic processes") as tools in network analysis is extensive. However, a dimension that is not always emphasized in such analyses is the distinction between processes that evolve in discrete time (eg, "random walks" that evolve in "steps" or "iterations") versus those that evolve in continuous time (eg, "diffusion" that evolves via "fluid flow"). These two kinds of processes require different analytic tools. Discrete-time processes (as exemplified by PageRank and random-walk-with-restarts) are more common, and their analysis is often simpler. However, the Regularized Laplacian (RL) that we rely on corresponds to the solution of a continuous-time process (eg, it can be described in terms of a fluid flow). The tools for analyzing such processes are based on continuous-time Markov chains (CTMCs), ie, systems of differential equations.

As noted by the referee, our work tunes the mean path length of a time-bounded continuous time random walk to match average path lengths in biological processes. To do so, the analytic challenge we overcome comes from the fact that path lengths take on discrete values, while the random walk evolves in continuous time, and the time bound is a random variable.

We note that even specialized references do not contain discussion of expected path lengths in the CTMC corresponding to a Regularized Laplacian. For example, the question is not discussed in the recent, comprehensive text "Algorithms and Models for Network and Link Analysis," (Fouss et al). Before writing up our analysis, we searched the literature and contacted experts in the field of stochastic processes to discern if this problem had previously been solved. We found no evidence that it had been addressed before.

With respect to the papers pointed to by the referee, each does indeed contain consideration of path lengths, but most are concerned only with models based on discrete time. None of the papers considers our question specifically. Thus the results of those papers cannot be used to answer the question we address.

Specifically:
Yazdani, Majid, and Andrei Popescu-Belis. "A random walk framework to compute textual semantic similarity: a unified model for three benchmark tasks." 2010 IEEE Fourth International Conference on Semantic Computing. IEEE, 2010.

This paper uses "hitting time" and "commute time" as measures of walk length. Although these terms involve the word "time", here time is understood to be a discrete quantity. For example, hitting time (i,j) is the mean number of steps in a discrete time random walk starting at i, up to the first visit to node j.

Ghosh, Rumi, et al. "Non-conservative diffusion and its application to social network analysis." arXiv preprint arXiv:1102.4639 (2011).

This paper studies "non-conservative" diffusion, which can also be described as epidemic modeling. That model is fundamentally different from a random walk. This paper too is only concerned with discrete time processes.

Ghosh, Rumi, and Kristina Lerman. "Rethinking centrality: the role of dynamical processes in social network analysis." arXiv preprint arXiv:1209.4616 (2012).

Powered by Editorial Manager® and ProduXion Manager® from Aries Systems Corporation

This paper, a precursor to the Ghosh et al paper above, is also concerned with discrete time random walks. Furthermore, the path lengths that it analyzes are paths in a non-conservative process, that is, paths of infection in an epidemic.

Stojmirović, Aleksandar, and Yi-Kuo Yu. "Information flow in interaction networks." Journal of Computational Biology 14.8 (2007): 1115-1143.

This paper includes discussion of a diffusion process and average path lengths. However the diffusion process is specific to this paper (not a commonly-used process like the RL) and path lengths are observed empirically (ie, no mathematical expression for path length is derived).

Masuda, Naoki, Mason A. Porter, and Renaud Lambiotte. "Random walks and diffusion on networks." Physics reports 716 (2017): 1-58.

This paper, in contrast to those above, extensively covers both discrete and continuous time stochastic processes. However, the specific question we ask, namely, the mean path length in the RL model, is not addressed in this paper. This underscores that our problem is not just one of computing the probability of reaching a given node in a given amount of time (which this paper does address) but specifically of how many nodes the walker will pass through in a random amount of continuous time.

Minor comments
Aside from the above major concerns, below are my minor points and comments to help the authors improve their work:

3) In the Discussion: "We were surprised to see that the top-contributing sources were invariably direct neighbours of the top-ranking predictions in the STRING network. A partial explanation for this trend may be the fact that as many as 5,331 proteins in the STRING network were direct neighbors of at least one source protein, even when we considered only interactions with weight at least 0.9 (the STRING database deems edges with such weights to be of "very high quality"). Thus, the structure of the STRING network and central location of sources within it may cause the RL both to give high ranks only to direct neighbors of sources and to channel propagation primarily along these direct connections."
To recap, this part of the discussion is related to the fact that, even though global exploration by using teleports is favored over local neighbor-hopping by setting \alpha to a high value, the top contributions still come from directly connected seeds. To me, this is one of the most interesting findings in the paper that might also call into question the widespread use of diffusion-based prioritization on PPI networks if it really is the case that, no matter how globally explored the network is, it is still the seeds directly neighboring the prioritized proteins that affect the prioritization the most. One question related to that is whether or not the same holds for proteins that were lowly ranked by RL, i.e. is RL (and potentially other random-walk based methods) capturing chiefly "local" contributions from seeds for proteins at the bottom of the list as well? I feel that this can be explored further by (1) trying PPI networks with different densities and degree distributions than STRING such as strictly experimental binary PPIs such as those derived from Y2H assays (e.g. Luck, Katja, et al. "A reference map of the human binary protein interactome." Nature 580.7803 (2020): 402-408.); (2) testing whether the number of seeds itself (in this case around 300 if I followed the methods correctly) is a contributing factor to this phenomenon where, as the authors noted in the discussion, the direct interactions of these seed proteins cover the majority of the network, "saturating" the random walk process in a sense. Would we see the same results if there were, say, only 30 seed proteins instead of 300? In general, I would request the authors to think a little bit to delve a little further into this, without disrupting the flow of the paper as it is now.

Thank you for these suggestions. We found the first idea to be appealing. We repeated our experiments with three PPI networks: BioGRID, BioGRID with Y2H assays only, and the high-quality HI-union network from the paper the reviewer suggested. We originally hypothesized that the effective diffusion would be higher for the top-ranking proteins of these networks, but surprisingly, the influence from direct neighbors was

Powered by Editorial Manager® and ProduXion Manager® from Aries Systems Corporation

even greater for these networks, resulting in lower effective diffusion values (see Figures 3(f), S9, and Table 1). We discuss these results in the Analysis section.

We believe these points merit further investigation and plan to continue this research in a future paper.

4) Figure 1 seems too generic. Perhaps include some more details such as what the nodes are (red, virus, blue human proteins, etc.), node sizes, etc.

We have added a legend to the figure on the node colors and gradients.

5) Figure 2a - I could not find anywhere if the AUROC/AUPRC values are statistically significantly different between different methods. The median values are compared but it seems by looking at the error bars that the difference is perhaps not statistically significant with respect to the other methods.

We tested for statistical significance between the algorithms and added a discussion in the supplementary results section in “Comparison of Cross-Validation Results.”

6) Figure 2a caption: precision at 0.1 recall or 0.3 recall (latter one used in the text, former used in the caption)

We corrected the caption to “precision at 0.3 recall”.

7) Figure 2C - top-ranked, meaning top 332 or top 1000?

Figure 2C (now B) shows the top 332. We updated the text to clarify this difference.

8) The provenance tracing part of the analysis: an intuitive definition of \alpha would be helpful at the beginning of this section. Currently, it is introduced without such an explanation as to what it does, such as the damping or "teleportation" parameter equivalent in pagerank.

At the beginning of the section “Tracing the Provenance of Top-Ranking Proteins,” we attempt to give an intuitive idea of the effect of \alpha: “Hence for larger values of \alpha, the influence of the sources is diffused more broadly across the network.”

9) The flow of this results section should be revisited. The authors lead with a discussion on the sensitivity analysis of \alpha, which, in my opinion, is of secondary importance to the provenance tracing aspect. The most important part of the paper thus gets buried further down into the results section.

We agree with the reviewer’s suggestion. We have reorganized the results section so that provenance tracing comes earlier.

10) Figure 3A, figure order (comes after Figs 3B-E) in the text

We updated the figure order.

11) "The GO biological process "protein folding in endoplasmic reticulum" was also enriched in the top-ranking proteins (p-value 4.32 10-9 for RL and 0.28 for interactors of SARS-CoV-2)."
This sentence reads as if the ER related GO terms were identified through two independent processes, where in reality it was the GO term enrichment on the top-prioritized proteins that was done first and "protein folding on ER" was identified as a process of interest, and then provenance tracing was performed on this biological process because it was implicated by the enrichment analysis in the first place. Minor point but one pertains to the flow of the text nevertheless: I think it would be helpful to remind the reader the order of events that led to these results, i.e. first the identification of salient pathways of GO terms, and then a detailed x-ray of these pathways through provenance tracing.

We added the following sentence to the paragraph just before the section “The Role of Endoplasmic Reticulum Stress, HSPA5, and Anti-Clotting Drugs”:

	“We remind the reader that we computed functions enriched in the top-ranking proteins, performed the provenance analysis independently, and then integrated the results in the protein networks we visualized.”<br><br>12) Typo: "we compute the precise contribution of each source's contribution to the score of u."<br><br>We have corrected it.
**Additional Information:**	
**Question**	**Response**
Are you submitting this manuscript to a special series or article collection?	No
**Experimental design and statistics**<br><br>Full details of the experimental design and statistical methods used should be given in the Methods section, as detailed in our Minimum Standards Reporting Checklist. Information essential to interpreting the data presented should be made available in the figure legends.<br><br>Have you included all the information requested in your manuscript?	Yes
**Resources**<br><br>A description of all resources used, including antibodies, cell lines, animals and software tools, with enough information to allow them to be uniquely identified, should be included in the Methods section. Authors are strongly encouraged to cite Research Resource Identifiers (RRIDs) for antibodies, model organisms and tools, where possible.<br><br>Have you included the information requested as detailed in our Minimum Standards Reporting Checklist?	Yes
**Availability of data and materials**<br><br>All datasets and code on which the conclusions of the paper rely must be	Yes

either included in your submission or deposited in publicly available repositories (where available and ethically appropriate), referencing such data using a unique identifier in the references and in the "Availability of Data and Materials" section of your manuscript.<br><br>Have you have met the above requirement as detailed in our Minimum Standards Reporting Checklist?	

Powered by Editorial Manager® and ProduXion Manager® from Aries Systems Corporation

LaTeX - Main Document (TeX file)

Click here to access/download;LaTeX - Main Document (TeX file);sars-cov-2-networks.tex

Placeholder for OUP logo oup.pdf

Placeholder for journal logo gigascience-logo.pdf

GigaScience, 20xx, 1–12

doi: xx.xxxx/xxxx
Manuscript in Preparation

Interpretable Network Propagation with Application to Expanding the Repertoire of Human Proteins that Interact with SARS-CoV-2

Jeffrey N. Law[1,†], Kyle Akers[1], Nure Tasnina[2], Catherine M. Della Santina[3], Shay Deutsch[4], Meghana Kshirsagar[5], Judith Klein-Seetharaman[6], Mark Crovella[7], Padmavathy Rajagopalan[8], Simon Kasif[3] and T. M. Murali[2,*]

[1]Interdisciplinary Ph.D. Program in Genetics, Bioinformatics, and Computational Biology, Blacksburg, VA, USA and [2]Department of Computer Science, Virginia Tech, Blacksburg, VA, USA and [3]Department of Biomedical Engineering, Boston University, Boston, MA, USA and [4]Department of Mathematics, University of California, Los Angeles, CA, USA and [5]AI for Good Lab, Microsoft, Redmond, WA, USA and [6]Department of Chemistry, Colorado School of Mines, Golden, CO USA and [7]Department of Computer Science, Boston University, Boston, MA, USA and [8]Department of Chemical Engineering, Virginia Tech, Blacksburg, VA, USA

[*] Corresponding author: murali@cs.vt.edu [†] Current Address: National Renewable Energy Laboratory, Golden, CO, USA

Abstract

Background: Network propagation has been widely used for nearly 20 years to predict gene functions and phenotypes. Despite the popularity of this approach, little attention has been paid to the question of provenance tracing in this context, e.g., determining how much any experimental observation in the input contributes to the score of every prediction. **Results:** We design a network propagation framework with two novel components and apply it to predict human proteins that directly or indirectly interact with SARS-CoV-2 proteins. First, we trace the provenance of each prediction to its experimentally validated sources, which in our case are human proteins experimentally determined to interact with viral proteins. Second, we design a technique that helps to reduce the manual adjustment of parameters by users. We find that for every top-ranking prediction, the highest contribution to its score arises from a direct neighbor in a human protein-protein interaction network. We further analyze these results to develop functional insights on SARS-CoV-2 that expand on known biology such as the connection between endoplasmic reticulum stress, HSPA5, and anti-clotting agents. **Conclusions:** We examine how our provenance tracing method can be generalized to a broad class of network-based algorithms. We provide a useful resource for the SARS-CoV-2 community that implicates many previously undocumented proteins with putative functional relationships to viral infection. This resource includes potential drugs that can be opportunistically repositioned to target these proteins. We also discuss how our overall framework can be extended to other, newly-emerging viruses.

Key words: network propagation; computational prediction; interpretable machine learning; provenance tracing; SARS-CoV-2; COVID-19; virus-host protein interaction networks;

Compiled on: September 21, 2021.
Draft manuscript prepared by the author.

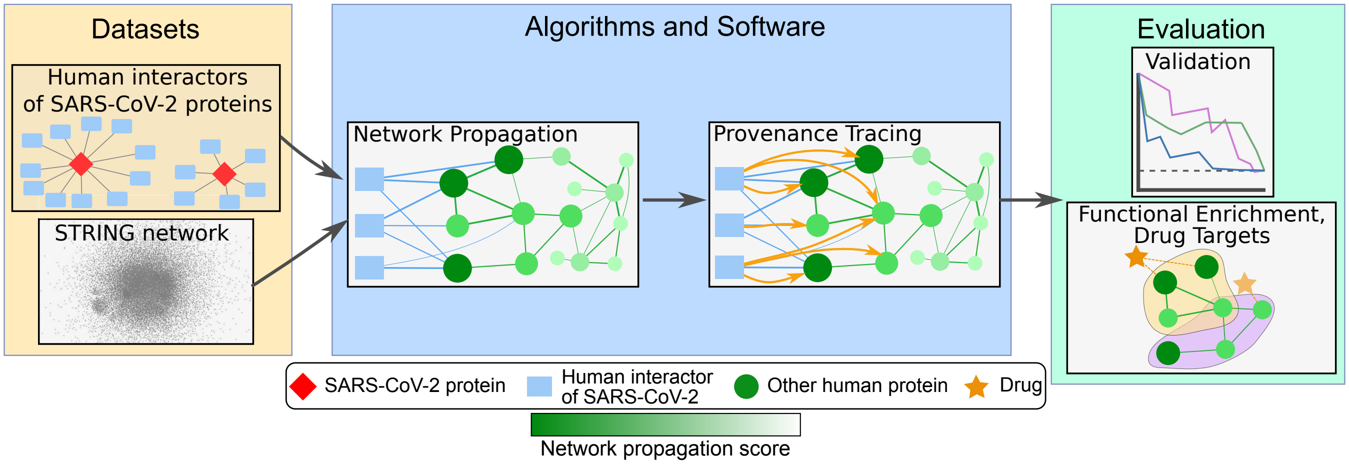

Figure 1. Overview of methodology. Algorithms and software for network propagation and provenance analysis take as input experimentally determined host-pathogen protein interactions and a human protein interaction network. Evaluation includes cross-validation, functional enrichment, and literature-based examination of promising protein targets and drugs.

Background

Network propagation algorithms have been widely used for nearly 20 years for function and phenotype prediction in systems biology [1, 2, 3, 4, 5, 6, 7]. More recently, applications of these techniques have included determination of genes associated with cancers and complex diseases [8] and denoising single-cell gene expression data [9]. Nowadays, network-based algorithms facilitate large-scale and automated data analysis of such complexity that it can be difficult for humans to understand the rationale that underlies a prediction, leading to decreased transparency and interpretability.

In this work, we consider the fundamental problem of tracing the provenance of a prediction back to the experimental sources [10]. Given a protein interaction network and a set of "sources", e.g., the human proteins that physically interact with SARS-CoV-2 [11], suppose we apply a network-based algorithm to score and prioritize additional proteins that may directly or indirectly interact with the virus. Can we determine which source proteins make the highest contribution to the score computed for each prediction? Surprisingly, this question has been insufficiently studied in the field of network biology [10]. This aspect takes particular importance in the context of COVID-19 or other clinically or scientifically critical applications, where it may be important to understand the rationale behind the computational prediction of a new drug target before committing to expensive experimental validation.

We present a simple and direct method to solve this problem for a large class of network propagation algorithms. Specifically, for each protein u in the network, we compute the precise contribution of each source to the score of u. This calculation enables us to sort the sources by their relative contributions to u and to quantify the relative roles of sources at different distances from u.

To evaluate the effectiveness of this strategy, we apply it to prioritize host proteins that may "functionally" (directly or indirectly) interact with SARS-CoV-2 proteins and host cellular processes that may be hijacked by the virus (Figure 1). To this end, we take advantage of a recently published dataset of human proteins that physically interact with SARS-CoV-2 [11]. Although these SARS-CoV-2 interactors are entry points to host cellular processes that may be hijacked by viral infection, the proteomics pipeline used to discover them [11] may not capture *in vivo* conditions and tissue-specific interactions, leading to false negatives. Therefore, we apply network propagation algorithms to these known human protein interactors of SARS-CoV-2 proteins (sources) and a whole-genome human protein interaction network from the STRING database [12]. We identify statistically-enriched host biological processes and pathways that include highly-ranking proteins computed by our methods. We illustrate how our provenance analysis can simplify visualizations of these processes and assist in understanding how they may be impacted by SARS-CoV-2.

Data Description

Here, we detail the different viral-human and human protein and functional interaction networks that we used in our study.

SARS-CoV-2–Human Protein-Protein Interactions (PPIs). We obtained 332 human proteins that interact with SARS-CoV-2 [11] and treated them as positive examples for our analysis. We added the ACE2 receptor to this set.

Functional and protein interaction networks. We used the human functional interaction network in the STRING database (version 11) [12], comprising of 18,886 nodes and 977,789 edges after applying a "medium" score cutoff of 400 and mapping to UniProt IDs. We used the interaction reliabilities provided by STRING as edge weights; we divided each value in STRING by 1,000 to scale them between 0 and 1. An edge in this network may be derived from experimental data or computational analysis. Thus, an edge may represent either direct physical binding or indirect functional interaction. Of the 332 viral interactors, 328 were present in this network; REEP6 (Q96HR9), PPIL3 (Q9H2H8), RAB18 (Q9NP72), and FKBP7 (Q9Y680) were missing.

We also computed results for PPI networks from two other sources: the BioGRID database [13], and the high-quality "HI-union" network published by Luck *et al.* [14]. For BioGRID, we considered two versions: (i) all PPIs (including protein complex membership), and (ii) only direct PPIs from yeast two-hybrid (Y2H) screens. For each of these networks, we did not use edge weights and restricted the nodes and edges to those in the largest

Network	# Nodes	# Edges	Edge Weights	Density	# SARS-CoV-2 inter. (/ 333)	# Nbrs. of sources
STRING (400)	18,886	977,789	Y	5.5×10^{-3}	329	12,480
BioGRID	16,595	488,787	N	3.6×10^{-3}	333	9,178
BioGRID-Y2H	12,582	87,801	N	1.1×10^{-3}	271	2,891
HI-union	9,053	64,193	N	1.6×10^{-3}	168	2,031

Table 1. Network statistics. For STRING, the weight cutoff applied is in parentheses. The column titled "# SARS-CoV-2 inter. (/ 333)" shows the number of sources that were in the network. The "# Nbrs. of sources" column shows the number of neighbors of the human proteins that interact with SARS-CoV-2 proteins (i.e., sources) in the given network.

connected component. See Table 1 for statistics of the network size and density.

Drug-protein interactions. We downloaded interactions among drugs and proteins from the DrugBank database (version 5.1.6) [15]. This dataset contained 16,503 drug-protein target pairs among 5,665 drugs and 2,891 target proteins. Limiting the targets to those in the STRING network reduced the number of drugs and targets to 5,589 and 2,769, respectively.

SARS-CoV-2–human A549 AP-MS interactome. We obtained 882 human proteins determined to interact with SARS-CoV-2 proteins by affinity purification followed by mass spectrometry analysis (AP-MS) [16]. This dataset was generated in A549 lung carcinoma cells transduced with lentivirus vectors expressing HA-tagged SARS-CoV-2 proteins. The authors used affinity purification with anti-HA antibodies to isolate stable complexes of human proteins bound to SARS-CoV-2 proteins. Subsequently, they identified and quantified the purified proteins by mass spectrometry.

SARS-CoV-2–human HEK293 AP-MS interactome. We obtained a set of 225 human proteins determined to interact with SARS-CoV-2 by AP-MS [17]. This dataset was generated by analyzing HEK293 embryonic kidney cells transfected with plasmid vectors expressing FLAG-tagged SARS-CoV-2 proteins. Affinity purification with anti-FLAG antibodies was used to isolate stable complexes of human proteins bound to SARS-CoV-2 proteins, and the purified proteins were identified and quantified by mass spectrometry.

SARS-CoV-2–human BioID interactome. We obtained a set of 2,241 human proteins determined to interact transiently or weakly with SARS-CoV-2 proteins by using proximity-dependent biotinylation (BioID) [18]. This dataset was generated by analyzing A549 lung carcinoma cells transduced with lentivirus vectors expressing SARS-CoV-2 proteins fused with a bacterial biotin ligase. The addition of biotin resulted in the biotinylation of host proteins in the proximity of SARS-CoV-2 proteins. Biotinylated proteins were purified and then identified and quantified by mass spectrometry. Compared to interactomes identified by AP-MS, BioID is more capable of identifying weaker interactions in poorly soluble intracellular locations such as membranes and organelles.

Differential protein abundance in SARS-CoV-2–infected iAT2 cells. We obtained a set of 5,665 human proteins determined to have differential abundance in response to SARS-CoV-2 infection [19]. This dataset was generated by infecting induced pluripotent stem cell-derived alveolar epithelial type 2 cells (iAT2) with SARS-CoV-2 and measuring protein abundance by quantitative mass spectrometry at 1, 3, 6, and 24 hours post-infection. The authors compared protein abundance in infected iAT2 cells with that of the uninfected iAT2 controls to obtain differentially-expressed proteins. In our analysis, we used the set of proteins with differential expression (FDR p-value < 0.05) at any of the 1, 3, 6, and 24 hours post-infection.

Differential gene expression in upper airway samples in SARS-CoV-2–infected patients. We obtained three sets of human proteins determined to have differential gene expression in cells infected with respiratory viruses [20]. To generate this dataset, the authors used metagenomic RNA-seq to identify and quantify both human and viral RNA expression in upper airway samples collected from patients with acute respiratory illness. They compared the gene expression values between samples that contained SARS-CoV-2 to uninfected samples in order to obtain differentially-expressed genes. They also identified additional viral infections including SARS-CoV, HRV, Influenza, HMPV, RSV, PIV in patient samples. Comparing SARS-CoV-2 infections with other viral infections and other viral infections with uninfected samples yielded two additional sets of differentially-expressed genes. In our analysis, we used the genes with differential expression (FDR p-value < 0.05) in these three sets obtaining (i) 1,383 genes from SARS-CoV-2-infected cells compared with uninfected samples, (ii) 7,338 genes from SARS-CoV-2-infected cells compared with other viral infections, and (iii) 5,779 genes from other viral infections compared with uninfected samples.

From each of these interactome and differential expression datasets, we removed human proteins used as positive examples in our analysis and the proteins that were not present in the STRING network. This step resulted in 2,080, 807, 212 proteins, respectively, from the interactome datasets and 5,447, 1,293, 6,940, and 5,472 proteins, respectively, from the differential expression datasets. We used Fisher's exact test to estimate the statistical significance of the overlap between the remaining proteins and our top-ranking proteins.

Analyses

Various network propagation methods have been successfully used in diverse applications in systems biology [21]. In particular, we model network propagation using the Regularized Laplacian (RL) [22]. As we describe below ("Methods"), RL has the benefit of two mutually-reinforcing interpretations. On one hand, it can be understood as an optimal labeling of network nodes, when some node labels are known *a priori*. On the other hand, it can be seen as the result of diffusion, i.e., a continuous-time random walk, on the network. Under this second interpretation, we derived a novel mathematical formula for the expected length of the path traversed in the network by the random walker, which we then used to characterize our top-ranking proteins.

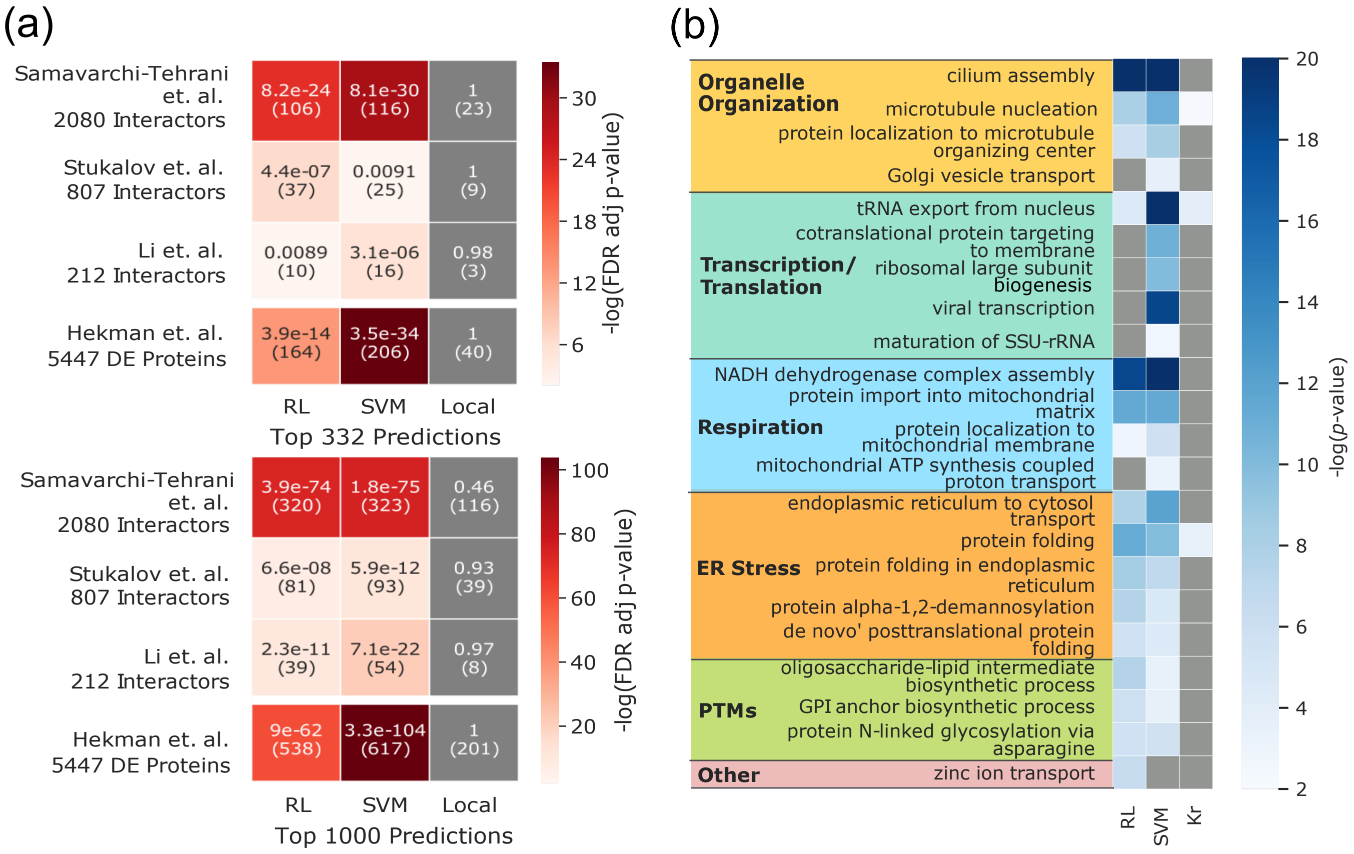

Figure 2. Network propagation results. (a) Heatmap showing the FDR adjusted *p*-value from the hypergeometric test for the overlap between the top-ranking predictions of RL, SVM, and Local and three new experimental datasets of SARS-CoV-2–human protein interactions [18, 17, 16] and one dataset of differentially expressed (DE) proteins after SARS-CoV-2 infection [19]. Each cell displays the FDR-adjusted *p*-value and the number of overlapping proteins in parentheses. A gray cell indicates a *p*-value larger than 0.01. (b) Heatmap summarizing GO biological process terms enriched in top ranking proteins from RL and SVM and human interactors of SARS-CoV-2 proteins (indicated as 'Kr'). We manually grouped the terms into broader categories shown in bold text. A gray cell indicates a *p*-value larger than 0.01. We examine the relevance of these biological processes to SARS-CoV-2 and COVID-19 in "Enriched Biological Processes" in the supplementary results and in "Discussion".

Prioritization of Potential SARS-CoV-2 Interactors

Our underlying hypothesis was that network propagation via methods such as the RL yields a reasonable mechanism for predicting SARS-CoV-2 interactors. Therefore, we applied RL to the set of positive examples to rank the remaining proteins in the STRING network. We also ranked these proteins using multiple other network propagation methods and off-the-shelf classifiers [23, 24, 25, 26]. We used a stratified sampling approach to estimate the statistical significance of the resulting node scores (see "Statistical Significance of Node Scores" in the supplementary methods). The sampling accounted for the possibility that if many sources have high degree, then scores may tend to be large overall in the network. Henceforth, for every method, we only considered proteins in the network that had a *p*-value less than 0.05.

To decide which methods to select for subsequent analyses, we compared them using 5-fold cross validation ("Comparison of Cross-Validation Results" in the supplementary results and Figure S1). RL, random walk with restarts (RWR)[23], and deepNF [26] had the highest values of area under the precision-recall curve followed by SVM and logistic regression. RL achieved marginally worse values of area under the precision-recall curve than RWR and deepNF. We selected one network propagation method (RL) and one supervised classifier (SVM) for the following reasons. We preferred RL over deepNF because the provenance tracing method we developed for RL enabled its results to be more easily interpreted than those for deepNF. Since RL and RWR produced highly similar predictions with a very high Spearman's correlation for the ranking of all proteins ("Overlap among algorithms" in the supplementary results and Figure S2), we selected RL as representative of the two methods. We chose SVM among the two off-the-shelf classifiers since it also had very good performance in cross-validation. We considered the top 332 predictions of RL and SVM that were statistically significant at $p < 0.05$ (File S1), which we refer to as "top-ranking proteins" below.

Three recent publications or preprints have independently discovered physical interactions between SARS-CoV-2 and human proteins [18, 16, 17]. These datasets differed in the type of host cell in which the viral proteins were expressed and the experimental methods used to determine if two proteins interacted. ("Datasets"). The top-ranking proteins for both RL and SVM had significant overlaps with each of the three new datasets, while the results for Local were not statistically significant (p-value > 0.01) (Figure 2(a)). We observed an especially striking overlap with the "proximity interactome" [18]. Approximately one-third of the 332 and 1000 top-ranking proteins computed by RL were present in this dataset of 2,080 interactions (p-value 8.2×10^{-24} and p-value 3.9×10^{-74} respectively).

The corresponding publication used BioID with the fast-acting miniTurbo enzyme [18], a technique that is useful for discovering viral-host protein interactions that take place at intracellular membranes and poorly soluble organelles, which are difficult to profile using classical biochemical purification approaches used in the other publications [11, 16, 17]. Thus, our top-ranking proteins may be members of biological processes that occur in such locations in the cell. These three independent datasets provide strong support of our predictions. Our top-ranking proteins that do not overlap with these resources may interact with viral proteins indirectly and thus would not be captured by assays that test for direct protein-protein interactions.

We additionally tested the overlap between our top predictions and independent experimental datasets identifying differential expression of proteins in response to SARS-CoV-2 infection [19]. As in the previous analysis, we observed that the results for Local were not statistically significant (p-value > 0.01), while both RL and SVM had significant overlaps with differential protein abundance in SARS-CoV-2 infected cells compared with uninfected cells [19](Figure 2(a)). Approximately half of the 332 and 1000 top-ranking proteins computed by RL were present in this dataset of 5,447 differentially expressed proteins (p-value 3.9×10^{-14} and p-value 9×10^{-62} respectively). This high overlap may indicate that these proteins are involved with changes in host protein expression occurring in SARS-CoV-2 infected cells, via either direct or indirect virus-host protein interactions.

In contrast, when we analyzed gene expression measurements in response to SARS-CoV-2 infection [20], we did not observe a significant overlap between our top-ranking proteins and differentially-expressed genes (Figure S8). This result may be attributed to a difference in cell types used for measuring gene expression data, including cells not directly infected by the virus. Moreover, the lack of edges connecting transcription factors to target genes in the PPI network we used may limit the size of the overlap between interactors predicted by RL and SVM with differentially-expressed genes.

We tested for enrichment of Gene Ontology (GO) biological processes (Benjamini-Hochberg corrected p-value ≤ 0.01) among the top-ranking proteins from RL and from SVM, as well as in the interactors of SARS-CoV-2 ("Functional Enrichment" in the supplementary methods). Our top-ranking proteins were enriched in five broad categories of GO biological processes: organelle organization, transcription and translation, respiration, ER stress, and post-translational modifications (Figure 2(b), Figure S5 , and File S2). We examine the relevance of these processes to the viral life cycle in more detail in "Discussion" and in "Enriched Biological Processes" in the supplementary results.

Tracing the Provenance of Top-Ranking Proteins

We can interpret the RL in terms of a continuous-time random walk over the network, which is governed by the internal parameter α. We are interested in the node reached by the walker after a random time that depends on α. The expected number of transitions made by the walker increases with the parameter α ("Analytical Perspective on the RL and Expected Path Length" in the supplementary methods). Hence for larger values of α, the "influence" of the sources is diffused more broadly across the network. To test how this spreading of "influence" affects our results, we varied α over four orders of magnitude from 0.01 to 100 and performed two analyses. First and most importantly, for each top-ranking protein computed by the RL, we developed a systematic procedure to determine the provenance of its score, i.e., which SARS-CoV-2 interactors made the greatest contributions to this score. For our second analysis, we developed a new methodology to select a value of α. We were motivated to do so since we could not use the common practice of choosing the parameter's value based on maximization of cross-validation performance: the AUROC, AUPRC, and precision at 0.3 recall of the RL varied very little with α (Figure S3).

For provenance tracing, we took advantage of the fact that the score computed by the RL for each protein in the network is a linear combination of contributions from source proteins ("Methods"). Therefore, for each protein u in the network, we sorted the source proteins by their relative contributions to the score of u (File S3). Figure 3(a)–(d) provide illustrative examples of the practical usefulness of provenance tracing. We used a value of $\alpha = 3.4$ to obtain these results. We present our method for selecting α at the end of this section.

In Figure 3(a), we display the top 332 ranking proteins computed by the RL that are annotated to the enriched GO term "protein folding in endoplasmic reticulum". For each such protein, we also show all the sources that interact with it as well as the viral proteins that in turn interact with the sources. This network is complex and difficult to understand. In contrast, in Figure 3(b), we connect each top-ranking protein only to the two source proteins that contribute the most to its score. This simplified network considerably facilitates the interpretation and rationalization of the RL's predictions. Figure 3(c,d) are similar in nature and correspond to the enriched term "cilium assembly". We return to the biological insights present in these networks in "Discussion".

Next, we considered the effect of α on the amount of diffusion in the network. When α was very small, e.g., 0.01, we expected the highest contributing sources to be direct neighbors of top-ranking proteins. As α increased, and the random walker traversed longer paths in the network, we expected more of the highest contributors to not be directly connected by an edge to top-ranking proteins. Contrary to our expectations, we found that for every value of α and for every top-ranking protein u (till a rank of 1,000), the source protein with the highest contribution to u's score was always a neighbor of u. Even when we considered the second and third highest contributors, we found that they were more than one edge away for as few as 2% of the top-ranking proteins for $\alpha = 0.01$. This number increased only to 25% for $\alpha = 100$.

The STRING network includes both direct, physical and indirect, functional PPIs. Therefore, we sought to see if this trend in the provenance analysis held for networks with only physical interactions corresponding to direct binding and indirect protein complex membership. We repeated the analyses up to this point on three other PPI networks: BioGRID, BioGRID-Y2H, and HI-union ("Methods"). For BioGRID, the results were comparable to those for STRING. The highest contributor was always a neighbor, except for $\alpha \geq 10$ where up to 3% of nodes received most of their score from a source more than one edge away. The second and third highest contributor was more than one step away for as few as 8% of top-ranking nodes for $\alpha = 0.01$, and up to 41% for $\alpha = 100$. For BioGRID-Y2H and HI-union, which are smaller, sparser networks with only direct PPIs, only 300–400 nodes had scores that were statistically significant at the 0.05 level. The highest contributing source was more than one step away for as many as 10–30% of the top-ranking nodes, even for $\alpha = 10$. For the second highest contributor, this percentage jumped to more than 50% for $\alpha = 0.01$ itself.

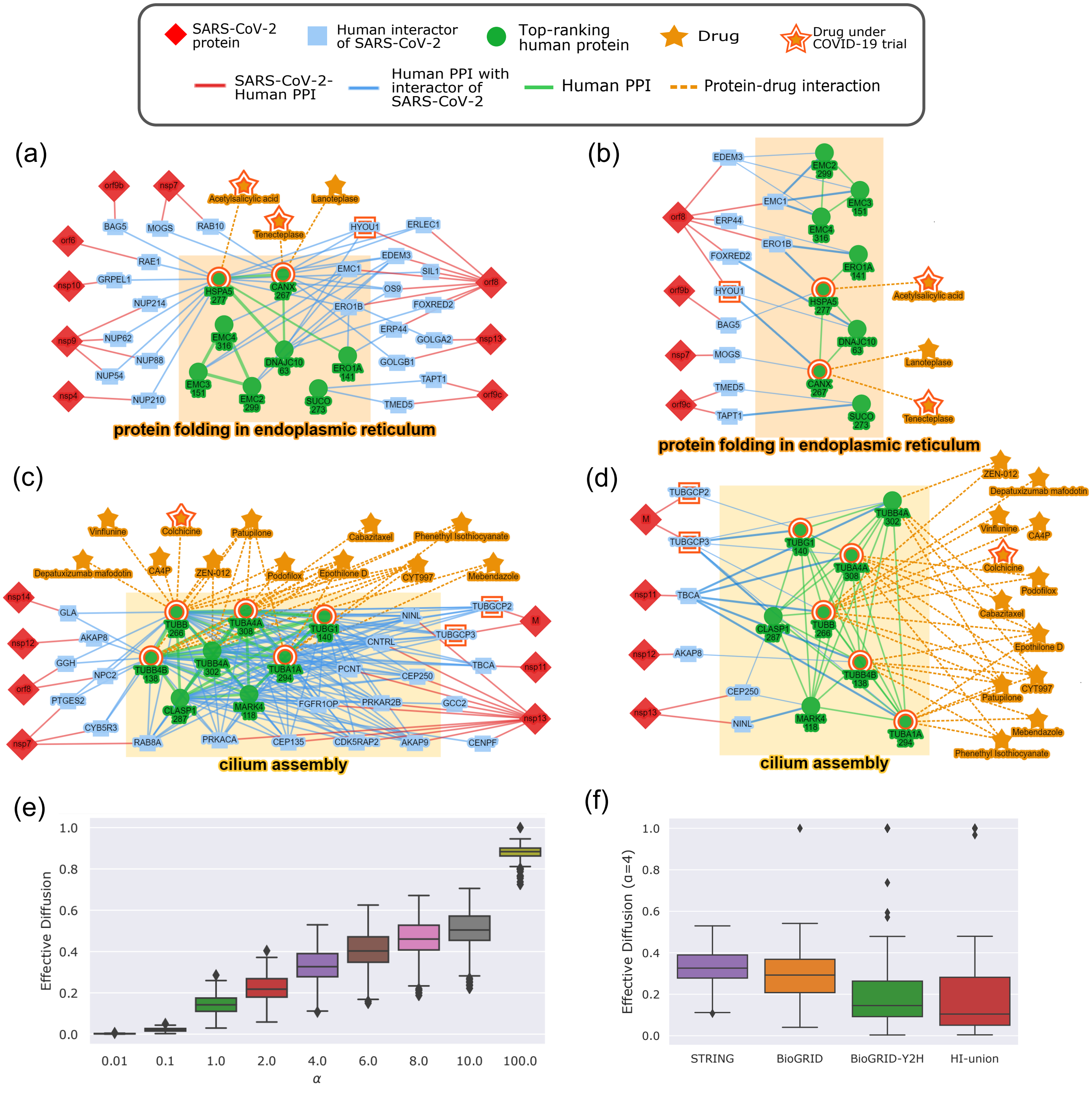

Figure 3. Provenance tracing results and illustrative examples of networks. (a) Network of the top 332 ranking proteins for RL (green nodes) that are annotated to the enriched term "protein folding in ER". For each top-ranking protein, we display its connections with all neighboring SARS-CoV-2 interactors. (b) The same network as in (a) except that we display only the top-two contributing SARS-CoV-2 interactors for each top-ranking protein. (c) Network of the top 332 ranking proteins for RL (green nodes) that are annotated to the enriched term "cilium assembly." (d) The same network as in (c) except that we display only the top-two contributing SARS-CoV-2 interactors for each top-ranking protein. In all four network visualizations, the number below the name of a green protein is its rank as computed by the RL. Proteins discussed in the text are highlighted with a red border. In (a,c), we removed STRING edges with weight < 700 to simplify the visualization. In (c,d), we removed drugs that promote clotting. (e) Distribution of effective diffusion for the top 332 ranking proteins for different values of α. (f) The same distribution as (e) except comparing different networks with α = 4.0.

To further characterize the contribution of non-neighboring sources, we defined the *effective diffusion* to a protein u as the fraction of its score $s(u)$ that arose from the non-direct neighbors of u that were also SARS-CoV-2 interactors. As expected, the effective diffusion to the top-ranking proteins increased with α with values close to zero for $\alpha = 0.01$ and a median of 0.88 for $\alpha = 100$ (Figure 3(e)). We concluded that the neighbours of the sources received non-trivial contributions to their RL scores from indirectly-connected sources only for values of $\alpha = 1$ and higher.

We repeated these experiments for BioGRID, BioGRID-Y2H, and HI-union (see Figure 3(f) and Figure S9). BioGRID maintained fairly similar results to STRING. On the other hand, for the other two networks, their effective diffusion values were quite a bit smaller (difference from STRING about 0.2 on average). Taken together, these results suggest that in the sparser networks (BioGRID-Y2H and HI-union), a top-ranking protein has fewer sources as direct neighbours than in the denser networks (STRING and BioGRID) but a larger proportion of its score arises from these adjacent sources.

These results motivated us to test a different method for selecting an appropriate value of α for downstream analysis. As mentioned earlier, we mathematically derived a new expression for the expected value of the path length of the random walker ("Analytical Perspective on the RL and Expected Path Length" in the supplementary methods). To our knowledge, no such formula is known for the interpretation of the RL as a continuous-time Markov chain. This value depended on α, the topology of the network, and which proteins interacted with SARS-CoV-2. We computed the expected path length for different values of α (Table S1). Independently, we computed the distribution of path lengths in the network from SARS-CoV-2 interactors to every other protein (Figure S10). The median number of edges in these paths was three. Therefore, we set the value of $\alpha = 3.4$ for which the expected path length of the random walker was 3.04 (Table S1). The median effective diffusion for this value of α was around 0.3. We used this value of α to generate the results presented in this work.

Discussion

The COVID-19 pandemic and its medical and economic impact have created an urgent challenge for biomedical researchers to understand infection mechanisms used by SARS-CoV-2 and to develop therapeutics against the disease [27]. A manifestation of this community response is the first protein-protein interactome associated with the SARS-CoV-2-human interface [11]. This set of human proteins reported to interact with SARS-CoV-2 is likely to have both false positives and false negatives due to the properties of the proteomic screening pipeline used.

In this work, we sought to further extend the results of this study to significantly expand the resources available to the COVID-19 community by producing an extended set of putative SARS-CoV-2 interactors. Comparison of our results with independently-generated SARS-CoV-2-human protein interaction networks [18, 17, 16] provides substantial experimental support for our predictions. We note that complementary efforts are based on protein structures [28], observational studies of treatments being administered to patients [29], shortest paths in protein networks [30], propagation in protein networks with predicted SARS-CoV-2 interactors [31], and exploratory analyses of virus-host-drug networks [32].

A notable new feature of our methodology is tracing the provenance of each of our predictions back to the most informative experimental sources [10]. In principle, the RL computes scores by integrating over all paths in the network. We were surprised to see that the top-contributing sources were invariably direct neighbours of the top-ranking predictions in the STRING network. A partial explanation for this trend may be the fact that as many as 5,331 proteins in the STRING network were direct neighbors of at least one source protein, even when we considered only interactions with weight at least 0.9 (the STRING database deems edges with such weights to be of "very high quality"). Thus, the structure of the STRING network and central location of sources within it may cause the RL both to give high ranks only to direct neighbors of sources and to channel propagation primarily along these direct connections. We stress that using only the interactions between sources and their neighbors in the network does not result in high-quality predictions, as evidenced by the relatively poor cross-validation performance of the Local algorithm. Thus, the integration of multiple paths by the RL plays a key role in prioritizing which neighbors of the sources are more likely to be potential interactors of SARS-CoV-2 proteins than others.

COVID-19 research has focused disproportionately on a small set of human proteins [33]. Our research has the potential to expand the repertoire of host proteins that are studied in the context of COVID-19 and thereby open new directions of study of the disease. The cellular processes in which our top-ranking proteins participate suggest how the virus may infect human cells. We discuss two illustrative examples of the type of insights provided by our approach, highlighting several proteins targeted by drugs that are already in clinical trials for COVID-19. We remind the reader that we computed functions enriched in the top-ranking proteins, performed the provenance analysis independently, and then integrated the results in the protein networks we visualized.

The Role of Endoplasmic Reticulum Stress, HSPA5, and Anti-Clotting Drugs

Our analysis points to a connection among interactors of SARS-CoV-2, proteins involved in endoplasmic reticulum (ER) stress, and anti-clotting drugs ((Figure 3(a,b))). The GO biological process "protein folding in endoplasmic reticulum" was enriched in the top-ranking proteins (p-value 4.32×10^{-9} for RL and 0.28 for interactors of SARS-CoV-2). HSPA5, also referred to as glucose regulated protein (GRP78) or immunoglobulin binding protein (BiP) in the literature, is evolutionarily conserved from prokaryotes to humans [34]. It has a repertoire of functions associated with ER stress response. HSPA5 is usually localized in the ER. When the ER is stressed, HSPA5 can translocate to the cell surface, the nucleus and mitochondria [35, 36]. On the cell surface, HSPA5 plays a multi-functional role in cell proliferation, cell viability, apoptosis, and regulation of innate and adaptive immunity [36, 37].

HSPA5 has been proposed as a universal target for human diseases [38]. It has increasingly well-documented essential interactions and activities during viral infections. In particular, the role of HSPA5 in viral entry and pathogenesis has been widely investigated. SARS-CoV infection has been shown to lead to ER stress and the up-regulation of HSPA5 [39, 40]. The S protein of SARS-CoV can induce transcriptional activation of HSPA5 [40]. This protein can serve as a point of attachment for both MERS-CoV and bat coronavirus (bCoV HKU9) [41]. Both Zika virus and Japanese encephalitis virus use HSPA5 to prevent apoptosis and to help in viral replication [42]. A recent molecular docking study has predicted HSPA5 as a potential receptor for the SARS-CoV S

protein [43]. The observed expression *in vitro* of HSPA5 in airway epithelial cells suggests that it may serve as an additional receptor for SARS-CoV-2 in these cells [44]. Based on our network-based analysis and support in the literature, we hypothesize that HSPA5 may serve as a co-receptor, a point of viral attachment, or aid in viral entry of SARS-CoV-2.

Blood hypercoagulability is reported to be common among COVID-19 patients [45]. Top-ranking proteins HSPA5 and CANX act as chaperones for pro-coagulant proteins such as Factor V and Factor VIII. Once Factor VIII is secreted, it binds to another pro-coagulant protein von Willebrand factor (vWF) to prevent degradation of clots [46]. Although Factor V, Factor VIII, and vWF are not among the top-ranking proteins and thus do not appear in Figure 3(a,b), this network is suggestive of mechanisms that SARS-CoV-2 may use to cause abnormal blood coagulation.

Anti-coagulant drugs that interact with HSPA5 or CANX include Tenecteplase, a third generation plasminogen activating enzyme and the investigational drug Lanoteplase, which is a serine protease that binds to fibrin leading to the formation of plasmin [47], an enzyme that breaks clots. Lanoteplase is a second-generation derivative of Alteplase, and a third generation derivative of recombinant plasminogen. It is notable that there are clinical trials for Tenecteplase (ClinicalTrials.gov, NCT04558125, NCT04505592) and Alteplase (ClinicalTrials.gov, NCT04357730, NCT04640194) to test their effectiveness in treating COVID-19. Aspirin, also present in (Figure 3(a,b)), binds to and inhibits the ATPase activity of HSPA5 [48]. Aspirin is currently involved in 16 clinical trials (ClinicalTrials.gov), with one testing the effects of aspirin at various levels of COVID-19 severity (NCT04365309), and another testing whether early treatment of COVID-19 patients with aspirin and vitamin D can inhibit the production of blood clots and decrease rates of hospitalization (NCT04363840).

Cilium Assembly and Tubulin-Modulating Drugs

GO biological processes related to cilia were significantly enriched in the top-ranking RL and SVM predictions. An example is "cilium assembly" (p-value 6.84×10^{-26} for RL vs. 0.31 in the human interactors of SARS-CoV-2. Many proteins annotated to this term belong to the tubulin family, which are components of microtubules. The SARS-CoV-2 M protein binds to two γ-tubulins (TUBGCP2 and TUBGCP3), which interact with several α- and β-tubulins among the top 332 predictions (Figure 3(c,d)). Microtubules are polymers that provide shape and structure to eukaryotic cells and are necessary in cell transport and cell division, among other functions [49]. α- and β-tubulins compose microtubule filaments, while γ-tubulins connect them to the microtubule organizing center.

Viruses commonly utilize microtubules for cellular entry, intra-cellular trafficking, and exit from cells [50]. For instance, the S protein of human α-coronavirus interacts with tubulin α and β chains [51], suggesting that tubulin may be involved in the transport and localization of the S protein and its assembly into virions [51]. Relevant to SARS-CoV-2, microtubules are the primary structural component of cilia, which line epithelial cells in the respiratory tract and are responsible for the transport of mucus out of cells [52]. The ACE2 receptor that SARS-CoV-2 uses to enter cells appears to be expressed primarily on the cilia of respiratory tract epithelial cells [53, 54], further implicating microtubules in viral infection. The combination of high expression levels of ACE2 and the presence of cilia may also explain the detection of the virus in multiple organs [55] and the deleterious effect of COVID-19 on the renal, gastroinstestinal, and olfactory systems [56]. The drugs that target Tubulin proteins (Figure 3(c,d)) are mostly anti-mitotic agents, which are being investigated as anti-cancer therapeutics. It is notable that 26 ongoing clinical trials (ClinicalTrials.gov) are testing the effectiveness of Colchicine against COVID-19.

Our work also sets the stage for follow-up analyses on SARS-CoV-2. Integrating new datasets of SARS-CoV-2-human protein interactions [18, 17, 16] and human proteins whose deletion inhibits viral replication [57, 58] with other omics data using our methods and with orthogonal analysis techniques promises to predict more biologically meaningful networks and processes impacted by the virus. In particular, single-cell RNA-seq data offer many opportunities to examine cellular heterogeneity and context-specific interactions.

Potential Implications

The approach we advocate here is inspired by the general framework of producing explanations for machine learning methods [59]. This area of "explanations" of predictions is receiving strong interest because of deep learning. While the idea has previously been studied in graphical models [60], most machine learning methods are not fully interpretable by the fairly strict definition of Kasif and Roberts [10]: tracing each prediction to the experimental evidence that supports it. This notion of explanation is a special but particularly important case for computational genomics and systems biology.

Causal perturbations [60] provide a general approach for producing explanations of this type for virtually any predictive model. Consider a model with experimental evidence that a gene g performs a function f. We perturb the variable associated with the gene, e.g. we change the probability $\Pr(g \text{ performs } f) = 1$ to $\Pr(g \text{ performs } f) = 0$. We then compute the change in probability of every other variable in the model due to this perturbation in order to assess the importance of this particular gene-function pair.

For network propagation, this idea yields the special case discussed in this work that is amenable to very efficient computation. Our strategy for tracing provenance extends to any algorithm that makes predictions using a linear combination of evidence such as logistic regression and GeneMania [61]. In particular, it is applicable to the large number of random-walk-based methods that have been developed for predicting disease genes or annotations to GO terms [62, 63, 64, 65].

An important future line of research will be to develop provenance tracing techniques for other classes of network-based methods such as Markov random fields (MRFs) [66, 67] and mincut based methods [6, 68]. For MRFs, we can apply the general perturbation-based method described above. For mincut-based methods, it is possible to recalculate the cut for any single change in experimental data using dynamic data structures [69]. Thus, the provenance tracing approach that we advocate here has many natural follow-ups that we expect to be studied by the community in the future.

It remains to be seen whether the trends we observed on the contributions from direct neighbors generalize to these methods and to annotations of terms in the Gene Ontology or the Human Phenotype Ontology terms. In general, it is quite likely that sources that are not direct neighbors may make substantial contributions to scores. In these cases, new algorithmic developments may be required to trace the paths by which the sources

spread their influence to a given node.

Our work provides significant new data and software resources to the COVID-19 community. Three properties of our results facilitate their use by experimentalists who are seeking to obtain new insights into the pathogenesis of this disease. First, the prioritized list of predicted interactors of SARS-CoV-2 (File S1) contains druggable targets that may be promising to study further. Second, our provenance analysis provides the rationale underlying each prediction by directly linking to the relevant experimental input. Third, the viral-human protein interaction networks corresponding to enriched GO terms (Figure 3 and Figure S6) are available for visualization and download on GraphSpace (http://graphspace.org/graphs/?query=tags:2021-sarscov2-network-analysis). Examination of these networks provides further context for the predictions.

We conclude by noting that our methodology is general purpose and easy to generalise to a new virus. The software requires a dataset of host proteins that interact with the virus and an interaction network among the host proteins themselves. The virus-host network may be determined experimentally [11]. If such a dataset is not available, a user can predict the network computationally from the sequence of the viral genes and interaction networks for phylogenetically similar viruses [70]. Subsequently, a user can apply network propagation to predict additional human proteins and biological processes that may be targeted by the virus.

Methods

Algorithms

To facilitate the complete reproducibility of our results, we now describe the RL algorithm that we use for label propagation and prediction. We present the other methods that we use (GeneMANIA, SinkSource, RWR, Local, deepNF, the Support Vector Machine, and Logistic Regression) and implementation details in "Other Algorithms" in the supplementary methods. We are given a weighted, undirected network $G = (V, E, w)$, where each node in V is a human protein, each edge (u, v) represents an interaction between proteins u and v, and $w : E \to (0, 1]$ is a function specifying the weight of each edge in E. Informally, the weight of an edge indicates our confidence in the experimental data supporting the corresponding protein-protein interaction. We are also given a set $P \in V$ of positive examples consisting of the human proteins that interact with SARS-CoV-2 proteins [11]. Each node in G is a human protein and each edge represents a physical or functional interaction between two proteins. We seek to compute a score vector $\vec{s} \in \mathbb{R}^n$, where n is the number of nodes in G. For every node v, the score $s(v)$ in this vector indicates our confidence that node v either physically interacts with or is functionally linked to a SARS-CoV-2 protein.

Regularized Laplacian [22]. Given a parameter $\alpha > 0$, we compute $\vec{s}$ using the following steps:

i. Define a label vector $\vec{y}$ over the nodes in G where $y(u) = 1$ if node u is in P and $y(u) = 0$, otherwise.
ii. Define $W \in \mathbb{R}^{n\times n}$ as the adjacency matrix of G with edge weights, i.e., the entry in row u and column v of W equals w_{uv} if (u, v) is an edge in G and 0, otherwise.
iii. Define D as a diagonal matrix with $D_{uu} = \sum_v w_{uv}$, for every node u in G.
iv. Compute the $\mathbb{R}^{n\times n}$ matrix $\tilde{W} = D^{-1/2} W D^{-1/2}$, which denotes the normalized network.
v. Compute the Laplacian of G as $\tilde{L} = \tilde{D} - \tilde{W}$, where we define $\tilde{D}$ to be a diagonal matrix with $\tilde{D}_{uu} = \sum_v \tilde{w}_{uv}$.
vi. Compute the vector $\vec{s} = (I + \alpha\tilde{L})^{-1}\vec{y}$.

The RL was introduced by Zhou and Schölkopf. Since then, several variations of this method have been published. The version we use is identical to the strategy used by Fouss *et al.* [22]. We provide the intuition behind the resulting RL matrix (i.e., $(I + \alpha\tilde{L})^{-1}$) and discuss its properties in "Analytical Perspective on the RL and Expected Path Length" in the supplementary methods. In particular, we derive an expression for the expected path length of the continuous-time Markov chain corresponding to the RL. As far as we know, this mathematical analysis has not previously been published.

Tracing the Provenance of Prediction Scores

Let K denote the RL matrix $(I + \alpha\tilde{L})^{-1}$. We remind the reader that the RL algorithm ranks proteins based on diffusion scores that associate a node u in the network with a diffusion score $s(u)$, where $s(u) = \sum_{v\in P} K_{uv}$, where v ranges over the set P of all SARS-CoV-2 interactors. For every protein u, we sorted the proteins in P in decreasing order of the values of K_{uv}, where v ranged over P. In the manner, we ranked the experimentally determined interactors that in decreasing order of their contributions to each node's diffusion score. This analysis is important for tracing the provenance of computational predictions to their experimental sources [10].

Availability of Source Code and Requirements

- Project name: SARS-CoV-2-network-analysis
- Project home page: https://github.com/Murali-group/SARS-CoV-2-network-analysis
- Operating system(s): Platform independent (tested and applied on Linux and Mac OS)
- Programming language: Python
- Other requirements: Please see https://github.com/Murali-group/SARS-CoV-2-network-analysis/blob/master/requirements.txt
- License: GNU General Public License (GPL) v3

Availability of supporting data and materials

We used publicly available datasets for our analysis. We downloaded these data from the respective publications or websites. We also provide the following supplementary files.

File S1: The prediction rank and p-value computed by RL and SVM for each human protein on the STRING network, the list of drugs that target the protein (when this information is available in DrugBank), and the closest SARS-CoV-2 interactor and SARS-CoV-2 protein. For the last piece of information, we computed the shortest weighted path, where we defined the weight of a path to be the sum of the absolute value of the base-10 logarithm of the weights of the edges in the path.

File S2: Enrichment results for RL, SVM and the viral interactors on GO biological processes.

File S3: Provenance tracing matrix of contributions to the network propagation score from each SARS-CoV-2 interactor to every top-ranking protein.

Declarations

Abbreviations

AP-MS: affinity purification followed by mass spectrometry analysis; AUPRC: area under the precision-recall curve; AUROC: area under the receiver-operator characteristic curve; bCoV: bat coronavirus; COVID-19: novel coronavirus disease 2019; BioID: proximity-dependent biotinylation; BiP: immunoglobulin binding protein; ER: endoplasmic reticulum; GM: GeneMania; GPL: General Public License; GO: Gene Ontology; GRP: glucose regulated protein; HIV-1: human immunodeficiency virus 1; HSV-1: herpes simplex virus type 1; KEGG: Kyoto Encyclopedia of Genes and Genomes; LogReg: Logistic Regression; MERS: Middle East respiratory syndrome; NSF: National Science Foundation; PPMI: Positive Pointwise Mutual Information; RWR: random walk with restarts; RL: Regularized Laplacian; SARS: severe acute respiratory syndrome; SARS-CoV-2: severe acute respiratory syndrome coronavirus 2; SS: SinkSource; SVM: Support Vector Machine; vWF: von Willebrand factor; USDA-NIFA: United States Department of Agriculture National Institute of Food and Agriculture

Consent for Publication

Not applicable.

Competing Interests

The authors declare that they have no competing interests.

Funding

TMM acknowledges support from National Science Foundation (NSF) grants DBI-1759858 and MCB-1817736. KA acknowledges support from the Genetics, Bioinformatics, and Computational Biology program at Virginia Tech. JK acknowledges support from NSF grant CCF-2029543. MC acknowledges support from NSF grant CNS-1618207. CMDS acknowledges support from the Hariri Institute and the Department of Biomedical Engineering at Boston University. PR acknowledges support from NSF grant CBET-1510920 and USDA-NIFA grant 2018-07578. PR and TMM acknowledge support from the Computational Tissue Engineering Graduate Education Program at Virginia Tech.

Author Contributions

TMM and SK proposed the study. TMM, SK, MC, JL, SD, MK, and JK contributed computational ideas. JL was the primary author of the software and led the computational analysis, with significant inputs from KA, NT, and CMDS. All authors analyzed the results. TMM, MC, PR, and SK wrote the paper with contributions and revisions from all authors. All the authors read and approved the final manuscript.

Acknowledgments

The authors wish to thank S. Alabdullatif, S. Alshuaib, M. Iennaco, M. Kouzminov, S. Murthy, S. Makwana, N. Naguib, C. Tagliettii, and M. Zanna for exploratory research on this data and insightful and thought-provoking analysis. We also thank Roded Sharan, Noga Alon, Dan Lancour and Rich Roberts for discussions that helped formulate the techniques and ideas we used in this paper.

References

1. Vazquez A, Flammini A, Maritan A, Vespignani A. Global protein function prediction from protein-protein interaction networks. Nat Biotechnol 2003;21(6):697–700.
2. Letovsky S, Kasif S. Predicting protein function from protein/protein interaction data: a probabilistic approach. Bioinformatics 2003;19 Suppl 1:i197–204.
3. Karaoz U, Murali TM, Letovsky S, Zheng Y, Ding C, Cantor CR, et al. Whole-genome annotation by using evidence integration in functional-linkage networks. Proceedings of the National Academy of Sciences of the United States of America 2004 3;101(9):2888–2893. http://dx.doi.org/10.1073/pnas.0307326101.
4. Deng M, Chen T, Sun F. An integrated probabilistic model for functional prediction of proteins. J Comput Biol 2004;11(2-3):463–75.
5. Fraser AG, Marcotte EM. A probabilistic view of gene function. Nat Genet 2004;36(6):559–64.
6. Murali TM, Wu CJ, Kasif S. The Art of Gene Function Prediction. Nature Biotechnology 2006;12:1474–1475.
7. Ideker T, Sharan R. Protein networks in disease. Genome research 2008 4;18(4):644–652. http://dx.doi.org/10.1101/gr.071852.107.
8. Leiserson MD, Vandin F, Wu HT, Dobson JR, Eldridge JV, Thomas JL, et al. Pan-cancer network analysis identifies combinations of rare somatic mutations across pathways and protein complexes. Nat Genet 2015 Feb;47(2):106–114.
9. van Dijk D, Sharma R, Nainys J, Yim K, Kathail P, Carr AJ, et al. Recovering Gene Interactions from Single-Cell Data Using Data Diffusion. Cell 2018 07;174(3):716–729.
10. Kasif S, Roberts RJ. We need to keep a reproducible trace of facts, predictions, and hypotheses from gene to function in the era of big data. PLoS Biol 2020 11;18(11):e3000999.
11. Gordon DE, Jang GM, Bouhaddou M, Xu J, Obernier K, White KM, et al. A SARS-CoV-2 protein interaction map reveals targets for drug repurposing. Nature 2020;.
12. Szklarczyk D, Morris JH, Cook H, Kuhn M, Wyder S, Simonovic M, et al. The STRING Database in 2017: Quality-Controlled Protein-Protein Association Networks, Made Broadly Accessible. Nucleic Acids Research 2016;45(D1):D362–D368. https://doi.org/10.1093/nar/gkw937.
13. Oughtred R, Rust J, Chang C, Breitkreutz BJ, Stark C, Willems A, et al. The BioGRID database: A comprehensive biomedical resource of curated protein, genetic, and chemical interactions. Protein Science 2021;30(1):187–200. https://onlinelibrary.wiley.com/doi/abs/10.1002/pro.3978.
14. Luck K, Kim DK, Lambourne L, Spirohn K, Begg BE, Bian W, et al. A reference map of the human binary protein interactome. Nature 2020;580(7803):402–408.
15. Wishart DS, Feunang YD, Guo AC, Lo EJ, Marcu A, Grant JR, et al. DrugBank 5.0: A major update to the DrugBank

database for 2018. Nucleic Acids Research 2018;.
16. Stukalov A, Girault V, Grass V, Karayel O, Bergant V, Urban C, et al. Multilevel proteomics reveals host perturbations by SARS-CoV-2 and SARS-CoV. Nature 2021 06;594(7862):246–252.
17. Li J, Guo M, Tian X, Wang X, Yang X, Wu P, et al. Virus-Host Interactome and Proteomic Survey Reveal Potential Virulence Factors Influencing SARS-CoV-2 Pathogenesis. Med (N Y) 2021 Jan;2(1):99–112.
18. Samavarchi-Tehrani P, Abdouni H, Knight JDR, Astori A, Samson R, Lin ZY, et al. A SARS-CoV-2 – host proximity interactome. bioRxiv 2020;https://www.biorxiv.org/content/early/2020/09/04/2020.09.03.282103.
19. Hekman RM, Hume AJ, Goel RK, Abo KM, Huang J, Blum BC, et al. Actionable Cytopathogenic Host Responses of Human Alveolar Type 2 Cells to SARS-CoV-2. Mol Cell 2020 12;80(6):1104–1122.
20. Mick E, Kamm J, Pisco AO, Ratnasiri K, Babik JM, Calfee CS, et al. Upper airway gene expression differentiates COVID-19 from other acute respiratory illnesses and reveals suppression of innate immune responses by SARS-CoV-2. medRxiv 2020 May;.
21. Cowen L, Ideker T, Raphael BJ, Sharan R. Network propagation: a universal amplifier of genetic associations. Nature Reviews Genetics 2017 Sep;18(9):551–562.
22. Fouss F, Francoisse K, Yen L, Pirotte A, Saerens M. An experimental investigation of kernels on graphs for collaborative recommendation and semisupervised classification. Neural Networks 2012;31:53 – 72. http://www.sciencedirect.com/science/article/pii/S0893608012000822.
23. Page L, Brin S, Motwani R, Winograd T. The PageRank Citation Ranking: Bringing Order to the Web. Stanford InfoLab; 1999.
24. Mostafavi S, Ray D, Warde-Farley D, Grouios C, Morris Q. GeneMANIA: a Real-Time Multiple Association Network Integration Algorithm for Predicting Gene Function. Genome Biology 2008;9(Suppl 1):S4. http://dx.doi.org/10.1186/gb-2008-9-s1-s4.
25. Murali TM, Dyer MD, Badger D, Tyler BM, Katze MG. Network-based prediction and analysis of HIV dependency factors. PLoS computational biology 2011 9;7(9):e1002164+. http://dx.doi.org/10.1371/journal.pcbi.1002164.
26. Gligorijević V, Barot M, Bonneau R. deepNF: Deep Network Fusion for Protein Function Prediction. Bioinformatics 2018;34(22):3873–3881. https://doi.org/10.1093/bioinformatics/bty440.
27. Guy RK, DiPaola RS, Romanelli F, Dutch RE. Rapid repurposing of drugs for COVID-19. Science 2020 05;368(6493):829–830.
28. Wu C, Liu Y, Yang Y, Zhang P, Zhong W, Wang Y, et al. Analysis of therapeutic targets for SARS-CoV-2 and discovery of potential drugs by computational methods. Acta Pharmaceutica Sinica B 2020;.
29. Vaduganathan M, Vardeny O, Michel T, McMurray JJ, Pfeffer MA, Solomon SD. Renin–angiotensin–aldosterone system inhibitors in patients with Covid-19. New England Journal of Medicine 2020;382(17):1653–1659.
30. Zhou Y, Hou Y, Shen J, Huang Y, Martin W, Cheng F. Network-based drug repurposing for novel coronavirus 2019-nCoV/SARS-CoV-2. Cell discovery 2020;6(1):1–18.
31. Zhang Y, Zeng T, Chen L, Ding S, Huang T, Cai YD. Identification of COVID-19 Infection-Related Human Genes Based on a Random Walk Model in a Virus–Human Protein Interaction Network. BioMed research international 2020;2020.
32. Sadegh S, Matschinske J, Blumenthal DB, Galindez G, Kacprowski T, List M, et al. Exploring the SARS-CoV-2 virus-host-drug interactome for drug repurposing. Nat Commun 2020 07;11(1):3518.
33. Stoeger T, Nunes Amaral LA. COVID-19 research risks ignoring important host genes due to pre-established research patterns. Elife 2020 11;9.
34. Lee AS. Glucose-regulated proteins in cancer: molecular mechanisms and therapeutic potential. Nat Rev Cancer 2014 Apr;14(4):263–276.
35. Zhang Y, Liu R, Ni M, Gill P, Lee AS. Cell surface relocalization of the endoplasmic reticulum chaperone and unfolded protein response regulator GRP78/BiP. J Biol Chem 2010 May;285(20):15065–15075.
36. Tsai YL, Ha DP, Zhao H, Carlos AJ, Wei S, Pun TK, et al. Endoplasmic reticulum stress activates SRC, relocating chaperones to the cell surface where GRP78/CD109 blocks TGF-β signaling. Proc Natl Acad Sci USA 2018 05;115(18):E4245–E4254.
37. Ni M, Zhang Y, Lee AS. Beyond the endoplasmic reticulum: atypical GRP78 in cell viability, signalling and therapeutic targeting. Biochem J 2011 Mar;434(2):181–188.
38. Booth L, Roberts JL, Cash DR, Tavallai S, Jean S, Fidanza A, et al. GRP78/BiP/HSPA5/Dna K is a universal therapeutic target for human disease. J Cell Physiol 2015 Jul;230(7):1661–1676.
39. DeDiego ML, Nieto-Torres JL, Jiménez-Guardeño JM, Regla-Nava JA, Alvarez E, Oliveros JC, et al. Severe acute respiratory syndrome coronavirus envelope protein regulates cell stress response and apoptosis. PLoS Pathog 2011 Oct;7(10):e1002315.
40. Chan CP, Siu KL, Chin KT, Yuen KY, Zheng B, Jin DY. Modulation of the unfolded protein response by the severe acute respiratory syndrome coronavirus spike protein. J Virol 2006 Sep;80(18):9279–9287.
41. Chu H, Chan CM, Zhang X, Wang Y, Yuan S, Zhou J, et al. Middle East respiratory syndrome coronavirus and bat coronavirus HKU9 both can utilize GRP78 for attachment onto host cells. J Biol Chem 2018 07;293(30):11709–11726.
42. Lyoo HR, Park SY, Kim JY, Jeong YS. Constant up-regulation of BiP/GRP78 expression prevents virus-induced apoptosis in BHK-21 cells with Japanese encephalitis virus persistent infection. Virol J 2015 Feb;12:32.
43. Ibrahim IM, Abdelmalek DH, Elshahat ME, Elfiky AA. COVID-19 spike-host cell receptor GRP78 binding site prediction. J Infect 2020 05;80(5):554–562.
44. Aguiar JA, Tremblay BJM, Mansfield MJ, Woody O, Lobb B, Banerjee A, et al. Gene expression and in situ protein profiling of candidate SARS-CoV-2 receptors in human airway epithelial cells and lung tissue. bioRxiv 2020;https://www.biorxiv.org/content/early/2020/04/12/2020.04.07.030742.
45. Terpos E, Ntanasis-Stathopoulos I, Elalamy I, Kastritis E, Sergentanis TN, Politou M, et al. Hematological findings and complications of COVID-19. Am J Hematol 2020 Apr;.
46. Kaufman RJ, Pipe SW, Tagliavacca L, Swaroop M, Moussalli M. Biosynthesis, assembly and secretion of coagulation factor VIII. Blood Coagul Fibrinolysis 1997 Dec;8 Suppl 2:3–14.
47. Flemmig M, Melzig MF. Serine-proteases as plasminogen activators in terms of fibrinolysis. J Pharm Pharmacol 2012 Aug;64(8):1025–1039.
48. Deng WG, Ruan KH, Du M, Saunders MA, Wu KK. Aspirin and salicylate bind to immunoglobulin heavy chain binding

protein (BiP) and inhibit its ATPase activity in human fibroblasts. FASEB J 2001 Nov;15(13):2463–2470.
49. Nogales E. Structural insights into microtubule function. Annu Rev Biochem 2000;69:277–302.
50. Greber UF, Way M. A superhighway to virus infection. Cell 2006 Feb;124(4):741–754.
51. Rüdiger AT, Mayrhofer P, Ma-Lauer Y, Pohlentz G, Müthing J, von Brunn A, et al. Tubulins interact with porcine and human S proteins of the genus Alphacoronavirus and support successful assembly and release of infectious viral particles. Virology 2016 10;497:185–197.
52. Satir P, Christensen ST. Overview of structure and function of mammalian cilia. Annu Rev Physiol 2007;69:377–400.
53. Lee IT, Nakayama T, Wu CT, Goltsev Y, Jiang S, Gall PA, et al. Robust ACE2 protein expression localizes to the motile cilia of the respiratory tract epithelia and is not increased by ACE inhibitors or angiotensin receptor blockers. medRxiv 2020;https://www.medrxiv.org/content/early/2020/05/12/2020.05.08.20092866.
54. Sungnak W, Huang N, Bécavin C, Berg M, Queen R, Litvinukova M, et al. SARS-CoV-2 entry factors are highly expressed in nasal epithelial cells together with innate immune genes. Nat Med 2020 05;26(5):681–687.
55. Puelles VG, Lütgehetmann M, Lindenmeyer MT, Sperhake JP, Wong MN, Allweiss L, et al. Multiorgan and Renal Tropism of SARS-CoV-2. N Engl J Med 2020 May;.
56. Huang C, Wang Y, Li X, Ren L, Zhao J, Hu Y, et al. Clinical features of patients infected with 2019 novel coronavirus in Wuhan, China. Lancet 2020 02;395(10223):497–506.
57. Wei J, Alfajaro MM, Hanna RE, DeWeirdt PC, Strine MS, Lu-Culligan WJ, et al. Genome-wide CRISPR screen reveals host genes that regulate SARS-CoV-2 infection. bioRxiv 2020;https://www.biorxiv.org/content/early/2020/06/17/2020.06.16.155101.
58. Daniloski Z, Jordan TX, Wessels HH, Hoagland DA, Kasela S, Legut M, et al. Identification of required host factors for SARS-CoV-2 infection in human cells. Cell 2020;.
59. Ribeiro MT, Singh S, Guestrin C. "Why should i trust you?" Explaining the predictions of any classifier. In: Proceedings of the 22nd ACM SIGKDD International Conference On Knowledge Discovery and Data Mining; 2016. p. 1135–1144.
60. Pearl J. Causality. Cambridge university press; 2009.
61. Mostafavi S, Ray D, Warde-Farley D, Grouios C, Morris Q. GeneMANIA: a real-time multiple association network integration algorithm for predicting gene function. Genome Biology 2008;9 Suppl 1:S4.
62. Vanunu O, Magger O, Ruppin E, Shlomi T, Sharan R. Associating genes and protein complexes with disease via network propagation. PLoS computational biology 2010 1;6(1):e1000641+. http://dx.doi.org/10.1371/journal.pcbi.1000641.
63. Komurov K, White MA, Ram PT. Use of data-biased random walks on graphs for the retrieval of context-specific networks from genomic data. PLoS Comput Biol 2010;6(8).
64. Jiang B, Kloster K, Gleich DF, Gribskov M. AptRank: an adaptive PageRank model for protein function prediction on bi-relational graphs. Bioinformatics 2017 6;33(12):1829–1836. http://dx.doi.org/10.1093/bioinformatics/btx029.
65. Hristov BH, Chazelle B, Singh M. uKIN Combines New and Prior Information with Guided Network Propagation to Accurately Identify Disease Genes. Cell Syst 2020 06;10(6):470–479.
66. Letovsky S, Kasif S. Predicting protein function from protein/protein interaction data: a probabilistic approach. Bioinformatics (Oxford, England) 2003 7;19 Suppl 1(suppl 1):i197–i204. http://dx.doi.org/10.1093/bioinformatics/btg1026.
67. Deng M, Tu Z, Sun F, Chen T. Mapping Gene Ontology to proteins based on protein-protein interaction data. Bioinformatics 2004;20(6):895–902.
68. Nabieva E, Jim K, Agarwal A, Chazelle B, Singh M. Whole-proteome prediction of protein function via graph-theoretic analysis of interaction maps. Bioinformatics 2005 June;21 Suppl 1:i302–i310.
69. Goranci G, Henzinger M, Thorup M. Incremental exact min-cut in polylogarithmic amortized update time. ACM Transactions on Algorithms (TALG) 2018;14(2):1–21.
70. Kshirsagar M, Tasnina N, Ward MD, Law JN, Murali TM, Lavista Ferres JM, et al. Protein sequence models for prediction and comparative analysis of the SARS-CoV-2 -human interactome. Pac Symp Biocomput 2021;26:154–165.

Manuscript

Click here to access/download;Manuscript;sars-cov-2-networks.pdf

OXFORD (GIGA)^n SCIENCE

GigaScience, 20xx, 1–12

doi: xx.xxxx/xxxx
Manuscript in Preparation

Interpretable Network Propagation with Application to Expanding the Repertoire of Human Proteins that Interact with SARS-CoV-2

Jeffrey N. Law[1,†], Kyle Akers[1], Nure Tasnina[2], Catherine M. Della Santina[3], Shay Deutsch[4], Meghana Kshirsagar[5], Judith Klein-Seetharaman[6], Mark Crovella[7], Padmavathy Rajagopalan[8], Simon Kasif[3] and T. M. Murali[2,*]

[1]Interdisciplinary Ph.D. Program in Genetics, Bioinformatics, and Computational Biology, Blacksburg, VA, USA and [2]Department of Computer Science, Virginia Tech, Blacksburg, VA, USA and [3]Department of Biomedical Engineering, Boston University, Boston, MA, USA and [4]Department of Mathematics, University of California, Los Angeles, CA, USA and [5]AI for Good Lab, Microsoft, Redmond, WA, USA and [6]Department of Chemistry, Colorado School of Mines, Golden, CO USA and [7]Department of Computer Science, Boston University, Boston, MA, USA and [8]Department of Chemical Engineering, Virginia Tech, Blacksburg, VA, USA

[*] Corresponding author: murali@cs.vt.edu [†] Current Address: National Renewable Energy Laboratory, Golden, CO, USA

Abstract

Background: Network propagation has been widely used for nearly 20 years to predict gene functions and phenotypes. Despite the popularity of this approach, little attention has been paid to the question of provenance tracing in this context, e.g., determining how much any experimental observation in the input contributes to the score of every prediction. **Results:** We design a network propagation framework with two novel components and apply it to predict human proteins that directly or indirectly interact with SARS-CoV-2 proteins. First, we trace the provenance of each prediction to its experimentally validated sources, which in our case are human proteins experimentally determined to interact with viral proteins. Second, we design a technique that helps to reduce the manual adjustment of parameters by users. We find that for every top-ranking prediction, the highest contribution to its score arises from a direct neighbor in a human protein-protein interaction network. We further analyze these results to develop functional insights on SARS-CoV-2 that expand on known biology such as the connection between endoplasmic reticulum stress, HSPA5, and anti-clotting agents. **Conclusions:** We examine how our provenance tracing method can be generalized to a broad class of network-based algorithms. We provide a useful resource for the SARS-CoV-2 community that implicates many previously undocumented proteins with putative functional relationships to viral infection. This resource includes potential drugs that can be opportunistically repositioned to target these proteins. We also discuss how our overall framework can be extended to other, newly-emerging viruses.

Key words: network propagation; computational prediction; interpretable machine learning; provenance tracing; SARS-CoV-2; COVID-19; virus-host protein interaction networks;

Compiled on: September 21, 2021.
Draft manuscript prepared by the author.

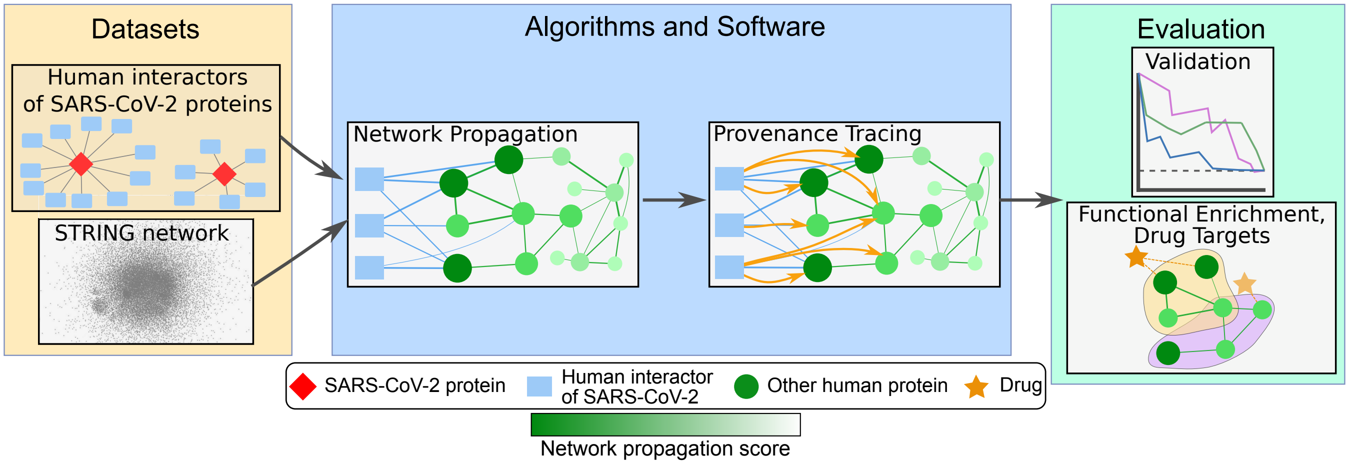

Figure 1. Overview of methodology. Algorithms and software for network propagation and provenance analysis take as input experimentally determined host-pathogen protein interactions and a human protein interaction network. Evaluation includes cross-validation, functional enrichment, and literature-based examination of promising protein targets and drugs.

Background

Network propagation algorithms have been widely used for nearly 20 years for function and phenotype prediction in systems biology [1, 2, 3, 4, 5, 6, 7]. More recently, applications of these techniques have included determination of genes associated with cancers and complex diseases [8] and denoising single-cell gene expression data [9]. Nowadays, network-based algorithms facilitate large-scale and automated data analysis of such complexity that it can be difficult for humans to understand the rationale that underlies a prediction, leading to decreased transparency and interpretability.

In this work, we consider the fundamental problem of tracing the provenance of a prediction back to the experimental sources [10]. Given a protein interaction network and a set of "sources", e.g., the human proteins that physically interact with SARS-CoV-2 [11], suppose we apply a network-based algorithm to score and prioritize additional proteins that may directly or indirectly interact with the virus. Can we determine which source proteins make the highest contribution to the score computed for each prediction? Surprisingly, this question has been insufficiently studied in the field of network biology [10]. This aspect takes particular importance in the context of COVID-19 or other clinically or scientifically critical applications, where it may be important to understand the rationale behind the computational prediction of a new drug target before committing to expensive experimental validation.

We present a simple and direct method to solve this problem for a large class of network propagation algorithms. Specifically, for each protein u in the network, we compute the precise contribution of each source to the score of u. This calculation enables us to sort the sources by their relative contributions to u and to quantify the relative roles of sources at different distances from u.

To evaluate the effectiveness of this strategy, we apply it to prioritize host proteins that may "functionally" (directly or indirectly) interact with SARS-CoV-2 proteins and host cellular processes that may be hijacked by the virus (Figure 1). To this end, we take advantage of a recently published dataset of human proteins that physically interact with SARS-CoV-2 [11]. Although these SARS-CoV-2 interactors are entry points to host cellular processes that may be hijacked by viral infection, the proteomics pipeline used to discover them [11] may not capture *in vivo* conditions and tissue-specific interactions, leading to false negatives. Therefore, we apply network propagation algorithms to these known human protein interactors of SARS-CoV-2 proteins (sources) and a whole-genome human protein interaction network from the STRING database [12]. We identify statistically-enriched host biological processes and pathways that include highly-ranking proteins computed by our methods. We illustrate how our provenance analysis can simplify visualizations of these processes and assist in understanding how they may be impacted by SARS-CoV-2.

Data Description

Here, we detail the different viral-human and human protein and functional interaction networks that we used in our study.

SARS-CoV-2–Human Protein-Protein Interactions (PPIs). We obtained 332 human proteins that interact with SARS-CoV-2 [11] and treated them as positive examples for our analysis. We added the ACE2 receptor to this set.

Functional and protein interaction networks. We used the human functional interaction network in the STRING database (version 11) [12], comprising of 18,886 nodes and 977,789 edges after applying a "medium" score cutoff of 400 and mapping to UniProt IDs. We used the interaction reliabilities provided by STRING as edge weights; we divided each value in STRING by 1,000 to scale them between 0 and 1. An edge in this network may be derived from experimental data or computational analysis. Thus, an edge may represent either direct physical binding or indirect functional interaction. Of the 332 viral interactors, 328 were present in this network; REEP6 (Q96HR9), PPIL3 (Q9H2H8), RAB18 (Q9NP72), and FKBP7 (Q9Y680) were missing.

We also computed results for PPI networks from two other sources: the BioGRID database [13], and the high-quality "HI-union" network published by Luck *et al.* [14]. For BioGRID, we considered two versions: (i) all PPIs (including protein complex membership), and (ii) only direct PPIs from yeast two-hybrid (Y2H) screens. For each of these networks, we did not use edge weights and restricted the nodes and edges to those in the largest

Network	# Nodes	# Edges	Edge Weights	Density	# SARS-CoV-2 inter. (/ 333)	# Nbrs. of sources
STRING (400)	18,886	977,789	Y	5.5×10^{-3}	329	12,480
BioGRID	16,595	488,787	N	3.6×10^{-3}	333	9,178
BioGRID-Y2H	12,582	87,801	N	1.1×10^{-3}	271	2,891
HI-union	9,053	64,193	N	1.6×10^{-3}	168	2,031

Table 1. Network statistics. For STRING, the weight cutoff applied is in parentheses. The column titled "# SARS-CoV-2 inter. (/ 333)" shows the number of sources that were in the network. The "# Nbrs. of sources" column shows the number of neighbors of the human proteins that interact with SARS-CoV-2 proteins (i.e., sources) in the given network.

connected component. See Table 1 for statistics of the network size and density.

Drug-protein interactions. We downloaded interactions among drugs and proteins from the DrugBank database (version 5.1.6) [15]. This dataset contained 16,503 drug-protein target pairs among 5,665 drugs and 2,891 target proteins. Limiting the targets to those in the STRING network reduced the number of drugs and targets to 5,589 and 2,769, respectively.

SARS-CoV-2–human A549 AP-MS interactome. We obtained 882 human proteins determined to interact with SARS-CoV-2 proteins by affinity purification followed by mass spectrometry analysis (AP-MS) [16]. This dataset was generated in A549 lung carcinoma cells transduced with lentivirus vectors expressing HA-tagged SARS-CoV-2 proteins. The authors used affinity purification with anti-HA antibodies to isolate stable complexes of human proteins bound to SARS-CoV-2 proteins. Subsequently, they identified and quantified the purified proteins by mass spectrometry.

SARS-CoV-2–human HEK293 AP-MS interactome. We obtained a set of 225 human proteins determined to interact with SARS-CoV-2 by AP-MS [17]. This dataset was generated by analyzing HEK293 embryonic kidney cells transfected with plasmid vectors expressing FLAG-tagged SARS-CoV-2 proteins. Affinity purification with anti-FLAG antibodies was used to isolate stable complexes of human proteins bound to SARS-CoV-2 proteins, and the purified proteins were identified and quantified by mass spectrometry.

SARS-CoV-2–human BioID interactome. We obtained a set of 2,241 human proteins determined to interact transiently or weakly with SARS-CoV-2 proteins by using proximity-dependent biotinylation (BioID) [18]. This dataset was generated by analyzing A549 lung carcinoma cells transduced with lentivirus vectors expressing SARS-CoV-2 proteins fused with a bacterial biotin ligase. The addition of biotin resulted in the biotinylation of host proteins in the proximity of SARS-CoV-2 proteins. Biotinylated proteins were purified and then identified and quantified by mass spectrometry. Compared to interactomes identified by AP-MS, BioID is more capable of identifying weaker interactions in poorly soluble intracellular locations such as membranes and organelles.

Differential protein abundance in SARS-CoV-2–infected iAT2 cells. We obtained a set of 5,665 human proteins determined to have differential abundance in response to SARS-CoV-2 infection [19]. This dataset was generated by infecting induced pluripotent stem cell-derived alveolar epithelial type 2 cells (iAT2) with SARS-CoV-2 and measuring protein abundance by quantitative mass spectrometry at 1, 3, 6, and 24 hours post-infection. The authors compared protein abundance in infected iAT2 cells with that of the uninfected iAT2 controls to obtain differentially-expressed proteins. In our analysis, we used the set of proteins with differential expression (FDR p-value < 0.05) at any of the 1, 3, 6, and 24 hours post-infection.

Differential gene expression in upper airway samples in SARS-CoV-2–infected patients. We obtained three sets of human proteins determined to have differential gene expression in cells infected with respiratory viruses [20]. To generate this dataset, the authors used metagenomic RNA-seq to identify and quantify both human and viral RNA expression in upper airway samples collected from patients with acute respiratory illness. They compared the gene expression values between samples that contained SARS-CoV-2 to uninfected samples in order to obtain differentially-expressed genes. They also identified additional viral infections including SARS-CoV, HRV, Influenza, HMPV, RSV, PIV in patient samples. Comparing SARS-CoV-2 infections with other viral infections and other viral infections with uninfected samples yielded two additional sets of differentially-expressed genes. In our analysis, we used the genes with differential expression (FDR p-value < 0.05) in these three sets obtaining (i) 1,383 genes from SARS-CoV-2-infected cells compared with uninfected samples, (ii) 7,338 genes from SARS-CoV-2-infected cells compared with other viral infections, and (iii) 5,779 genes from other viral infections compared with uninfected samples.

From each of these interactome and differential expression datasets, we removed human proteins used as positive examples in our analysis and the proteins that were not present in the STRING network. This step resulted in 2,080, 807, 212 proteins, respectively, from the interactome datasets and 5,447, 1,293, 6,940, and 5,472 proteins, respectively, from the differential expression datasets. We used Fisher's exact test to estimate the statistical significance of the overlap between the remaining proteins and our top-ranking proteins.

Analyses

Various network propagation methods have been successfully used in diverse applications in systems biology [21]. In particular, we model network propagation using the Regularized Laplacian (RL) [22]. As we describe below ("Methods"), RL has the benefit of two mutually-reinforcing interpretations. On one hand, it can be understood as an optimal labeling of network nodes, when some node labels are known *a priori*. On the other hand, it can be seen as the result of diffusion, i.e., a continuous-time random walk, on the network. Under this second interpretation, we derived a novel mathematical formula for the expected length of the path traversed in the network by the random walker, which we then used to characterize our top-ranking proteins.

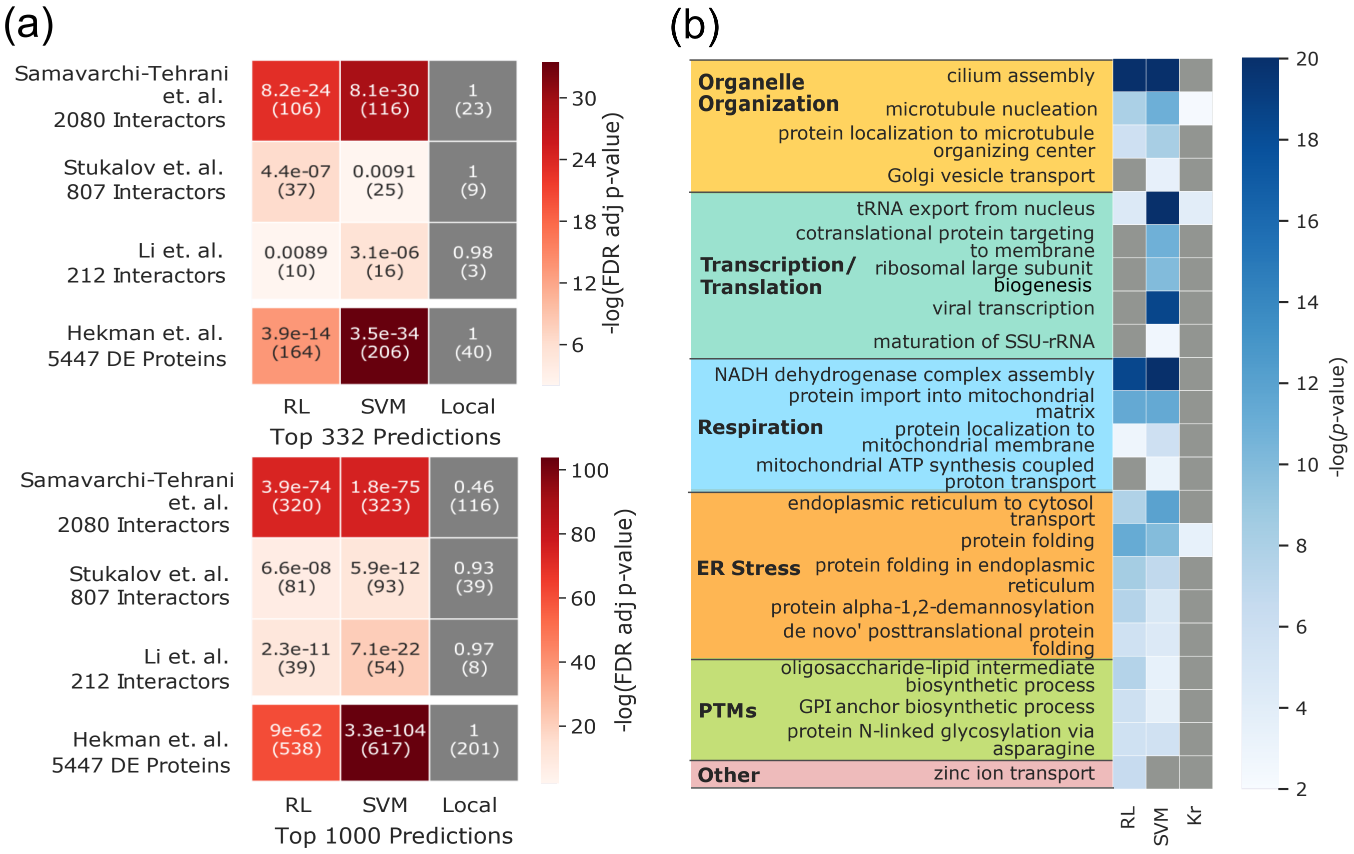

Figure 2. Network propagation results. (a) Heatmap showing the FDR adjusted *p*-value from the hypergeometric test for the overlap between the top-ranking predictions of RL, SVM, and Local and three new experimental datasets of SARS-CoV-2-human protein interactions [18, 17, 16] and one dataset of differentially expressed (DE) proteins after SARS-CoV-2 infection [19]. Each cell displays the FDR-adjusted *p*-value and the number of overlapping proteins in parentheses. A gray cell indicates a *p*-value larger than 0.01. (b) Heatmap summarizing GO biological process terms enriched in top ranking proteins from RL and SVM and human interactors of SARS-CoV-2 proteins (indicated as 'Kr'). We manually grouped the terms into broader categories shown in bold text. A gray cell indicates a *p*-value larger than 0.01. We examine the relevance of these biological processes to SARS-CoV-2 and COVID-19 in "Enriched Biological Processes" in the supplementary results and in "Discussion".

Prioritization of Potential SARS-CoV-2 Interactors

Our underlying hypothesis was that network propagation via methods such as the RL yields a reasonable mechanism for predicting SARS-CoV-2 interactors. Therefore, we applied RL to the set of positive examples to rank the remaining proteins in the STRING network. We also ranked these proteins using multiple other network propagation methods and off-the-shelf classifiers [23, 24, 25, 26]. We used a stratified sampling approach to estimate the statistical significance of the resulting node scores (see "Statistical Significance of Node Scores" in the supplementary methods). The sampling accounted for the possibility that if many sources have high degree, then scores may tend to be large overall in the network. Henceforth, for every method, we only considered proteins in the network that had a *p*-value less than 0.05.

To decide which methods to select for subsequent analyses, we compared them using 5-fold cross validation ("Comparison of Cross-Validation Results" in the supplementary results and Figure S1). RL, random walk with restarts (RWR)[23], and deepNF [26] had the highest values of area under the precision-recall curve followed by SVM and logistic regression. RL achieved marginally worse values of area under the precision-recall curve than RWR and deepNF. We selected one network propagation method (RL) and one supervised classifier (SVM) for the following reasons. We preferred RL over deepNF because the provenance tracing method we developed for RL enabled its results to be more easily interpreted than those for deepNF. Since RL and RWR produced highly similar predictions with a very high Spearman's correlation for the ranking of all proteins ("Overlap among algorithms" in the supplementary results and Figure S2), we selected RL as representative of the two methods. We chose SVM among the two off-the-shelf classifiers since it also had very good performance in cross-validation. We considered the top 332 predictions of RL and SVM that were statistically significant at $p < 0.05$ (File S1), which we refer to as "top-ranking proteins" below.

Three recent publications or preprints have independently discovered physical interactions between SARS-CoV-2 and human proteins [18, 16, 17]. These datasets differed in the type of host cell in which the viral proteins were expressed and the experimental methods used to determine if two proteins interacted. ("Datasets"). The top-ranking proteins for both RL and SVM had significant overlaps with each of the three new datasets, while the results for Local were not statistically significant (p-value > 0.01) (Figure 2(a)). We observed an especially striking overlap with the "proximity interactome" [18]. Approximately one-third of the 332 and 1000 top-ranking proteins computed by RL were present in this dataset of 2,080 interactions (p-value 8.2×10^{-24} and p-value 3.9×10^{-74} respectively).

The corresponding publication used BioID with the fast-acting miniTurbo enzyme [18], a technique that is useful for discovering viral-host protein interactions that take place at intracellular membranes and poorly soluble organelles, which are difficult to profile using classical biochemical purification approaches used in the other publications [11, 16, 17]. Thus, our top-ranking proteins may be members of biological processes that occur in such locations in the cell. These three independent datasets provide strong support of our predictions. Our top-ranking proteins that do not overlap with these resources may interact with viral proteins indirectly and thus would not be captured by assays that test for direct protein-protein interactions.

We additionally tested the overlap between our top predictions and independent experimental datasets identifying differential expression of proteins in response to SARS-CoV-2 infection [19]. As in the previous analysis, we observed that the results for Local were not statistically significant (p-value > 0.01), while both RL and SVM had significant overlaps with differential protein abundance in SARS-CoV-2 infected cells compared with uninfected cells [19](Figure 2(a)). Approximately half of the 332 and 1000 top-ranking proteins computed by RL were present in this dataset of 5,447 differentially expressed proteins (p-value 3.9×10^{-14} and p-value 9×10^{-62} respectively). This high overlap may indicate that these proteins are involved with changes in host protein expression occurring in SARS-CoV-2 infected cells, via either direct or indirect virus-host protein interactions.

In contrast, when we analyzed gene expression measurements in response to SARS-CoV-2 infection [20], we did not observe a significant overlap between our top-ranking proteins and differentially-expressed genes (Figure S8). This result may be attributed to a difference in cell types used for measuring gene expression data, including cells not directly infected by the virus. Moreover, the lack of edges connecting transcription factors to target genes in the PPI network we used may limit the size of the overlap between interactors predicted by RL and SVM with differentially-expressed genes.

We tested for enrichment of Gene Ontology (GO) biological processes (Benjamini-Hochberg corrected p-value ≤ 0.01) among the top-ranking proteins from RL and from SVM, as well as in the interactors of SARS-CoV-2 ("Functional Enrichment" in the supplementary methods). Our top-ranking proteins were enriched in five broad categories of GO biological processes: organelle organization, transcription and translation, respiration, ER stress, and post-translational modifications (Figure 2(b), Figure S5 , and File S2). We examine the relevance of these processes to the viral life cycle in more detail in "Discussion" and in "Enriched Biological Processes" in the supplementary results.

Tracing the Provenance of Top-Ranking Proteins

We can interpret the RL in terms of a continuous-time random walk over the network, which is governed by the internal parameter α. We are interested in the node reached by the walker after a random time that depends on α. The expected number of transitions made by the walker increases with the parameter α ("Analytical Perspective on the RL and Expected Path Length" in the supplementary methods). Hence for larger values of α, the "influence" of the sources is diffused more broadly across the network. To test how this spreading of "influence" affects our results, we varied α over four orders of magnitude from 0.01 to 100 and performed two analyses. First and most importantly, for each top-ranking protein computed by the RL, we developed a systematic procedure to determine the provenance of its score, i.e., which SARS-CoV-2 interactors made the greatest contributions to this score. For our second analysis, we developed a new methodology to select a value of α. We were motivated to do so since we could not use the common practice of choosing the parameter's value based on maximization of cross-validation performance: the AUROC, AUPRC, and precision at 0.3 recall of the RL varied very little with α (Figure S3).

For provenance tracing, we took advantage of the fact that the score computed by the RL for each protein in the network is a linear combination of contributions from source proteins ("Methods"). Therefore, for each protein u in the network, we sorted the source proteins by their relative contributions to the score of u (File S3). Figure 3(a)–(d) provide illustrative examples of the practical usefulness of provenance tracing. We used a value of $\alpha = 3.4$ to obtain these results. We present our method for selecting α at the end of this section.

In Figure 3(a), we display the top 332 ranking proteins computed by the RL that are annotated to the enriched GO term "protein folding in endoplasmic reticulum". For each such protein, we also show all the sources that interact with it as well as the viral proteins that in turn interact with the sources. This network is complex and difficult to understand. In contrast, in Figure 3(b), we connect each top-ranking protein only to the two source proteins that contribute the most to its score. This simplified network considerably facilitates the interpretation and rationalization of the RL's predictions. Figure 3(c,d) are similar in nature and correspond to the enriched term "cilium assembly". We return to the biological insights present in these networks in "Discussion".

Next, we considered the effect of α on the amount of diffusion in the network. When α was very small, e.g., 0.01, we expected the highest contributing sources to be direct neighbors of top-ranking proteins. As α increased, and the random walker traversed longer paths in the network, we expected more of the highest contributors to not be directly connected by an edge to top-ranking proteins. Contrary to our expectations, we found that for every value of α and for every top-ranking protein u (till a rank of 1,000), the source protein with the highest contribution to u's score was always a neighbor of u. Even when we considered the second and third highest contributors, we found that they were more than one edge away for as few as 2% of the top-ranking proteins for $\alpha = 0.01$. This number increased only to 25% for $\alpha = 100$.

The STRING network includes both direct, physical and indirect, functional PPIs. Therefore, we sought to see if this trend in the provenance analysis held for networks with only physical interactions corresponding to direct binding and indirect protein complex membership. We repeated the analyses up to this point on three other PPI networks: BioGRID, BioGRID-Y2H, and HI-union ("Methods"). For BioGRID, the results were comparable to those for STRING. The highest contributor was always a neighbor, except for $\alpha \geq 10$ where up to 3% of nodes received most of their score from a source more than one edge away. The second and third highest contributor was more than one step away for as few as 8% of top-ranking nodes for $\alpha = 0.01$, and up to 41% for $\alpha = 100$. For BioGRID-Y2H and HI-union, which are smaller, sparser networks with only direct PPIs, only 300–400 nodes had scores that were statistically significant at the 0.05 level. The highest contributing source was more than one step away for as many as 10–30% of the top-ranking nodes, even for $\alpha = 10$. For the second highest contributor, this percentage jumped to more than 50% for $\alpha = 0.01$ itself.

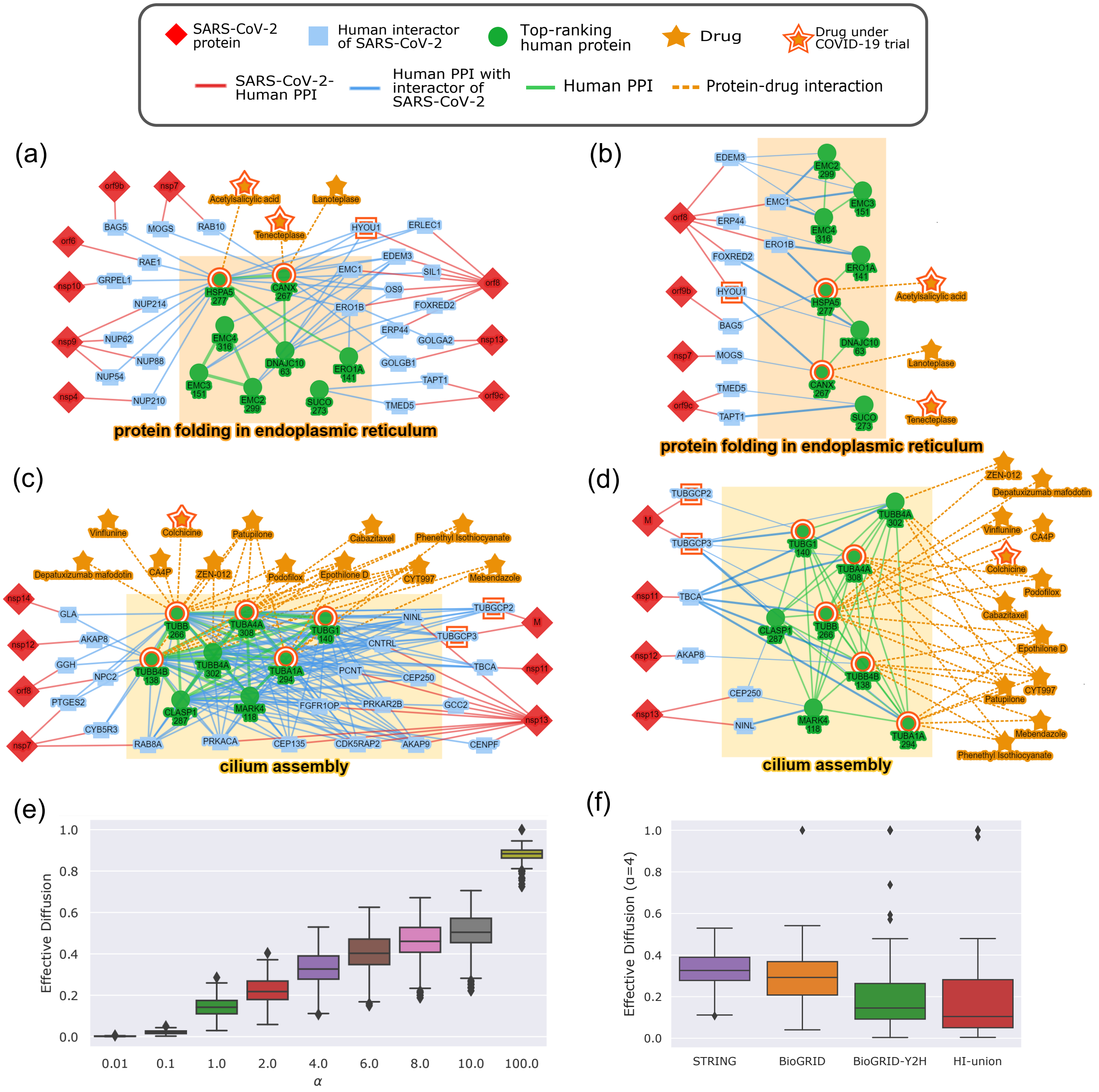

Figure 3. Provenance tracing results and illustrative examples of networks. (a) Network of the top 332 ranking proteins for RL (green nodes) that are annotated to the enriched term "protein folding in ER". For each top-ranking protein, we display its connections with all neighboring SARS-CoV-2 interactors. (b) The same network as in (a) except that we display only the top-two contributing SARS-CoV-2 interactors for each top-ranking protein. (c) Network of the top 332 ranking proteins for RL (green nodes) that are annotated to the enriched term "cilium assembly." (d) The same network as in (c) except that we display only the top-two contributing SARS-CoV-2 interactors for each top-ranking protein. In all four network visualizations, the number below the name of a green protein is its rank as computed by the RL. Proteins discussed in the text are highlighted with a red border. In (a,c), we removed STRING edges with weight < 700 to simplify the visualization. In (c,d), we removed drugs that promote clotting. (e) Distribution of effective diffusion for the top 332 ranking proteins for different values of α. (f) The same distribution as (e) except comparing different networks with $\alpha = 4.0$.

To further characterize the contribution of non-neighboring sources, we defined the *effective diffusion* to a protein u as the fraction of its score $s(u)$ that arose from the non-direct neighbors of u that were also SARS-CoV-2 interactors. As expected, the effective diffusion to the top-ranking proteins increased with α with values close to zero for $\alpha = 0.01$ and a median of 0.88 for $\alpha = 100$ (Figure 3(e)). We concluded that the neighbours of the sources received non-trivial contributions to their RL scores from indirectly-connected sources only for values of $\alpha = 1$ and higher.

We repeated these experiments for BioGRID, BioGRID-Y2H, and HI-union (see Figure 3(f) and Figure S9). BioGRID maintained fairly similar results to STRING. On the other hand, for the other two networks, their effective diffusion values were quite a bit smaller (difference from STRING about 0.2 on average). Taken together, these results suggest that in the sparser networks (BioGRID-Y2H and HI-union), a top-ranking protein has fewer sources as direct neighbours than in the denser networks (STRING and BioGRID) but a larger proportion of its score arises from these adjacent sources.

These results motivated us to test a different method for selecting an appropriate value of α for downstream analysis. As mentioned earlier, we mathematically derived a new expression for the expected value of the path length of the random walker ("Analytical Perspective on the RL and Expected Path Length" in the supplementary methods). To our knowledge, no such formula is known for the interpretation of the RL as a continuous-time Markov chain. This value depended on α, the topology of the network, and which proteins interacted with SARS-CoV-2. We computed the expected path length for different values of α (Table S1). Independently, we computed the distribution of path lengths in the network from SARS-CoV-2 interactors to every other protein (Figure S10). The median number of edges in these paths was three. Therefore, we set the value of $\alpha = 3.4$ for which the expected path length of the random walker was 3.04 (Table S1). The median effective diffusion for this value of α was around 0.3. We used this value of α to generate the results presented in this work.

Discussion

The COVID-19 pandemic and its medical and economic impact have created an urgent challenge for biomedical researchers to understand infection mechanisms used by SARS-CoV-2 and to develop therapeutics against the disease [27]. A manifestation of this community response is the first protein-protein interactome associated with the SARS-CoV-2-human interface [11]. This set of human proteins reported to interact with SARS-CoV-2 is likely to have both false positives and false negatives due to the properties of the proteomic screening pipeline used.

In this work, we sought to further extend the results of this study to significantly expand the resources available to the COVID-19 community by producing an extended set of putative SARS-CoV-2 interactors. Comparison of our results with independently-generated SARS-CoV-2-human protein interaction networks [18, 17, 16] provides substantial experimental support for our predictions. We note that complementary efforts are based on protein structures [28], observational studies of treatments being administered to patients [29], shortest paths in protein networks [30], propagation in protein networks with predicted SARS-CoV-2 interactors [31], and exploratory analyses of virus-host-drug networks [32].

A notable new feature of our methodology is tracing the provenance of each of our predictions back to the most informative experimental sources [10]. In principle, the RL computes scores by integrating over all paths in the network. We were surprised to see that the top-contributing sources were invariably direct neighbours of the top-ranking predictions in the STRING network. A partial explanation for this trend may be the fact that as many as 5,331 proteins in the STRING network were direct neighbors of at least one source protein, even when we considered only interactions with weight at least 0.9 (the STRING database deems edges with such weights to be of "very high quality"). Thus, the structure of the STRING network and central location of sources within it may cause the RL both to give high ranks only to direct neighbors of sources and to channel propagation primarily along these direct connections. We stress that using only the interactions between sources and their neighbors in the network does not result in high-quality predictions, as evidenced by the relatively poor cross-validation performance of the Local algorithm. Thus, the integration of multiple paths by the RL plays a key role in prioritizing which neighbors of the sources are more likely to be potential interactors of SARS-CoV-2 proteins than others.

COVID-19 research has focused disproportionately on a small set of human proteins [33]. Our research has the potential to expand the repertoire of host proteins that are studied in the context of COVID-19 and thereby open new directions of study of the disease. The cellular processes in which our top-ranking proteins participate suggest how the virus may infect human cells. We discuss two illustrative examples of the type of insights provided by our approach, highlighting several proteins targeted by drugs that are already in clinical trials for COVID-19. We remind the reader that we computed functions enriched in the top-ranking proteins, performed the provenance analysis independently, and then integrated the results in the protein networks we visualized.

The Role of Endoplasmic Reticulum Stress, HSPA5, and Anti-Clotting Drugs

Our analysis points to a connection among interactors of SARS-CoV-2, proteins involved in endoplasmic reticulum (ER) stress, and anti-clotting drugs ((Figure 3(a,b))). The GO biological process "protein folding in endoplasmic reticulum" was enriched in the top-ranking proteins (p-value 4.32×10^{-9} for RL and 0.28 for interactors of SARS-CoV-2). HSPA5, also referred to as glucose regulated protein (GRP78) or immunoglobulin binding protein (BiP) in the literature, is evolutionarily conserved from prokaryotes to humans [34]. It has a repertoire of functions associated with ER stress response. HSPA5 is usually localized in the ER. When the ER is stressed, HSPA5 can translocate to the cell surface, the nucleus and mitochondria [35, 36]. On the cell surface, HSPA5 plays a multi-functional role in cell proliferation, cell viability, apoptosis, and regulation of innate and adaptive immunity [36, 37].

HSPA5 has been proposed as a universal target for human diseases [38]. It has increasingly well-documented essential interactions and activities during viral infections. In particular, the role of HSPA5 in viral entry and pathogenesis has been widely investigated. SARS-CoV infection has been shown to lead to ER stress and the up-regulation of HSPA5 [39, 40]. The S protein of SARS-CoV can induce transcriptional activation of HSPA5 [40]. This protein can serve as a point of attachment for both MERS-CoV and bat coronavirus (bCoV HKU9) [41]. Both Zika virus and Japanese encephalitis virus use HSPA5 to prevent apoptosis and to help in viral replication [42]. A recent molecular docking study has predicted HSPA5 as a potential receptor for the SARS-CoV S

protein [43]. The observed expression *in vitro* of HSPA5 in airway epithelial cells suggests that it may serve as an additional receptor for SARS-CoV-2 in these cells [44]. Based on our network-based analysis and support in the literature, we hypothesize that HSPA5 may serve as a co-receptor, a point of viral attachment, or aid in viral entry of SARS-CoV-2.

Blood hypercoagulability is reported to be common among COVID-19 patients [45]. Top-ranking proteins HSPA5 and CANX act as chaperones for pro-coagulant proteins such as Factor V and Factor VIII. Once Factor VIII is secreted, it binds to another pro-coagulant protein von Willebrand factor (vWF) to prevent degradation of clots [46]. Although Factor V, Factor VIII, and vWF are not among the top-ranking proteins and thus do not appear in Figure 3(a,b), this network is suggestive of mechanisms that SARS-CoV-2 may use to cause abnormal blood coagulation.

Anti-coagulant drugs that interact with HSPA5 or CANX include Tenecteplase, a third generation plasminogen activating enzyme and the investigational drug Lanoteplase, which is a serine protease that binds to fibrin leading to the formation of plasmin [47], an enzyme that breaks clots. Lanoteplase is a second-generation derivative of Alteplase, and a third generation derivative of recombinant plasminogen. It is notable that there are clinical trials for Tenecteplase (ClinicalTrials.gov, NCT04558125, NCT04505592) and Alteplase (ClinicalTrials.gov, NCT04357730, NCT04640194) to test their effectiveness in treating COVID-19. Aspirin, also present in (Figure 3(a,b)), binds to and inhibits the ATPase activity of HSPA5 [48]. Aspirin is currently involved in 16 clinical trials (ClinicalTrials.gov), with one testing the effects of aspirin at various levels of COVID-19 severity (NCT04365309), and another testing whether early treatment of COVID-19 patients with aspirin and vitamin D can inhibit the production of blood clots and decrease rates of hospitalization (NCT04363840).

Cilium Assembly and Tubulin-Modulating Drugs

GO biological processes related to cilia were significantly enriched in the top-ranking RL and SVM predictions. An example is "cilium assembly" (p-value 6.84×10^{-26} for RL vs. 0.31 in the human interactors of SARS-CoV-2. Many proteins annotated to this term belong to the tubulin family, which are components of microtubules. The SARS-CoV-2 M protein binds to two γ-tubulins (TUBGCP2 and TUBGCP3), which interact with several α- and β-tubulins among the top 332 predictions (Figure 3(c,d)). Microtubules are polymers that provide shape and structure to eukaryotic cells and are necessary in cell transport and cell division, among other functions [49]. α- and β-tubulins compose microtubule filaments, while γ-tubulins connect them to the microtubule organizing center.

Viruses commonly utilize microtubules for cellular entry, intra-cellular trafficking, and exit from cells [50]. For instance, the S protein of human α-coronavirus interacts with tubulin α and β chains [51], suggesting that tubulin may be involved in the transport and localization of the S protein and its assembly into virions [51]. Relevant to SARS-CoV-2, microtubules are the primary structural component of cilia, which line epithelial cells in the respiratory tract and are responsible for the transport of mucus out of cells [52]. The ACE2 receptor that SARS-CoV-2 uses to enter cells appears to be expressed primarily on the cilia of respiratory tract epithelial cells [53, 54], further implicating microtubules in viral infection. The combination of high expression levels of ACE2 and the presence of cilia may also explain the detection of the virus in multiple organs [55] and the deleterious effect of COVID-19 on the renal, gastroinstestinal, and olfactory systems [56]. The drugs that target Tubulin proteins (Figure 3(c,d)) are mostly anti-mitotic agents, which are being investigated as anti-cancer therapeutics. It is notable that 26 ongoing clinical trials (ClinicalTrials.gov) are testing the effectiveness of Colchicine against COVID-19.

Our work also sets the stage for follow-up analyses on SARS-CoV-2. Integrating new datasets of SARS-CoV-2-human protein interactions [18, 17, 16] and human proteins whose deletion inhibits viral replication [57, 58] with other omics data using our methods and with orthogonal analysis techniques promises to predict more biologically meaningful networks and processes impacted by the virus. In particular, single-cell RNA-seq data offer many opportunities to examine cellular heterogeneity and context-specific interactions.

Potential Implications

The approach we advocate here is inspired by the general framework of producing explanations for machine learning methods [59]. This area of "explanations" of predictions is receiving strong interest because of deep learning. While the idea has previously been studied in graphical models [60], most machine learning methods are not fully interpretable by the fairly strict definition of Kasif and Roberts [10]: tracing each prediction to the experimental evidence that supports it. This notion of explanation is a special but particularly important case for computational genomics and systems biology.

Causal perturbations [60] provide a general approach for producing explanations of this type for virtually any predictive model. Consider a model with experimental evidence that a gene g performs a function f. We perturb the variable associated with the gene, e.g. we change the probability Pr(g performs f) = 1 to Pr(g performs f) = 0. We then compute the change in probability of every other variable in the model due to this perturbation in order to assess the importance of this particular gene-function pair.

For network propagation, this idea yields the special case discussed in this work that is amenable to very efficient computation. Our strategy for tracing provenance extends to any algorithm that makes predictions using a linear combination of evidence such as logistic regression and GeneMania [61]. In particular, it is applicable to the large number of random-walk-based methods that have been developed for predicting disease genes or annotations to GO terms [62, 63, 64, 65].

An important future line of research will be to develop provenance tracing techniques for other classes of network-based methods such as Markov random fields (MRFs) [66, 67] and mincut based methods [6, 68]. For MRFs, we can apply the general perturbation-based method described above. For mincut-based methods, it is possible to recalculate the cut for any single change in experimental data using dynamic data structures [69]. Thus, the provenance tracing approach that we advocate here has many natural follow-ups that we expect to be studied by the community in the future.

It remains to be seen whether the trends we observed on the contributions from direct neighbors generalize to these methods and to annotations of terms in the Gene Ontology or the Human Phenotype Ontology terms. In general, it is quite likely that sources that are not direct neighbors may make substantial contributions to scores. In these cases, new algorithmic developments may be required to trace the paths by which the sources

spread their influence to a given node.

Our work provides significant new data and software resources to the COVID-19 community. Three properties of our results facilitate their use by experimentalists who are seeking to obtain new insights into the pathogenesis of this disease. First, the prioritized list of predicted interactors of SARS-CoV-2 (File S1) contains druggable targets that may be promising to study further. Second, our provenance analysis provides the rationale underlying each prediction by directly linking to the relevant experimental input. Third, the viral-human protein interaction networks corresponding to enriched GO terms (Figure 3 and Figure S6) are available for visualization and download on GraphSpace (http://graphspace.org/graphs/?query=tags:2021-sarscov2-network-analysis). Examination of these networks provides further context for the predictions.

We conclude by noting that our methodology is general purpose and easy to generalise to a new virus. The software requires a dataset of host proteins that interact with the virus and an interaction network among the host proteins themselves. The virus-host network may be determined experimentally [11]. If such a dataset is not available, a user can predict the network computationally from the sequence of the viral genes and interaction networks for phylogenetically similar viruses [70]. Subsequently, a user can apply network propagation to predict additional human proteins and biological processes that may be targeted by the virus.

Methods

Algorithms

To facilitate the complete reproducibility of our results, we now describe the RL algorithm that we use for label propagation and prediction. We present the other methods that we use (GeneMANIA, SinkSource, RWR, Local, deepNF, the Support Vector Machine, and Logistic Regression) and implementation details in "Other Algorithms" in the supplementary methods. We are given a weighted, undirected network $G = (V, E, w)$, where each node in V is a human protein, each edge (u, v) represents an interaction between proteins u and v, and $w : E \rightarrow (0, 1]$ is a function specifying the weight of each edge in E. Informally, the weight of an edge indicates our confidence in the experimental data supporting the corresponding protein-protein interaction. We are also given a set $P \in V$ of positive examples consisting of the human proteins that interact with SARS-CoV-2 proteins [11]. Each node in G is a human protein and each edge represents a physical or functional interaction between two proteins. We seek to compute a score vector $\vec{s} \in \mathbb{R}^n$, where n is the number of nodes in G. For every node v, the score $s(v)$ in this vector indicates our confidence that node v either physically interacts with or is functionally linked to a SARS-CoV-2 protein.

Regularized Laplacian [22]. Given a parameter $\alpha > 0$, we compute $\vec{s}$ using the following steps:

i. Define a label vector $\vec{y}$ over the nodes in G where $y(u) = 1$ if node u is in P and $y(u) = 0$, otherwise.
ii. Define $W \in \mathbb{R}^{n\times n}$ as the adjacency matrix of G with edge weights, i.e., the entry in row u and column v of W equals w_{uv} if (u, v) is an edge in G and 0, otherwise.
iii. Define D as a diagonal matrix with $D_{uu} = \sum_v w_{uv}$, for every node u in G.
iv. Compute the $\mathbb{R}^{n\times n}$ matrix $\tilde{W} = D^{-1/2}WD^{-1/2}$, which denotes the normalized network.
v. Compute the Laplacian of G as $\tilde{L} = \tilde{D} - \tilde{W}$, where we define $\tilde{D}$ to be a diagonal matrix with $\tilde{D}_{uu} = \sum_v \tilde{w}_{uv}$.
vi. Compute the vector $\vec{s} = (I + \alpha\tilde{L})^{-1}\vec{y}$.

The RL was introduced by Zhou and Schölkopf. Since then, several variations of this method have been published. The version we use is identical to the strategy used by Fouss *et al.* [22]. We provide the intuition behind the resulting RL matrix (i.e., $(I + \alpha\tilde{L})^{-1}$) and discuss its properties in "Analytical Perspective on the RL and Expected Path Length" in the supplementary methods. In particular, we derive an expression for the expected path length of the continuous-time Markov chain corresponding to the RL. As far as we know, this mathematical analysis has not previously been published.

Tracing the Provenance of Prediction Scores

Let K denote the RL matrix $(I + \alpha\tilde{L})^{-1}$. We remind the reader that the RL algorithm ranks proteins based on diffusion scores that associate a node u in the network with a diffusion score $s(u)$, where $s(u) = \sum_{v\in P} K_{uv}$, where v ranges over the set P of all SARS-CoV-2 interactors. For every protein u, we sorted the proteins in P in decreasing order of the values of K_{uv}, where v ranged over P. In the manner, we ranked the experimentally determined interactors that in decreasing order of their contributions to each node's diffusion score. This analysis is important for tracing the provenance of computational predictions to their experimental sources [10].

Availability of Source Code and Requirements

- Project name: SARS-CoV-2-network-analysis
- Project home page: https://github.com/Murali-group/SARS-CoV-2-network-analysis
- Operating system(s): Platform independent (tested and applied on Linux and Mac OS)
- Programming language: Python
- Other requirements: Please see https://github.com/Murali-group/SARS-CoV-2-network-analysis/blob/master/requirements.txt
- License: GNU General Public License (GPL) v3

Availability of supporting data and materials

We used publicly available datasets for our analysis. We downloaded these data from the respective publications or websites. We also provide the following supplementary files.

File S1: The prediction rank and p-value computed by RL and SVM for each human protein on the STRING network, the list of drugs that target the protein (when this information is available in DrugBank), and the closest SARS-CoV-2 interactor and SARS-CoV-2 protein. For the last piece of information, we computed the shortest weighted path, where we defined the weight of a path to be the sum of the absolute value of the base-10 logarithm of the weights of the edges in the path.

File S2: Enrichment results for RL, SVM and the viral interactors on GO biological processes.

File S3: Provenance tracing matrix of contributions to the network propagation score from each SARS-CoV-2 interactor to every top-ranking protein.

Declarations

Abbreviations

AP-MS: affinity purification followed by mass spectrometry analysis; AUPRC: area under the precision-recall curve; AUROC: area under the receiver-operator characteristic curve; bCoV: bat coronavirus; COVID-19: novel coronavirus disease 2019; BioID: proximity-dependent biotinylation; BiP: immunoglobulin binding protein; ER: endoplasmic reticulum; GM: GeneMania; GPL: General Public License; GO: Gene Ontology; GRP: glucose regulated protein; HIV-1: human immunodeficiency virus 1; HSV-1: herpes simplex virus type 1; KEGG: Kyoto Encyclopedia of Genes and Genomes; LogReg: Logistic Regression; MERS: Middle East respiratory syndrome; NSF: National Science Foundation; PPMI: Positive Pointwise Mutual Information; RWR: random walk with restarts; RL: Regularized Laplacian; SARS: severe acute respiratory syndrome; SARS-CoV-2: severe acute respiratory syndrome coronavirus 2; SS: SinkSource; SVM: Support Vector Machine; vWF: von Willebrand factor; USDA-NIFA: United States Department of Agriculture National Institute of Food and Agriculture

Consent for Publication

Not applicable.

Competing Interests

The authors declare that they have no competing interests.

Funding

TMM acknowledges support from National Science Foundation (NSF) grants DBI-1759858 and MCB-1817736. KA acknowledges support from the Genetics, Bioinformatics, and Computational Biology program at Virginia Tech. JK acknowledges support from NSF grant CCF-2029543. MC acknowledges support from NSF grant CNS-1618207. CMDS acknowledges support from the Hariri Institute and the Department of Biomedical Engineering at Boston University. PR acknowledges support from NSF grant CBET-1510920 and USDA-NIFA grant 2018-07578. PR and TMM acknowledge support from the Computational Tissue Engineering Graduate Education Program at Virginia Tech.

Author Contributions

TMM and SK proposed the study. TMM, SK, MC, JL, SD, MK, and JK contributed computational ideas. JL was the primary author of the software and led the computational analysis, with significant inputs from KA, NT, and CMDS. All authors analyzed the results. TMM, MC, PR, and SK wrote the paper with contributions and revisions from all authors. All the authors read and approved the final manuscript.

Acknowledgments

The authors wish to thank S. Alabdullatif, S. Alshuaib, M. Iennaco, M. Kouzminov, S. Murthy, S. Makwana, N. Naguib, C. Tagliettii, and M. Zanna for exploratory research on this data and insightful and thought-provoking analysis. We also thank Roded Sharan, Noga Alon, Dan Lancour and Rich Roberts for discussions that helped formulate the techniques and ideas we used in this paper.

References

1. Vazquez A, Flammini A, Maritan A, Vespignani A. Global protein function prediction from protein-protein interaction networks. Nat Biotechnol 2003;21(6):697–700.
2. Letovsky S, Kasif S. Predicting protein function from protein/protein interaction data: a probabilistic approach. Bioinformatics 2003;19 Suppl 1:i197–204.
3. Karaoz U, Murali TM, Letovsky S, Zheng Y, Ding C, Cantor CR, et al. Whole-genome annotation by using evidence integration in functional-linkage networks. Proceedings of the National Academy of Sciences of the United States of America 2004 3;101(9):2888–2893. http://dx.doi.org/10.1073/pnas.0307326101.
4. Deng M, Chen T, Sun F. An integrated probabilistic model for functional prediction of proteins. J Comput Biol 2004;11(2-3):463–75.
5. Fraser AG, Marcotte EM. A probabilistic view of gene function. Nat Genet 2004;36(6):559–64.
6. Murali TM, Wu CJ, Kasif S. The Art of Gene Function Prediction. Nature Biotechnology 2006;12:1474–1475.
7. Ideker T, Sharan R. Protein networks in disease. Genome research 2008 4;18(4):644–652. http://dx.doi.org/10.1101/gr.071852.107.
8. Leiserson MD, Vandin F, Wu HT, Dobson JR, Eldridge JV, Thomas JL, et al. Pan-cancer network analysis identifies combinations of rare somatic mutations across pathways and protein complexes. Nat Genet 2015 Feb;47(2):106–114.
9. van Dijk D, Sharma R, Nainys J, Yim K, Kathail P, Carr AJ, et al. Recovering Gene Interactions from Single-Cell Data Using Data Diffusion. Cell 2018 07;174(3):716–729.
10. Kasif S, Roberts RJ. We need to keep a reproducible trace of facts, predictions, and hypotheses from gene to function in the era of big data. PLoS Biol 2020 11;18(11):e3000999.
11. Gordon DE, Jang GM, Bouhaddou M, Xu J, Obernier K, White KM, et al. A SARS-CoV-2 protein interaction map reveals targets for drug repurposing. Nature 2020;.
12. Szklarczyk D, Morris JH, Cook H, Kuhn M, Wyder S, Simonovic M, et al. The STRING Database in 2017: Quality-Controlled Protein-Protein Association Networks, Made Broadly Accessible. Nucleic Acids Research 2016;45(D1):D362–D368. https://doi.org/10.1093/nar/gkw937.
13. Oughtred R, Rust J, Chang C, Breitkreutz BJ, Stark C, Willems A, et al. The BioGRID database: A comprehensive biomedical resource of curated protein, genetic, and chemical interactions. Protein Science 2021;30(1):187–200. https://onlinelibrary.wiley.com/doi/abs/10.1002/pro.3978.
14. Luck K, Kim DK, Lambourne L, Spirohn K, Begg BE, Bian W, et al. A reference map of the human binary protein interactome. Nature 2020;580(7803):402–408.
15. Wishart DS, Feunang YD, Guo AC, Lo EJ, Marcu A, Grant JR, et al. DrugBank 5.0: A major update to the DrugBank

database for 2018. Nucleic Acids Research 2018;.
16. Stukalov A, Girault V, Grass V, Karayel O, Bergant V, Urban C, et al. Multilevel proteomics reveals host perturbations by SARS-CoV-2 and SARS-CoV. Nature 2021 06;594(7862):246–252.
17. Li J, Guo M, Tian X, Wang X, Yang X, Wu P, et al. Virus-Host Interactome and Proteomic Survey Reveal Potential Virulence Factors Influencing SARS-CoV-2 Pathogenesis. Med (N Y) 2021 Jan;2(1):99–112.
18. Samavarchi-Tehrani P, Abdouni H, Knight JDR, Astori A, Samson R, Lin ZY, et al. A SARS-CoV-2 – host proximity interactome. bioRxiv 2020;https://www.biorxiv.org/content/early/2020/09/04/2020.09.03.282103.
19. Hekman RM, Hume AJ, Goel RK, Abo KM, Huang J, Blum BC, et al. Actionable Cytopathogenic Host Responses of Human Alveolar Type 2 Cells to SARS-CoV-2. Mol Cell 2020 12;80(6):1104–1122.
20. Mick E, Kamm J, Pisco AO, Ratnasiri K, Babik JM, Calfee CS, et al. Upper airway gene expression differentiates COVID-19 from other acute respiratory illnesses and reveals suppression of innate immune responses by SARS-CoV-2. medRxiv 2020 May;.
21. Cowen L, Ideker T, Raphael BJ, Sharan R. Network propagation: a universal amplifier of genetic associations. Nature Reviews Genetics 2017 Sep;18(9):551–562.
22. Fouss F, Francoisse K, Yen L, Pirotte A, Saerens M. An experimental investigation of kernels on graphs for collaborative recommendation and semisupervised classification. Neural Networks 2012;31:53 – 72. http://www.sciencedirect.com/science/article/pii/S0893608012000822.
23. Page L, Brin S, Motwani R, Winograd T. The PageRank Citation Ranking: Bringing Order to the Web. Stanford InfoLab; 1999.
24. Mostafavi S, Ray D, Warde-Farley D, Grouios C, Morris Q. GeneMANIA: a Real-Time Multiple Association Network Integration Algorithm for Predicting Gene Function. Genome Biology 2008;9(Suppl 1):S4. http://dx.doi.org/10.1186/gb-2008-9-s1-s4.
25. Murali TM, Dyer MD, Badger D, Tyler BM, Katze MG. Network-based prediction and analysis of HIV dependency factors. PLoS computational biology 2011 9;7(9):e1002164+. http://dx.doi.org/10.1371/journal.pcbi.1002164.
26. Gligorijević V, Barot M, Bonneau R. deepNF: Deep Network Fusion for Protein Function Prediction. Bioinformatics 2018;34(22):3873–3881. https://doi.org/10.1093/bioinformatics/bty440.
27. Guy RK, DiPaola RS, Romanelli F, Dutch RE. Rapid repurposing of drugs for COVID-19. Science 2020 05;368(6493):829–830.
28. Wu C, Liu Y, Yang Y, Zhang P, Zhong W, Wang Y, et al. Analysis of therapeutic targets for SARS-CoV-2 and discovery of potential drugs by computational methods. Acta Pharmaceutica Sinica B 2020;.
29. Vaduganathan M, Vardeny O, Michel T, McMurray JJ, Pfeffer MA, Solomon SD. Renin–angiotensin–aldosterone system inhibitors in patients with Covid-19. New England Journal of Medicine 2020;382(17):1653–1659.
30. Zhou Y, Hou Y, Shen J, Huang Y, Martin W, Cheng F. Network-based drug repurposing for novel coronavirus 2019-nCoV/SARS-CoV-2. Cell discovery 2020;6(1):1–18.
31. Zhang Y, Zeng T, Chen L, Ding S, Huang T, Cai YD. Identification of COVID-19 Infection-Related Human Genes Based on a Random Walk Model in a Virus-Human Protein Interaction Network. BioMed research international 2020;2020.
32. Sadegh S, Matschinske J, Blumenthal DB, Galindez G, Kacprowski T, List M, et al. Exploring the SARS-CoV-2 virus-host-drug interactome for drug repurposing. Nat Commun 2020 07;11(1):3518.
33. Stoeger T, Nunes Amaral LA. COVID-19 research risks ignoring important host genes due to pre-established research patterns. Elife 2020 11;9.
34. Lee AS. Glucose-regulated proteins in cancer: molecular mechanisms and therapeutic potential. Nat Rev Cancer 2014 Apr;14(4):263–276.
35. Zhang Y, Liu R, Ni M, Gill P, Lee AS. Cell surface relocalization of the endoplasmic reticulum chaperone and unfolded protein response regulator GRP78/BiP. J Biol Chem 2010 May;285(20):15065–15075.
36. Tsai YL, Ha DP, Zhao H, Carlos AJ, Wei S, Pun TK, et al. Endoplasmic reticulum stress activates SRC, relocating chaperones to the cell surface where GRP78/CD109 blocks TGF-β signaling. Proc Natl Acad Sci USA 2018 05;115(18):E4245–E4254.
37. Ni M, Zhang Y, Lee AS. Beyond the endoplasmic reticulum: atypical GRP78 in cell viability, signalling and therapeutic targeting. Biochem J 2011 Mar;434(2):181–188.
38. Booth L, Roberts JL, Cash DR, Tavallai S, Jean S, Fidanza A, et al. GRP78/BiP/HSPA5/Dna K is a universal therapeutic target for human disease. J Cell Physiol 2015 Jul;230(7):1661–1676.
39. DeDiego ML, Nieto-Torres JL, Jiménez-Guardeño JM, Regla-Nava JA, Alvarez E, Oliveros JC, et al. Severe acute respiratory syndrome coronavirus envelope protein regulates cell stress response and apoptosis. PLoS Pathog 2011 Oct;7(10):e1002315.
40. Chan CP, Siu KL, Chin KT, Yuen KY, Zheng B, Jin DY. Modulation of the unfolded protein response by the severe acute respiratory syndrome coronavirus spike protein. J Virol 2006 Sep;80(18):9279–9287.
41. Chu H, Chan CM, Zhang X, Wang Y, Yuan S, Zhou J, et al. Middle East respiratory syndrome coronavirus and bat coronavirus HKU9 both can utilize GRP78 for attachment onto host cells. J Biol Chem 2018 07;293(30):11709–11726.
42. Lyoo HR, Park SY, Kim JY, Jeong YS. Constant up-regulation of BiP/GRP78 expression prevents virus-induced apoptosis in BHK-21 cells with Japanese encephalitis virus persistent infection. Virol J 2015 Feb;12:32.
43. Ibrahim IM, Abdelmalek DH, Elshahat ME, Elfiky AA. COVID-19 spike-host cell receptor GRP78 binding site prediction. J Infect 2020 05;80(5):554–562.
44. Aguiar JA, Tremblay BJM, Mansfield MJ, Woody O, Lobb B, Banerjee A, et al. Gene expression and in situ protein profiling of candidate SARS-CoV-2 receptors in human airway epithelial cells and lung tissue. bioRxiv 2020;https://www.biorxiv.org/content/early/2020/04/12/2020.04.07.030742.
45. Terpos E, Ntanasis-Stathopoulos I, Elalamy I, Kastritis E, Sergentanis TN, Politou M, et al. Hematological findings and complications of COVID-19. Am J Hematol 2020 Apr;.
46. Kaufman RJ, Pipe SW, Tagliavacca L, Swaroop M, Moussalli M. Biosynthesis, assembly and secretion of coagulation factor VIII. Blood Coagul Fibrinolysis 1997 Dec;8 Suppl 2:3–14.
47. Flemmig M, Melzig MF. Serine-proteases as plasminogen activators in terms of fibrinolysis. J Pharm Pharmacol 2012 Aug;64(8):1025–1039.
48. Deng WG, Ruan KH, Du M, Saunders MA, Wu KK. Aspirin and salicylate bind to immunoglobulin heavy chain binding

protein (BiP) and inhibit its ATPase activity in human fibroblasts. FASEB J 2001 Nov;15(13):2463–2470.
49. Nogales E. Structural insights into microtubule function. Annu Rev Biochem 2000;69:277–302.
50. Greber UF, Way M. A superhighway to virus infection. Cell 2006 Feb;124(4):741–754.
51. Rüdiger AT, Mayrhofer P, Ma-Lauer Y, Pohlentz G, Müthing J, von Brunn A, et al. Tubulins interact with porcine and human S proteins of the genus Alphacoronavirus and support successful assembly and release of infectious viral particles. Virology 2016 10;497:185–197.
52. Satir P, Christensen ST. Overview of structure and function of mammalian cilia. Annu Rev Physiol 2007;69:377–400.
53. Lee IT, Nakayama T, Wu CT, Goltsev Y, Jiang S, Gall PA, et al. Robust ACE2 protein expression localizes to the motile cilia of the respiratory tract epithelia and is not increased by ACE inhibitors or angiotensin receptor blockers. medRxiv 2020;https://www.medrxiv.org/content/early/2020/05/12/2020.05.08.20092866.
54. Sungnak W, Huang N, Bécavin C, Berg M, Queen R, Litvinukova M, et al. SARS-CoV-2 entry factors are highly expressed in nasal epithelial cells together with innate immune genes. Nat Med 2020 05;26(5):681–687.
55. Puelles VG, Lütgehetmann M, Lindenmeyer MT, Sperhake JP, Wong MN, Allweiss L, et al. Multiorgan and Renal Tropism of SARS-CoV-2. N Engl J Med 2020 May;.
56. Huang C, Wang Y, Li X, Ren L, Zhao J, Hu Y, et al. Clinical features of patients infected with 2019 novel coronavirus in Wuhan, China. Lancet 2020 02;395(10223):497–506.
57. Wei J, Alfajaro MM, Hanna RE, DeWeirdt PC, Strine MS, Lu-Culligan WJ, et al. Genome-wide CRISPR screen reveals host genes that regulate SARS-CoV-2 infection. bioRxiv 2020;https://www.biorxiv.org/content/early/2020/06/17/2020.06.16.155101.
58. Daniloski Z, Jordan TX, Wessels HH, Hoagland DA, Kasela S, Legut M, et al. Identification of required host factors for SARS-CoV-2 infection in human cells. Cell 2020;.
59. Ribeiro MT, Singh S, Guestrin C. "Why should i trust you?" Explaining the predictions of any classifier. In: Proceedings of the 22nd ACM SIGKDD International Conference On Knowledge Discovery and Data Mining; 2016. p. 1135–1144.
60. Pearl J. Causality. Cambridge university press; 2009.
61. Mostafavi S, Ray D, Warde-Farley D, Grouios C, Morris Q. GeneMANIA: a real-time multiple association network integration algorithm for predicting gene function. Genome Biology 2008;9 Suppl 1:S4.
62. Vanunu O, Magger O, Ruppin E, Shlomi T, Sharan R. Associating genes and protein complexes with disease via network propagation. PLoS computational biology 2010 1;6(1):e1000641+. http://dx.doi.org/10.1371/journal.pcbi.1000641.
63. Komurov K, White MA, Ram PT. Use of data-biased random walks on graphs for the retrieval of context-specific networks from genomic data. PLoS Comput Biol 2010;6(8).
64. Jiang B, Kloster K, Gleich DF, Gribskov M. AptRank: an adaptive PageRank model for protein function prediction on bi-relational graphs. Bioinformatics 2017 6;33(12):1829–1836. http://dx.doi.org/10.1093/bioinformatics/btx029.
65. Hristov BH, Chazelle B, Singh M. uKIN Combines New and Prior Information with Guided Network Propagation to Accurately Identify Disease Genes. Cell Syst 2020 06;10(6):470–479.
66. Letovsky S, Kasif S. Predicting protein function from protein/protein interaction data: a probabilistic approach. Bioinformatics (Oxford, England) 2003 7;19 Suppl 1(suppl 1):i197–i204. http://dx.doi.org/10.1093/bioinformatics/btg1026.
67. Deng M, Tu Z, Sun F, Chen T. Mapping Gene Ontology to proteins based on protein-protein interaction data. Bioinformatics 2004;20(6):895–902.
68. Nabieva E, Jim K, Agarwal A, Chazelle B, Singh M. Whole-proteome prediction of protein function via graph-theoretic analysis of interaction maps. Bioinformatics 2005 June;21 Suppl 1:i302–i310.
69. Goranci G, Henzinger M, Thorup M. Incremental exact min-cut in polylogarithmic amortized update time. ACM Transactions on Algorithms (TALG) 2018;14(2):1–21.
70. Kshirsagar M, Tasnina N, Ward MD, Law JN, Murali TM, Lavista Ferres JM, et al. Protein sequence models for prediction and comparative analysis of the SARS-CoV-2 -human interactome. Pac Symp Biocomput 2021;26:154–165.

Supplementary Text

Click here to access/download
Supplementary Material
sars-cov-2-networks-supplement.pdf

File S1

Click here to access/download
Supplementary Material
File-S1.tsv

File S2

Click here to access/download
Supplementary Material
File-S2.tsv

File S3

Click here to access/download
Supplementary Material
File-S3.tsv

Click here to access/download;Personal Cover;2021-09-sars-cov-2-networks-GigaScience-response-to-reviews.pdf

Response to Reviewers
Interpretable Network Propagation with Application to Expanding the Repertoire of Human Proteins that Interact with SARS-CoV-2

We thank the reviewers for their insightful comments and suggestions. We made a few changes to the manuscript and added several analyses to the supplement. We highlight these changes in the manuscript using this color.

The changes in the main manuscript include restructuring "Analysis" to give more importance to our main contributions on provenance tracing:

1. *We shortened the subsection "Prioritization of Potential SARS-CoV-2 Interactors" by moving the cross validation results to the supplementary results. Consequently, this subsection contains the evaluations of predictions made by RL in terms of overlap with other datasets and functional enrichment. We retained these analyses in the main text since they confirm that our predictions are biologically meaningful and relevant to the virus.*
2. *We reordered the section "Tracing the Provenance of Top-Ranking Proteins" by moving the analysis of alpha below the main results for our provenance tracing approach.*

The analyses we added are as follows:

1. *Results for Random Walk with Restarts (RWR) in the sections on cross-validation (CV), parameter selection, and overlap among algorithms.*
2. *Comparison to PPI networks (BioGRID, BioGRID-Y2H, HI-union) for the effective diffusion results.*
3. *Overlap of our top-ranking proteins with two more experimentally determined sets*
    a. *Differential protein abundance in SARS-CoV-2–infected iAT2 cells.*
    b. *Differential gene expression in upper airway samples in SARS– CoV-2–infected patients*

Finally, we divided the Supplementary Information into three parts: Supplementary Methods, Supplementary Results, and Supplementary Figures.

We address each reviewer's comments in detail below.

Reviewer 1

The article provides information, useful and verifiable; presenting an adequate methodological approach applied to the study of the most important public health problem that now urges us. However, unlike what the authors argue in some sections, I do not consider it very original, as it is only a refinement of what has been done in propagation studies in networks for some time, but without a doubt its results are valuable,

which more than justifies its publication. On the other hand, the mathematical description of the algorithm is quite clear, much clearer than what is described in the text of the document, which I suggest to review, as it is difficult in some sections.

We appreciate the reviewer's viewpoint. We hope that the reviewer finds that our restructuring of the text and edits have improved the clarity of the presentation.

Reviewer 2

The authors designed a network propagation framework with two novel components and applied it to predict human proteins that directly or indirectly interact with SARS-CoV-2 proteins. There are several major problems:

1. There have been several similar studies which identified covid-19 infection-related human genes based on a random walk model in a virus-human protein interaction network (PMID: 32685484). The authors need to compare with previous works.

Thank you for this suggestion. Since the cited manuscript uses RWR as the primary algorithmic component, we also executed RWR using the same source nodes (i.e., human proteins that interact with SARS-CoV-2 proteins), and found that it produced similar results to RL. We added a citation to this specific paper suggested by the reviewer in a paragraph on related work in "Discussion".

2. The authors used network-based method to predict SARS-CoV-2 interaction protein. But there is plenty of gene expression data. The authors should compare the network genes with expression genes, such as PMID: 33505977 and 33519902.

We appreciate the recommendation to compare network-based predictions with differential gene expression data. We have added a paragraph to "Analyses" discussing the overlap between our top-ranking predictions and experimental data sets identifying differential protein abundance and gene expression in response to SARS-CoV-2 infection. We have updated Figure 2(a) with the results for protein abundance and added Supplementary Figure S8 for the overlap with differential gene expression data. We have also cited the relevant papers that published the original experimental data.

3. Within the network methods, there are network embedding (PMID: 31455874), network diffusion (PMID: 30069494), random walk with restart (PMID: 28787010, 28932904), shortest path (PMID: 28076954, 27434024, 26209080, 28974058, 23762832, 24056857, 22496748) and guilt-by-association (doi: 10.1016/j.neucom.2015.09.136). These methods should be introduced.

Thank you for this idea. Please note that the first seven papers that we have already cited in our manuscript are among the earliest in the field of network biology (published between 2003 and 2008) that use ideas in network diffusion, random walks, and guilt by association. We also cite an early review in the field that covers these topics, including shortest paths. In addition, the first paper we cite in "Analyses" is a more recent review that covers the topics mentioned by the reviewer. Our perspective is that it is important to cite such papers that may have influenced the field.

In this work, we compared a specific, representative set of network methods, including network embedding (deepNF), network diffusion (RL, GeneMANIA, and RWR; note that RWR is in fact a network diffusion method), and guilt-by-association (Local). We chose not to compare with shortest path approaches since network propagation methods implicitly consider all paths in the network, with parameters (e.g., the restart parameter for RWR) controlling for the relative importance of short vs long paths.

4. The supplementary materials are different from the descriptions of File S1 … Figure S4. Please check them carefully.

Thank you for pointing out this error. We have checked and corrected the supplementary files. Please note that there are only three files now.

Reviewer 3

In their manuscript, Law and colleagues use network propagation to predict potential SARS-CoV-2-interacting human proteins. On the methods front, they propose a means to (1) track down the prioritization contributions of individual seed proteins experimentally documented to interact with the virus; (2) select the main parameter of their network propagation method of choice (regularized Laplacian - RL) in an unbiased manner by relating it to the expected shortest path length of the random walk.

Overall, the individual sections of the paper are clear and well-written. However, taken together, the two main analyses somewhat lack a unifying narrative and feel disconnected from each other. While the methodological contributions of the paper seem interesting, the core choice of methods in the application to SARS-CoV-2 seems inadequately justified. Below are my two major concerns pertaining to each part of the analysis.

We very much appreciate the reviewer's careful inspection of our work and considerable effort put into their review. We hope that the substantial restructuring of the manuscript and additional analyses and rationale we have provided address the concerns raised by the reviewer.

Major comments

1) In general, in the first (prioritization) part of the analysis, it is not clear to me what the actual goal is. RL is benchmarked against several types of network propagation-based and "traditional" prioritization approaches, then SVM is taken as a representative of the latter group. Proteins are then prioritized between these two methods and validated in silico by their overlap with literature and their functional annotations. As it stands, the point of discussing and comparing different methods is not clear. What makes RL stand out is not directly evident the way these results are presented. In fact, the entire first part of the results, in my view, does not make a sufficient case for the use of network propagation as the prioritization method of choice. It rather looks like other off-the-shelf methods perform comparably (Fig. 2a), and yield similar biological insights (Fig 2c). It seems to me that if comparison of methods was performed, the authors should at least focus on the unique insights provided by RL (e.g. in terms of GO terms), if any, compared to SVM.

We acknowledge the reviewer's point. Our main goal was to apply the RL and the provenance tracing method we developed to predict potential interactors of SARS-CoV-2. Rather than use RL directly, we first compared it to many different approaches to solve the same problem. Based on the reviewer's comments, we have statistically compared the distributions of AUROC, AUPRC, and precision at 0.3 recall of all pairs of algorithms in "Comparison of Cross-Validation Results" in the Supplementary Results. RWR and deepNF had slightly higher AUPRC and precision at 0.3 recall than RL for the 1:5 and 1:10 positive:negative ratios but not for the 1:1 ratio. Nevertheless, we selected RL (and SVM) for subsequent analyses as we explain in the main manuscript:

> *"To decide which methods to select for subsequent analyses,we compared them using 5-fold cross validation ("Comparison of Cross-Validation Results" in the supplementary text and Figure S1). RL, random walk with restarts (RWR) [23], and deepNF [26] had the highest values of area under the precision-recall curve followed by SVM and logistic regression. RL achieved marginally worse values of area under the precision-recall curve than RWR and deepNF. We selected one network propagation method (RL) and one supervised classifier (SVM) for the following reasons. We preferred RL over deepNF because the provenance tracing method we developed for RL enabled its results to be more easily interpreted than those for deepNF. Since RL and RWR produced highly similar predictions with a very high Spearman's correlation for the ranking of all proteins ("Overlap among algorithms" in the supplementary text and Figure S2), we selected RL as representative of the two methods. We chose SVM among the two off-the-shelf classifiers since it also had very good performance in cross-validation."*

This reasoning also addresses the question that the reviewer raises about the choice of RL in the next comment. We acknowledge that using RWR instead of RL may have yielded similar results for provenance tracing. We intend to perform this analysis in a subsequent project.

2) Provenance tracing explored in the second part is very interesting as a premise, and the fact that it is relatively uninvestigated makes it an appealing topic. As the authors note, it is based on a simple principle: once the regularized Laplacian kernel is calculated, the values of the kernel matrix are row-sorted for each protein. While this is still, in a sense, a steady-state view, and the name "provenance tracing" inspires in the reader's mind a sequence of connections between seeds and targets, I think the authors did a good job showcasing how it simplifies the subnetworks related to each biological process of interest, improving interpretability. I am convinced by its utility in that sense. What I have been having trouble convincing myself about is the particular choice of RL, some claims around the novelty of the analytical results, and why \alpha matters in the first place. To elaborate:

2a) The rationale presented for putting emphasis on \alpha seems not so well-justified to me. The authors state that they looked for a different way to determine alpha than looking at AUROC/AUPRC values since they varied little over a wide range of alpha. **Does this not mean that the choice of alpha does not impact the prediction results in the case of RL?** I guess I don't fully understand the point of the derivation other than a purely mathematical exercise (whose precedents seem to exist - see below). This also bears asking why RL was chosen in the first place. It sounds in the paper as if RL is the only choice of kernel that can be utilized this way to compute the contribution of the seed nodes, whereas many other types of kernels should work in a similar manner (see Fouss et al. cited by the authors as [18]). **It seems that for provenance tracing, any type of kernel could work as the authors simply use the values corresponding to the seeds at the steady state kernel matrix to represent the contribution of each**

seed on the candidate protein's score. For example, RL is indeed similar to RWR, a widely used propagation method in biology (see Köhler, Sebastian, et al. "Walking the interactome for prioritization of candidate disease genes." The American Journal of Human Genetics 82.4 (2008): 949-958), in terms of the form of its kernel. **deepNF, one of the methods considered in the benchmark that does comparably or better than RL, is also based on RWR. Could the authors comment on the above points?**

(We have highlighted in bold the key questions asked by the reviewer in this comment.)

We agree with the reviewer that the choice of alpha does not impact the predictions in the case of RL. It was important for us to have tested different values of alpha before reaching this conclusion. Although varying alpha did not have a major effect on the predictions for RL in this particular application, it may have a larger impact for other prediction problems.

We also agree that the RL is not the only kernel to which this type of provenance tracing is applicable. We mention this point at two places in the manuscript:
(i) Abstract: "We examine how our provenance tracing method can be generalized to a broad class of network-based algorithms."
(ii) Discussion: "Our strategy for tracing provenance extends to any algorithm that makes predictions using a linear combination of evi- dence such as logistic regression and GeneMania [61]. In particular, it is applicable to the large number of random-walk-based methods that have been developed for predicting disease genes or annotations to GO terms [62, 63, 64, 65]."

Although deepNF is based on RWR, it uses an autoencoder to learn a low-dimensional embedding of the input network and an SVM with an RBF kernel trained on this embedding to solve the node labeling problem. Developing a provenance tracing strategy for deepNF is non-trivial and will require a substantial research effort.

2b) The authors take a mean-field approach relating the teleportation/damping parameter (\alpha) to the expected value of path lengths given a network. The authors then use the actual network to determine the median path length between seeds and candidates and find the \alpha that corresponds to this value from their precomputed lookup table. In a way, we can see this parameter selection process as the tuning of the random walk according to the network at hand. Regarding the analytical results on average path lengths in random walks/diffusion processes, similar results seem to exist:

- Yazdani, Majid, and Andrei Popescu-Belis. "A random walk framework to compute textual semantic similarity: a unified model for three benchmark tasks." 2010 IEEE Fourth International Conference on Semantic Computing. IEEE, 2010.
- Ghosh, Rumi, et al. "Non-conservative diffusion and its application to social network analysis." arXiv preprint arXiv:1102.4639 (2011).
- Ghosh, Rumi, and Kristina Lerman. "Rethinking centrality: the role of dynamical processes in social network analysis." arXiv preprint arXiv:1209.4616 (2012).
- Stojmirović, Aleksandar, and Yi-Kuo Yu. "Information flow in interaction networks." Journal of Computational Biology 14.8 (2007): 1115-1143.

- Masuda, Naoki, Mason A. Porter, and Renaud Lambiotte. "Random walks and diffusion on networks." Physics reports 716 (2017): 1-58.

Could the authors elaborate on the difference of their approach and scope?

We appreciate the referee's attention to this aspect of our work, which encourages us to expand on the motivation and novelty of our analysis.

The use of diffusion and random walks (broadly, "stochastic processes") as tools in network analysis is extensive. However, a dimension that is not always emphasized in such analyses is the distinction between processes that evolve in discrete time (eg, "random walks" that evolve in "steps" or "iterations") versus those that evolve in continuous time (eg, "diffusion" that evolves via "fluid flow"). These two kinds of processes require different analytic tools. Discrete-time processes (as exemplified by PageRank and random-walk-with-restarts) are more common, and their analysis is often simpler. However, the Regularized Laplacian (RL) that we rely on corresponds to the solution of a continuous-time process (eg, it can be described in terms of a fluid flow). The tools for analyzing such processes are based on continuous-time Markov chains (CTMCs), ie, systems of differential equations.

As noted by the referee, our work tunes the mean path length of a time-bounded continuous time random walk to match average path lengths in biological processes. To do so, the analytic challenge we overcome comes from the fact that path lengths take on discrete values, while the ***random walk evolves in continuous time****, and the* ***time bound is a random variable.***

We note that even specialized references do not contain discussion of expected path lengths in the CTMC corresponding to a Regularized Laplacian. For example, the question is not discussed in the recent, comprehensive text "Algorithms and Models for Network and Link Analysis," (Fouss et al). Before writing up our analysis, we searched the literature and contacted experts in the field of stochastic processes to discern if this problem had previously been solved. We found no evidence that it had been addressed before.

With respect to the papers pointed to by the referee, each does indeed contain consideration of path lengths, but most are concerned only with models based on discrete time. None of the papers considers our question specifically. Thus the results of those papers cannot be used to answer the question we address.

Specifically:

- Yazdani, Majid, and Andrei Popescu-Belis. "A random walk framework to compute textual semantic similarity: a unified model for three benchmark tasks." 2010 IEEE Fourth International Conference on Semantic Computing. IEEE, 2010.

  This paper uses "hitting time" and "commute time" as measures of walk length. Although these terms involve the word "time", here time is understood to be a discrete quantity. For example, hitting time (i,j) is the mean number of steps in a discrete time random walk starting at i, up to the first visit to node j.

- Ghosh, Rumi, et al. "Non-conservative diffusion and its application to social network analysis." arXiv preprint arXiv:1102.4639 (2011).

  This paper studies "non-conservative" diffusion, which can also be described as epidemic modeling. That model is fundamentally different from a random walk. This paper too is only concerned with discrete time processes.

- Ghosh, Rumi, and Kristina Lerman. "Rethinking centrality: the role of dynamical processes in social network analysis." arXiv preprint arXiv:1209.4616 (2012).

  This paper, a precursor to the Ghosh et al paper above, is also concerned with discrete time random walks. Furthermore, the path lengths that it analyzes are paths in a non-conservative process, that is, paths of infection in an epidemic.

- Stojmirović, Aleksandar, and Yi-Kuo Yu. "Information flow in interaction networks." Journal of Computational Biology 14.8 (2007): 1115-1143.

  This paper includes discussion of a diffusion process and average path lengths. However the diffusion process is specific to this paper (not a commonly-used process like the RL) and path lengths are observed empirically (ie, no mathematical expression for path length is derived).

- Masuda, Naoki, Mason A. Porter, and Renaud Lambiotte. "Random walks and diffusion on networks." Physics reports 716 (2017): 1-58.

  This paper, in contrast to those above, extensively covers both discrete and continuous time stochastic processes. However, the specific question we ask, namely, the mean path length in the RL model, is not addressed in this paper. This underscores that our problem is not just one of computing the probability of reaching a given node in a given amount of time (which this paper does address) but specifically of ***how many nodes the walker will pass through in a random amount of continuous time.***

Minor comments

Aside from the above major concerns, below are my minor points and comments to help the authors improve their work:

3) In the Discussion: "We were surprised to see that the top-contributing sources were invariably direct neighbours of the top-ranking predictions in the STRING network. A partial explanation for this trend may be the fact that as many as 5,331 proteins in the STRING network were direct neighbors of at least one source protein, even when we considered only interactions with weight at least 0.9 (the STRING database deems edges with such weights to be of "very high quality"). Thus, the structure of the STRING network and central location of sources within it may cause the RL both to give high ranks only to direct neighbors of sources and to channel propagation primarily along these direct connections."
To recap, this part of the discussion is related to the fact that, even though global exploration by using teleports is favored over local neighbor-hopping by setting \alpha to a high value, the top contributions

still come from directly connected seeds. To me, this is one of the most interesting findings in the paper that might also call into question the widespread use of diffusion-based prioritization on PPI networks if it really is the case that, no matter how globally explored the network is, it is still the seeds directly neighboring the prioritized proteins that affect the prioritization the most. One question related to that is whether or not the same holds for proteins that were lowly ranked by RL, i.e. is RL (and potentially other random-walk based methods) capturing chiefly "local" contributions from seeds for proteins at the bottom of the list as well? I feel that this can be explored further by (1) trying PPI networks with different densities and degree distributions than STRING such as strictly experimental binary PPIs such as those derived from Y2H assays (e.g. Luck, Katja, et al. "A reference map of the human binary protein interactome." Nature 580.7803 (2020): 402-408.); (2) testing whether the number of seeds itself (in this case around 300 if I followed the methods correctly) is a contributing factor to this phenomenon where, as the authors noted in the discussion, the direct interactions of these seed proteins cover the majority of the network, "saturating" the random walk process in a sense. Would we see the same results if there were, say, only 30 seed proteins instead of 300? In general, I would request the authors to think a little bit to delve a little further into this, without disrupting the flow of the paper as it is now.

Thank you for these suggestions. We found the first idea to be appealing. We repeated our experiments with three PPI networks: BioGRID, BioGRID with Y2H assays only, and the high-quality HI-union network from the paper the reviewer suggested. We originally hypothesized that the effective diffusion would be higher for the top-ranking proteins of these networks, but surprisingly, the influence from direct neighbors was even greater for these networks, resulting in lower effective diffusion values (see Figures 3(f), S9, and Table 1). We discuss these results in the Analysis section.

We believe these points merit further investigation and plan to continue this research in a future paper.

4) Figure 1 seems too generic. Perhaps include some more details such as what the nodes are (red, virus, blue human proteins, etc.), node sizes, etc.

We have added a legend to the figure on the node colors and gradients.

5) Figure 2a - I could not find anywhere if the AUROC/AUPRC values are statistically significantly different between different methods. The median values are compared but it seems by looking at the error bars that the difference is perhaps not statistically significant with respect to the other methods.

We tested for statistical significance between the algorithms and added a discussion in the supplementary results section in "Comparison of Cross-Validation Results."

6) Figure 2a caption: precision at 0.1 recall or 0.3 recall (latter one used in the text, former used in the caption)

We corrected the caption to "precision at 0.3 recall".

7) Figure 2C - top-ranked, meaning top 332 or top 1000?

Figure 2C (now B) shows the top 332. We updated the text to clarify this difference.

8) The provenance tracing part of the analysis: an intuitive definition of \alpha would be helpful at the beginning of this section. Currently, it is introduced without such an explanation as to what it does, such as the damping or "teleportation" parameter equivalent in pagerank.

At the beginning of the section "Tracing the Provenance of Top-Ranking Proteins," we attempt to give an intuitive idea of the effect of \alpha: "Hence for larger values of \alpha, the influence of the sources is diffused more broadly across the network."

9) The flow of this results section should be revisited. The authors lead with a discussion on the sensitivity analysis of \alpha, which, in my opinion, is of secondary importance to the provenance tracing aspect. The most important part of the paper thus gets buried further down into the results section.

We agree with the reviewer's suggestion. We have reorganized the results section so that provenance tracing comes earlier.

10) Figure 3A, figure order (comes after Figs 3B-E) in the text

We updated the figure order.

11) "The GO biological process "protein folding in endoplasmic reticulum" was also enriched in the top-ranking proteins (p-value 4.32 10-9 for RL and 0.28 for interactors of SARS-CoV-2)."
This sentence reads as if the ER related GO terms were identified through two independent processes, where in reality it was the GO term enrichment on the top-prioritized proteins that was done first and "protein folding on ER" was identified as a process of interest, and then provenance tracing was performed on this biological process because it was implicated by the enrichment analysis in the first place. Minor point but one pertains to the flow of the text nevertheless: I think it would be helpful to remind the reader the order of events that led to these results, i.e. first the identification of salient pathways of GO terms, and then a detailed x-ray of these pathways through provenance tracing.

We added the following sentence to the paragraph just before the section "The Role of Endoplasmic Reticulum Stress, HSPA5, and Anti-Clotting Drugs":

> *"We remind the reader that we computed functions enriched in the top-ranking proteins, performed the provenance analysis independently, and then integrated the results in the protein networks we visualized."*

12) Typo: "we compute the precise contribution of each source's contribution to the score of u."

We have corrected it.
