# Supplementary material for: Interpretable network propagation with application to expanding the repertoire of human proteins that interact with SARS-CoV-2
Source: Gigascience. 2021 Dec 29;10(12):giab082. doi: 10.1093/gigascience/giab082 (PMC8716363; doi:10.1093/gigascience/giab082)
Supplement: giab082_Supplemental_Files [file giab082_supplemental_files.zip › sars-cov-2-networks-supplement.pdf]

GigaScience, 20xx, 1–15

doi: xx.xxxx/xxxx
Manuscript in Preparation

Supplementary Information
Interpretable Network Propagation with Application to Expanding the Repertoire of Human Proteins that Interact with SARS-CoV-2

Jeffrey N. Law[1], Kyle Akers[1], Nure Tasnina[2], Catherine M. Della Santina[3], Shay Deutsch[4], Meghana Kshirsagar[5], Judith Klein-Seetharaman[6], Mark Crovella[7], Padmavathy Rajagopalan[8], Simon Kasif[3] and T. M. Murali[2,*]

[1]Interdisciplinary Ph.D. Program in Genetics, Bioinformatics, and Computational Biology, Blacksburg, VA, USA and [2]Department of Computer Science, Virginia Tech, Blacksburg, VA, USA and [3]Department of Biomedical Engineering, Boston University, Boston, MA, USA and [4]Department of Mathematics, University of California, Los Angeles, CA, USA and [5]AI for Good Lab, Microsoft, Redmond, WA, USA and [6]Department of Chemistry, Colorado School of Mines, Golden, CO USA and [7]Department of Computer Science, Boston University, Boston, MA, USA and [8]Department of Chemical Engineering, Virginia Tech, Blacksburg, VA, USA

[*] Corresponding author: murali@cs.vt.edu

Compiled on: November 14, 2021.
Draft manuscript prepared by the author.

Supplementary Information

Supplementary Figures

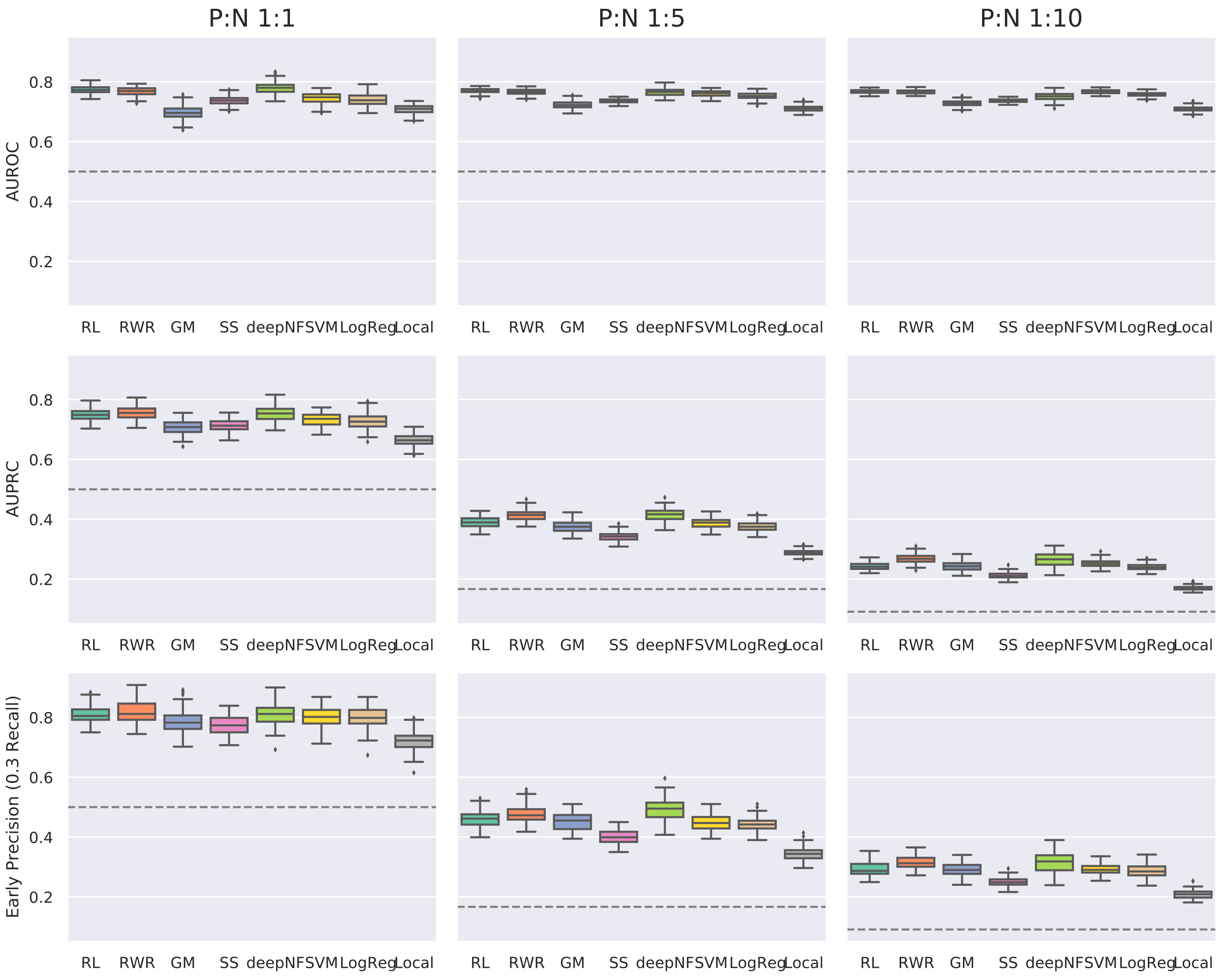

Figure S1. Cross validation results for positive:negative ratios of 1:1, 1:5 and 1:10.

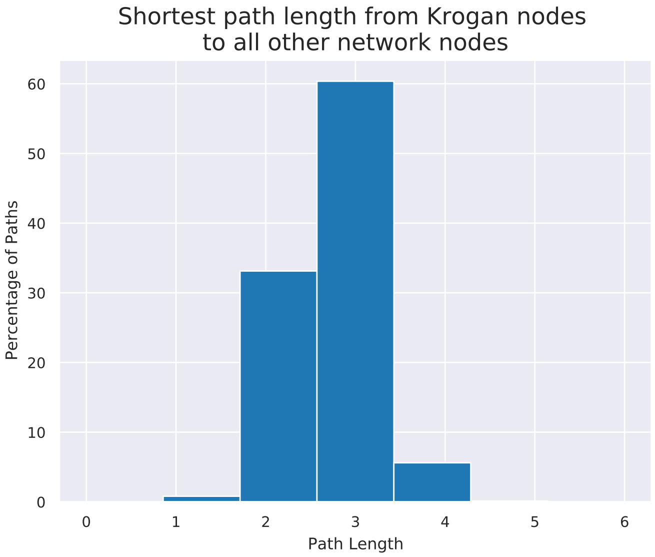

Figure S2. Distribution of path lengths from SARS-CoV-2 interactors to every other protein in the STRING network.

Supplementary Tables

α	Expectation
0.01	0.0101
0.1	0.0993
0.5	0.4790
1	0.936
2	1.824
3	2.695
3.1	2.782
3.2	2.868
3.3	2.955
**3.4**	**3.041**
3.5	3.128
3.6	3.214
3.7	3.299
3.8	3.386
3.9	3.472
4	3.558
6	5.270
8	6.973
10	8.671

Table S1. Variation of expected path length with α.

Supplementary Text

Other Algorithms

GeneMANIA [1]. This method is a variation of RL that also takes a set N of negative examples as input.

i. Compute a label vector $\vec{y}$ over the nodes in G where $y(u) = 1$ if node u is in P, $y(u) = -1$ if u is in N, and $y(u) = \frac{|P|-|N|}{|P|+|N|}$ otherwise.
ii–vi. These steps are identical to the RL algorithm.

Note that the original version of GM implicitly chose $\alpha = 1$. We introduce the parameter α to allow a tradeoff in the importance given to the input node labels $\vec{y}$ vis-a-vis the similarity of adjacent labels in the output $\vec{s}$. Every element in $\vec{s}$ has a value between -1 and 1.

SinkSource [2, 3]. This method takes only positive examples as input, like RL. Unlike RL, it fixes the scores of the positive examples. It takes a parameter $\lambda > 0$ and consists of the following steps:

i. Set $s(u) = 1$ for every positive example in P.
ii. Define U to be the set of unlabeled nodes in G, i.e., $U = V - P$.
iii. Add an artificial node t to G, set $s(t) = 0$ (i.e., treat t as a negative example), and connect every node in U to t with an edge whose weight is λ.
iv. Define D as a diagonal matrix with $D_{uu} = \sum_v w_{uv}$, for every node u in G.
v. Define the vector $\vec{f} \in \mathbb{R}^{|U|}$ as follows: for every node $u \in U$, $f(u)$ equals the sum of the weighted scores of its neighbors that are positive examples, i.e.,

$$f(u) = \sum_{\substack{v \in N(u) \\ v \text{ is positive}}} \frac{w_{uv}}{D_{uu}},$$

where $N(u)$ denotes the neighbors of u.
vi. Let W_U equal the submatrix of W corresponding to the subgraph of G induced by U and let D_U be the diagonal matrix equal to the submatrix of D corresponding to the nodes in U.
vii. Compute $\vec{s} = (I - D_U^{-1} W_U)^{-1} \vec{f}$.

The scores computed by this algorithm minimize the following function:

$$\sum_{(u,v) \in E} w_{uv} (s(u) - s(v))^2.$$

Local. We set $s(v) = 1$ for every node in P as in the case of SS. For every other node u, we initialise $s(u) = 0$ and then compute $s(u)$ as the weighted average of the scores of its neighbors in $D_U^{-1} W_U$.

deepNF [4]. deepNF uses an autoencoder to integrate multiple interaction networks into low-dimensional embeddings of the proteins. deepNF consists of four main steps:

i. For each network and for each node, perform a random walk with restarts (RWR) to that node.
ii. For each network, create a matrix where each row corresponds to a node and stores the stationary probabilities of the relevant RWR. Compute the Positive Pointwise Mutual Information (PPMI) between every pair of rows. Store these values in a (symmetric) matrix where each row stores the PPMI values for one node.
iii. Train a multimodal deep autoencoder to learn a single integrated representation of the PPMI matrices for all the networks. Treat the output of the encoder (the innermost layer of the autoencoder) as a low-dimensional representation of the proteins.
iv. Use this representation to train an SVM classifier that predicts the label of interest, which in our case is whether a human protein interacts with SARS-CoV-2 or not.

We applied the deepNF framework using the same steps and parameters as used by the authors in their original work. For step one, we set $\alpha = 0.98$ and the maximum number of transitions in the random walk to three. For step three, we used a 3-layer architecture with 2,500 nodes on the outer two layers, and 1,200 nodes for the inner layer. We also used the default number of epochs (80), batch size (64) and noise factor (0.5) for training the autoencoder.

We made two modifications. First, rather than use a subset of the STRING channels for step one, we provided every STRING channel as a network to be integrated by deepNF. We did so since the STRING network we provided to the other methods was computed by combining the scores from each individual channel. Second, for step four, we chose to train a standard linear kernel rather than a radial basis function (RBF) kernel.

Support Vector Machine. We set each node's feature vector to be its adjacency vector in the matrix $\tilde{W}$ that we computed in the RL algorithm. We trained a linear kernel using the `LinearSVC` function in the `scikit-learn` Python package (v0.22.1) with default parameters.

Logistic Regression. We set each node's feature vector as in the case of SVM. We used the `LogisticRegression` function in the `scikit-learn` Python package with default parameters.

Implementation. We implemented RL, GM, and SS in Python (v3.7.7) using the SciPy (v1.4.1) and NumPy (v1.18.2) libraries. To solve the RL and GM linear systems, we utilized the conjugate gradient solver with SciPy's default tolerance cutoff of 10^{-5}. To compute the scores for SS, we used power iteration (i.e., $\vec{s}^{(i+1)} = D_U^{-1} W_U \vec{s}^{(i)} + \vec{f}$) with $\vec{s}^{(0)} = 0$ and stopped after 100 iterations. For deepNF, we used the Python code available at https://github.com/VGligorijevic/deepNF, which relies on the Keras package (v2.4.3).

Statistical Significance of Node Scores

To estimate the statistical significance of each node's scores, we adopted a null hypothesis corresponding to the distribution of scores obtained from a randomly chosen positive set P' where $|P'| = |P|$. (As an alternative to randomizing the positive set, randomizing the network (e.g., via a degree-preserving edge swap process) would destroy the correlations between adjacent nodes (homophily) that are important contributions to pathway and neighborhood structure in the network.) We note that the degrees of the nodes in P may have a strong effect on the resulting distribution of scores. For example, if many nodes in P have high degree, then scores may tend to be larger overall than if there are few nodes in P with high degree. Thus if the degree distribution of P does not approximately match that of P', the resulting p-values will be biased.

Had we selected each random sample uniformly at random

from all nodes in G, then the degree distribution of the chosen nodes would not be ensured to match that of the nodes in P. Therefore, we implemented a stratified sampling approach, as follows: Given a number of bins b, we partitioned the nodes in G into b sets as follows:

i. We sorted the nodes by weighted degree.
ii. We executed k-means clustering on the degree sequence of G to compute b clusters (i.e., we set $k = b$ in the k-means algorithm).

This approach emphasizes nearly-equal-degree groups. Then, to generate a random sample P' having $|P|$ nodes, for every positive example v in P, we determined the subset whose range endpoints contained v and sampled a node from that subset uniformly at random. After evaluating various values of b, we selected $b = 10$ for use in our results (see "Parameter Selection" in supplementary text).

For each P' we designated these nodes to be the set of positive examples and executed each of the prediction algorithms, ensuring that the negative samples we selected for each P' did not intersect with the original set of positive examples P. Repeating this procedure 1,000 times, we constructed a distribution of scores for each node in P. We then estimated the p-value of a node's score as the fraction of values in this distribution that were at least as large as the score. We did not correct these scores for multiple hypothesis testing.

Analytical Perspective on the RL and Expected Path Length

As mentioned in the main text, the RL benefits from two complementary interpretations, both of which are important for our work. Here, we review those two interpretations. We then provide a new derivation that links these two interpretations, and in so doing provides justification for our specific setting of the α parameter of the RL. We use the same notation as we did to describe the steps of the RL algorithm.

First, the RL represents the optimal solution of a semi-supervised classification problem on networks [5], one that has a natural application in our setting. Consider a binary classification problem in which network nodes may have a positive label, a negative label, or be unlabeled. Associate with each node u in the network a value $y(u)$ which is 1 when the node has the positive label, and 0 otherwise (i.e., the node has the negative label or is unlabeled). This assignment reflects the choice that when facing unlabeled nodes, the labeling should be biased toward the negative label. We seek to form a new labeling $s(u)$ reflecting confidence that each node u in fact has the positive label. The estimate takes into account both the known labels and the assumption that adjacent nodes in the network should have similar labels. We formalize this goal via following expression:

$$\sum_{u\in V}(s(u)-y(u))^2+\alpha\sum_{(u,v)\in E}\tilde{w}_{uv}(s(u)-s(v))^2. \qquad (1)$$

This expression measures the amount to which the new labeling $\vec{s}$ differs from the input labeling $\vec{y}$ (first term) and the amount to which adjacent nodes have different labels (second term). We form the new labeling $\vec{s}$ by minimizing this expression over all vectors in $\mathbb{R}^n$. Seen in this light, the parameter α controls the tradeoff between these two opposing goals: a large α results in a labeling that changes smoothly between adjacent network nodes, whereas a small α results in sharper changes in $\vec{s}$ between adjacent nodes.

It can be shown that to find $\vec{s}$, it suffices to solve the system of linear equations $(I + \alpha\tilde{L})\vec{s} = \vec{y}$ [5], as we do in Step 6 of the RL algorithm above. For any connected graph G, this system always has a unique solution, and so $(I + \alpha\tilde{L})$ is always invertible.

Although this node-labeling interpretation of the RL has a natural application to our setting as a way of determining which proteins ($\vec{s}$) are most associated with SARS-CoV-2 interactors ($\vec{y}$), it leaves open the question of the best choice of α, i.e., how "smooth" should be the final labeling $\vec{s}$ on the network. Recall that the performance of the RL during cross validation did not show much variation with α (Parameter Selection and Figure S4). Hence, to address the choice of α, we turn to the second interpretation of the RL.

The second interpretation of the RL is in terms of a *continuous-time Markov chain* (CTMC) on the network [5]. Like a discrete-time Markov chain, a CTMC has the Markov property that future events are independent of past events, given the present state of the system. Since a CTMC is in continuous time, the time spent in each node is exponentially distributed. This property is a consequence of the fact that the exponential distribution is the only continuous distribution with the Markov (or memoryless) property. That is, for an exponential random variable X,

$$P[X > t + k \mid X > k] = P[X > t]. \qquad (2)$$

We can associate a random walk with the CTMC , as follows. Given the graph defined by $\tilde{W}$, the walker spends an exponentially distributed time in a node u with expected duration $1/\tilde{D}_{uu}$. After the exponentially distributed time, the walker moves to a new node v with probability $\tilde{w}_{vu}/\tilde{D}_{uu}$. Thus the transition probabilities of the walker are given by $\tilde{W}\tilde{D}^{-1}$.

Assume the walker starts in node u with probability $b(u)$. We are interested in the location of the walker after an elapsed time T, where T depends on α. To establish the connection between this CTMC and the RL, we choose T to be an exponentially distributed random variable with mean α, i.e., $P[T > x] = e^{-x/\alpha}$. Then it is possible to show that the probability $s(v)$ of finding the walker in a node v at time T is given as $\vec{s} = (I+\alpha\tilde{L})^{-1}\vec{b}$ [6]. Thus the RL can be interpreted as an amount of diffusion that flows in the network between any two nodes, when diffusion time is governed by α. In fact, there is an equivalent interpretation of the RL in terms of fluid flow [7, 8].

To decide how to set α in a way that balances smoothness of labeling s with known input labels, we propose to tune the random walk length to match typical path lengths in the networks under study. As described in the main text, we determined empirically that median path length from SARS-CoV-2 interactors to all other proteins was three. Hence we seek an α that yields comparable walk lengths.

To this end, we derive the expected length of the random walk that starts according to $b(u) = y(u)/\sum_v y(v)$. (As before, we set $y(u) = 1$ if u is a source node, and 0 otherwise.) We are interested in the number of steps taken by the random walk during the elapsed time T.

We denote by p_n the probability that the walker has made n transitions at time T, conditioned on the fact that the total time spent in transitions $0, 1, 2, \ldots, n-1$ is less than T. For example, p_0 is the probability that the walker has not made any transition before time T.

Consider the instant when the walker enters node u. Let $X(u)$

be the random variable denoting the sojourn time, i.e., the time elapsing from when the walker enters u until it moves to the next node. Hence $X(u)$ is an exponential random variable such that $P[X(u) > x] = e^{-\tilde{D}_{uu}x}$. Further, let R denote the time elapsing from when the walker enters u until we reach time T. By the memoryless property of the exponential distribution (equation 2), regardless of how long the walker has been walking, R is also exponentially distributed with mean α. Time T will be reached while the walker is in node u if $R < X(u)$. So the probability that time T is reached while the walker is in node u is $1/(\alpha\tilde{D}_{uu} + 1)$.[1] Hence

$$p_0 = \sum_{u \in G} b_u/(1 + \alpha\tilde{D}_{uu}) = \mathbf{1}^T(I + \alpha\tilde{D})^{-1}b$$

where **1** represents a vector of all ones.

If T is not reached while the walker is in node u, the walker goes on to a new node according to the transition probabilities $\tilde{W}\tilde{D}^{-1}$ and the process is repeated. Hence,

$$p_1 = \mathbf{1}^T(I + \alpha\tilde{D})^{-1}\tilde{W}\tilde{D}^{-1}b$$

and

$$p_n = \mathbf{1}^T(I + \alpha\tilde{D})^{-1}(\tilde{W}\tilde{D}^{-1})^n b$$

So the unconditional probability that time T is reached at step n is

$$p_n \prod_{i=0}^{n-1}(1 - p_i).$$

Let the random variable N denote the number of transitions taken by the walk before time T. Then

$$E[N] = \sum_{n=0}^{\infty} n p_n \prod_{i=0}^{n-1}(1 - p_i)$$

.

For a given starting distribution $\vec{b}$, we compute enough terms of these expressions until convergence to find the expected number of steps before time T is reached.

1 For exponential random variables X_1 and X_2 with parameters λ_1 and λ_2, $P[X_1 < X_2] = \frac{\lambda_1}{\lambda_1+\lambda_2}$.

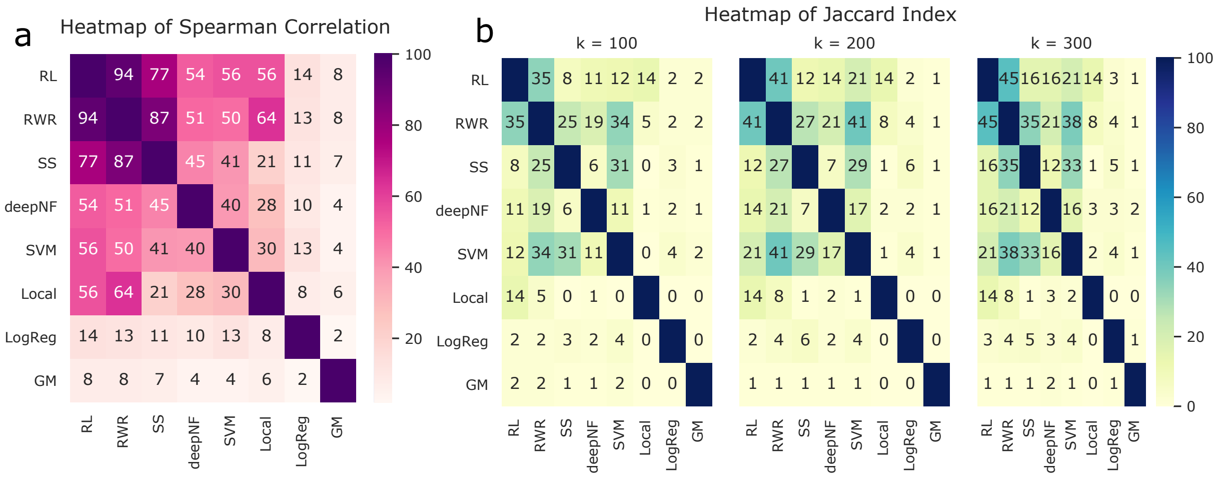

Figure S3. Similarity of predictions between every pair of methods. (**a**) Spearman correlations of node prediction scores. (**b**) Overlap of the top k predictions of each method, measured using the Jaccard index. The number in each cell is the value of the corresponding correlation or Jaccard index multiplied by 100.

Overlap Among Algorithms

To understand the diversity of the predictions across the prediction methods, between each pair of algorithms, we computed the Spearman's correlation of all scores. We also compared the top-ranking predictions between algorithms using the Jaccard index. RL had a high correlation with Local (0.75), which was surprising given the poor performance of Local. However, the Jaccard index for the two methods was around 0.1 (Figure S3(b)), suggesting that they shared very few top-ranking predictions and that the high correlation may be caused by lower-scoring proteins. SVM had moderate values of correlation and Jaccard's index with RL and SS (around 0.4 and 0.3, respectively). LogReg and GM both had low correlations and Jaccard's indices with all other methods (< 0.15).

Parameter Selection

Algorithms. To tune the methods, we varied the parameter α for RL, the weight of the edges λ connecting each node to the artificial sink for SS, and the parameter C controlling the inverse of the regularization strength for SVM and LogReg. For each setting of these parameters, we repeated 5-fold cross-validation with all three positive:negative ratios. We show the results for the ratios 1:1, 1:5, and 1:10 in Figure S4 and observed the results were fairly consistent across ratios. We focused on optimizing early precision values since we were interested in the analysis of top-ranking predictions.

For RL, GM, and SS, we found that in general, constraining the propagation locally around positive examples (i.e., small values of α, large λ) achieved higher early precision than more global propagation (i.e., large α, small λ). We chose the parameter values $\alpha = 0.01$, $\alpha = 0.1$, and $\lambda = 100$ for RL, GM, and SS, respectively.

For the supervised classifiers SVM and LogReg, we found that decreasing the regularization parameter (i.e., trying large values of C) resulted in a slight increase in median early precision (about 0.05 for SVM, and 0.07 for LogReg) over the default $C = 1$. However, to avoid overfitting, we chose to use $C = 1$ for both methods. For the SVM used by deepNF, we chose to use $C = 0.1$, which resulted in the best performance across the various ratios and measures.

Stratified sampling. The number b of bins is a parameter. We tested $b = 10, 20, 30$ and found that in each case, almost all of the top 332 nodes had a p-value < 0.05 with the exception of the top 15 predictions for SVM, many of which had p-values slightly higher than the cutoff (Figure S5). In general, the p-values for SVM were slightly higher than those for RL. Since we did not observe much difference when varying b, we selected $b = 10$.

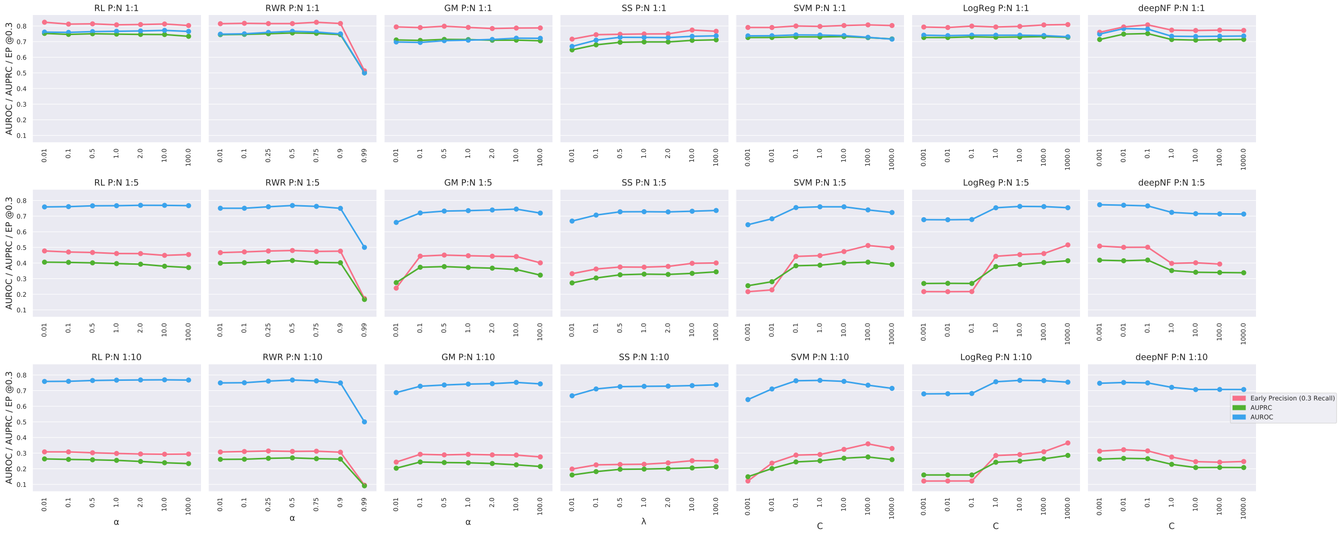

Figure S4. Parameter search results for each method, evaluated using AUROC, AUPRC, and early precision (at recall equal to 0.1) of 5-fold CV with a positive:negative ratio of 1:1, 1:5, and 1:10 on the STRING network. Each point shows the median value of 100 repetitions

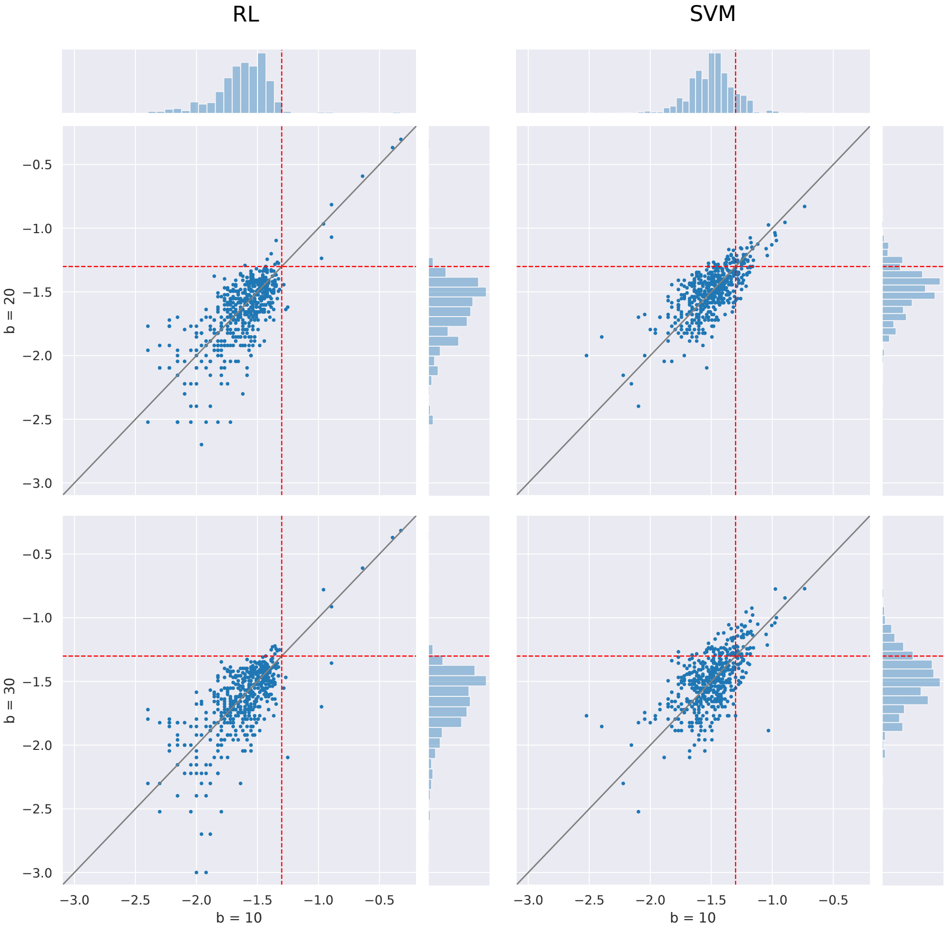

Figure S5. Base-10 logarithms of the p-values of node scores of the top 500 ranked proteins for RL and SVM for three values of b. The red dashed lines show the significance cutoff of 0.05, while the diagonal line shows $x = y$.

Functional Enrichment

We used the `clusterProfiler` package in *R* [9] to compute GO terms, Kyoto Encyclopedia of Genes and Genomes (KEGG) pathways, and Reactome pathways enriched in our predictions or in the human interactors of SARS-CoV-2 proteins. This package uses Fisher's exact test to estimate the enrichment of an individual term or pathway and the method of Benjamini and Hochberg to correct for testing multiple hypotheses. We applied this correction for each database (GO, KEGG, Reactome) separately. We used a threshold of 0.01 to decide if a GO term or KEGG/Reactome pathway was significantly enriched.

The enrichment analysis yields many highly similar statistically significant GO terms and KEGG and Reactome pathways; by "similar", we mean that two different terms or pathways may annotate many proteins in common. This problem is well-known with several approaches that have been proposed to mitigate it either by grouping similar terms and selecting a small subset of dissimilar terms [10, 11] or by directly computing a set of non-redundant GO terms [12, 13]. As far as we can tell, these methods have been developed to consider the enriched GO terms for one set of terms. When we apply them independently to different sets of proteins (e.g., predictions from RL and predictions from SVM), they may select one term for one set of proteins but a similar but not identical term for another set, making the distinctions in enrichment hard to discern.

Therefore, taking inspiration from previously developed methods (cited above), we developed a simple heuristic based on the weighted set cover algorithm that simultaneously simplifies multiple sets of enriched terms or pathways. For every term that is enriched in at least one protein set, we defined its *composite odds ratio* to be the product of the odds ratios for that term across the protein sets. We iteratively selected the term with the largest composite odds ratio, deleted the proteins annotated to this term from the annotations of all other enriched term, adjusted the odds ratio for every term, and recomputed the composite odds ratios. We stopped when the maximum composite odds ratio of the remaining terms became less than one. We demonstrate how this algorithm was successful in reducing the redundancy among enriched GO terms in "Evaluation of the Method for Simplifying Functional Enrichment Results" below.

In the case of two terms automatically selected by this algorithm, there was a different, highly overlapping term that we felt would be more interpretable in the context of SARS-CoV-2 and COVID-19. Therefore, we manually replaced the selected terms by their alternative choices. Specifically, we replaced "ciliary basal body-plasma membrane docking" and "mRNA transport" with "cilium assembly" and "viral transcription", respectively. To substantiate our choices, we computed the Jaccard index between the original and replaced term. Here we considered the proteins annotated by a term across all the protein sets (i.e., top-ranking proteins from RL and SVM and human interactors of SARS-CoV-2 proteins). The Jaccard index value was 0.95 between "ciliary basal body-plasma membrane docking" and "cilium assembly" with all 88 proteins annotated by the first term being annotated by the second, which had 93 annotations. The Jaccard index between "mRNA transport" and "viral transcription" was 0.46. The terms annotated 40 and 43 proteins, respectively, with 26 proteins in common. In addition, we removed many terms with a small number of annotations (≤ 7) that were fairly similar to other terms in the list. We used the final list of terms for further analysis.

Enriched Biological Processes

Since parent-child relationships in the GO cause many closely related terms to be enriched, we used a heuristic to select a non-redundant set of terms that were enriched in at least one of these sets of proteins ("Methods" and the section "Evaluation of the Method for Simplifying Functional Enrichment Results"). While some terms were common to all three sets of proteins, there were many that were enriched only in our predictions (Figure 2(b) in the main text, "Enrichment results for RL, SVM and viral interactors" [14]) indicating that network propagation successfully identified specific cellular processes involving proteins proximal to, but not directly interacting with, viral proteins. Figure S6 provides a high-level view of these GO terms and their connections in the STRING network to SARS-CoV-2 interactors and SARS-CoV-2 proteins. In the rest of this section, we examine the relevance of some of these processes to the viral cell cycle.

One group of enriched terms are related to protein translation (green nodes in Figure S6). These terms annotate only the SVM's top-ranking proteins (second group in Figure 2(b) in the main text. They include "tRNA export from nucleus"(p-value 3.35×10^{-5} for RL, p-value 7.15×10^{-21} for SVM, and p-value 1.2×10^{-4} for SARS-CoV-2 interactors) and"ribosomal large subunit biogenesis" (p-value 1.12×10^{-10} for SVM and 0.69 for SARS-CoV-2 interactors). RNA viruses appear to reduce protein synthesis in host cells, including in the case of SARS-CoV [15]. A recent proteomics study that infected Caco-2 cells with SARS-CoV-2 [16] revealed that while global translation rates in host cells exhibited only minor changes, the levels of a significant number of human proteins involved in translation were highly positively correlated with levels of key viral proteins. This study further showed that translation inhibitors prevented SARS-CoV-2 replication. The authors concluded that SARS-CoV-2 may increase production of proteins involved in the translation machinery components so as to compensate for inhibition of translation in host cells.

The GO term "viral transcription" is enriched in top-ranking SVM proteins (p-value 3.44×10^{-19}) but not in SARS-CoV-2 interactors (p-value 0.05). The proteins involved are primarily nucleoporins and members of the large ribosomal subunit. An overwhelming fraction of the annotations to this GO term come from the Reactome pathway "Viral Messenger RNA Synthesis" (R-HSA-168325). This pathway describes how the influenza A virus co-opts the host machinery to transcribe its mRNAs. Thus the enrichment of this GO term in the top-ranking SVM proteins may indicate a similar exploitation of the host by SARS-CoV-2.

N-linked glycosylation is an important post-translational protein modification that is conserved across eukaryortes [17]. It involves the attachment of an oligosaccharide to an asparagine residue of a protein [17]. The process takes place in two major stages. The first step involves the biosynthesis of lipid-linked oligosaccharides [17] corresponding to the GO term "oligosaccharide-lipid intermediate biosynthetic process" (Figure S7(a)), which is enriched in top-ranking RL proteins (p-value 2.98×10^{-8}) but not in SARS-CoV-2 interactors (p-value 0.35). In the second step, oligosaccharyltransferases enable the transfer of the oligosaccharide to the asparagine residue. These proteins are annotated to the GO term "protein N-linked glycosylation via asparagine", which is enriched in top-ranking RL proteins (p-value 2.2×10^{-6}) but not in SARS-CoV-2 interactors (p-value 0.2).

Several viruses can exploit the host glycosylation pathway to modify viral proteins in order to enter host cells and evade the immune response [18]. An extensive glycan shield that covers the S protein in human coronavirus NL63 (HCoV-NL63) masks the protein surface, thus limiting access to neutralizing antibodies [19]. The receptor-binding and fusion-peptide domains of the S protein (fusion protein) in SARS-CoV-2, SARS-CoV, and MERS-CoV contain N-glycans [20, 21]. It appears that the N-linked glycan modifications of SARS and MERS CoV S proteins are extensive but do not form as effective a shield as in viruses such as HIV-1 [20]. Drugs that target host N-linked glycosylation pathways have been proposed as treatments for COVID-19 [22].

The term "(GPI)-anchor biosynthetic process" is significantly enriched in the top-ranking proteins from RL (p-value 1.4×10^{-6}) but not in the human interactors of SARS-CoV-2 (p-value 0.21). The proteins prioritized by our methods (Figure S7(b)) are either components of the Gycosylphosphatidylinositol (GPI) transamidase complex or transfer GPI to proteins during the synthesis of GPI anchors; these anchors tether proteins to lipid bilayers [23]. GPI-anchored proteins are often associated with lipid rafts, which are microdomains in plasma membranes that are enriched with cholesterol and sphingolipids [23]. Lipid rafts play a major role in viral entry, assembly, replication, and budding. They are known to be involved in the entry of SARS-CoV into host cells [24]. Thus, the proteins involved in (GPI)-anchor biosynthesis that we prioritize may provide a deeper understanding on SARS-CoV-2 entry into host cells.

Several GO terms related to cellular respiration (blue nodes in Figure S6) were enriched in the top-ranking proteins, e.g., "protein import into mitochondrial matrix" (Figure S7(d); p-value of 4.11×10^{-12} for RL and 0.3 for SARS-CoV-2 interactors). Many of the proteins annotated to this term are translocases of the inner and outer membranes of the mitochondrion. These proteins comprise complexes that transport proteins across the mitochondrial membranes. "NADH dehydrogenase complex assembly" is a related GO biological process (Figure S7(c)), which has a p-value of 3.55×10^{-19} for RL in contrast to a p-value of 0.035 in SARS-CoV-2 interactors. NADH dehydrogenase is the first complex in the mitochondrial respiratory chain. Viral infections have been proposed to cause the Warburg effect that is frequently associated with cancer [25]. Due to oxygen deprivation, cells switch from respiration to glycolysis. We note that a recent genome-wide CRISPR screen for human genes that regulate SARS-CoV-2 infection [26] identified several members of the respiratory chain as "anti-viral", i.e., the knockout of the gene sensitizes the host cell to virus-induced cell death. Thus, the GO terms enriched in our results may suggest mechanisms by which SARS-CoV-2 modulates cellular respiration in the host to promote infection.

The GO biological process "protein alpha-1,2-demannosylation", i.e., the removal of a mannose group from a protein, is enriched in top-ranking RL proteins (p-value 2.98×10^{-8}) but not in SARS-CoV-2 interactors (p-value 0.09). Key top-ranking proteins in this network (Figure S7(e)) include MAN1B1 and UGGT1, which are localized to the ER. Enveloped viruses have been shown to hijack the host cell's ER for survival and replication, thereby causing ER-associated degradation (ERAD) [27]. MAN1B1 plays a role in protein quality control as well as in their degradation [28]. The utilization of UGGT1 by enteroviruses leads to increased viral replication and higher pathogenicity [29]. Another high-ranking protein EDEM1 controls the degradation of mis-folded glycoproteins and is a key player in ERAD [30] When EDEM1 is cleared from the ER, it is transported out of the organelle in nanoscale vesicles that are called EDEMosmomes. Coronaviruses such as the mouse hepatitis virus subsequently hijack these organelles and remodel them to form double membrane vesicles (DMVs) [31]. DMVs are

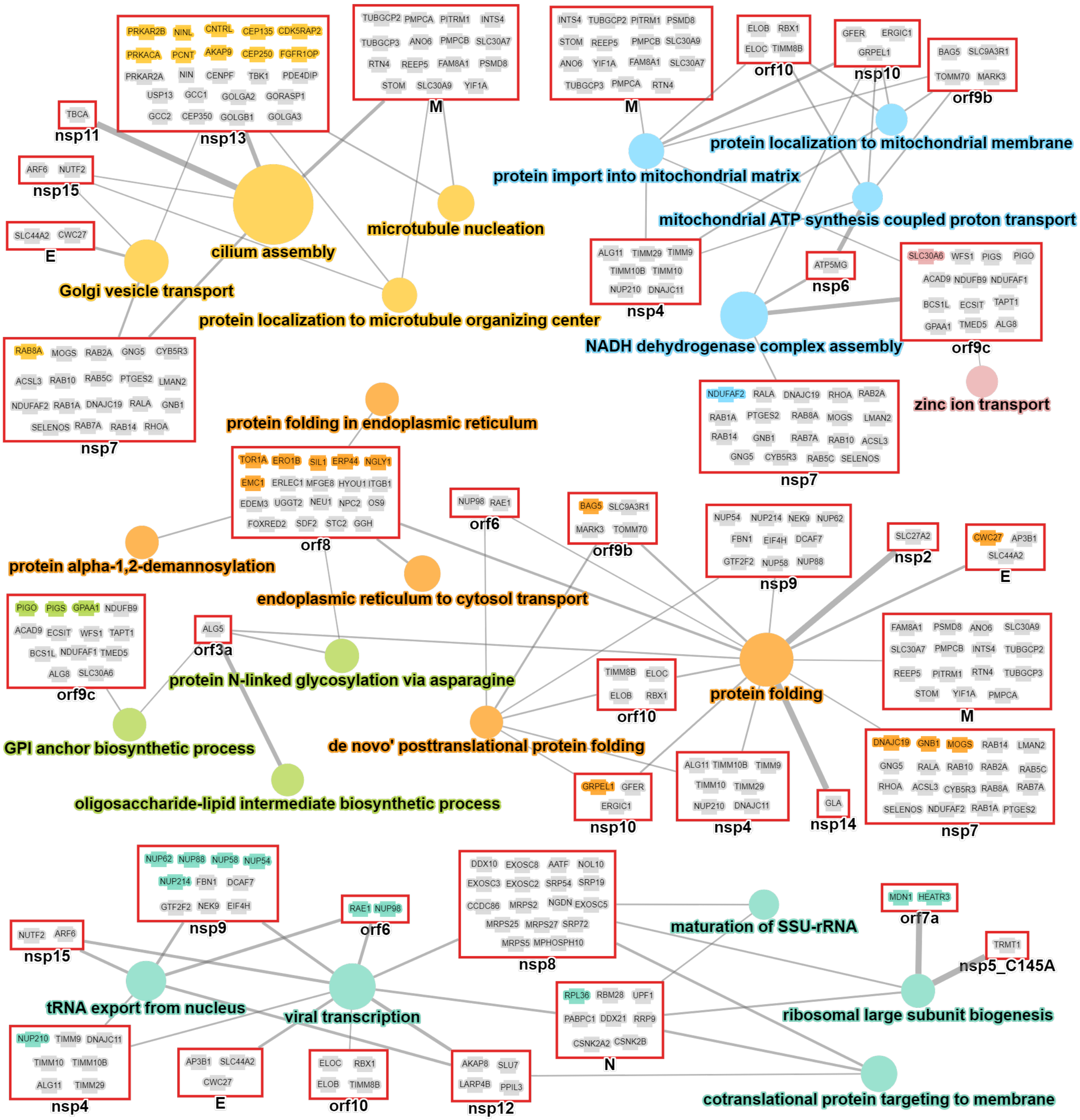

Figure S6. Overview of connections between SARS-CoV-2 interactors and the GO biological process terms enriched in the top-ranking proteins. Each circle is a GO term shown in Figure 2(b) in the main text; the color of the GO term corresponds to its group in this figure and its size corresponds to the number of annotated top-ranking proteins. Each box with a red border is a SARS-CoV-2 protein (the box labels). The box contains one or more human proteins that interact with that viral protein. A viral protein may label more than one box. A gray edge connects a red box to a GO term. The width of the edge is proportional to the fraction of the human interactors of the viral protein that are connected in the STRING network to the top-ranking proteins annotated to the term (considering only STRING edge weights ≥ 900). We do not show edges with a fraction < 0.05, except for the edge between orf9c and "zinc ion transport" (0.026). If a viral interactor is annotated to a GO term, its color is the same as that of the GO term.

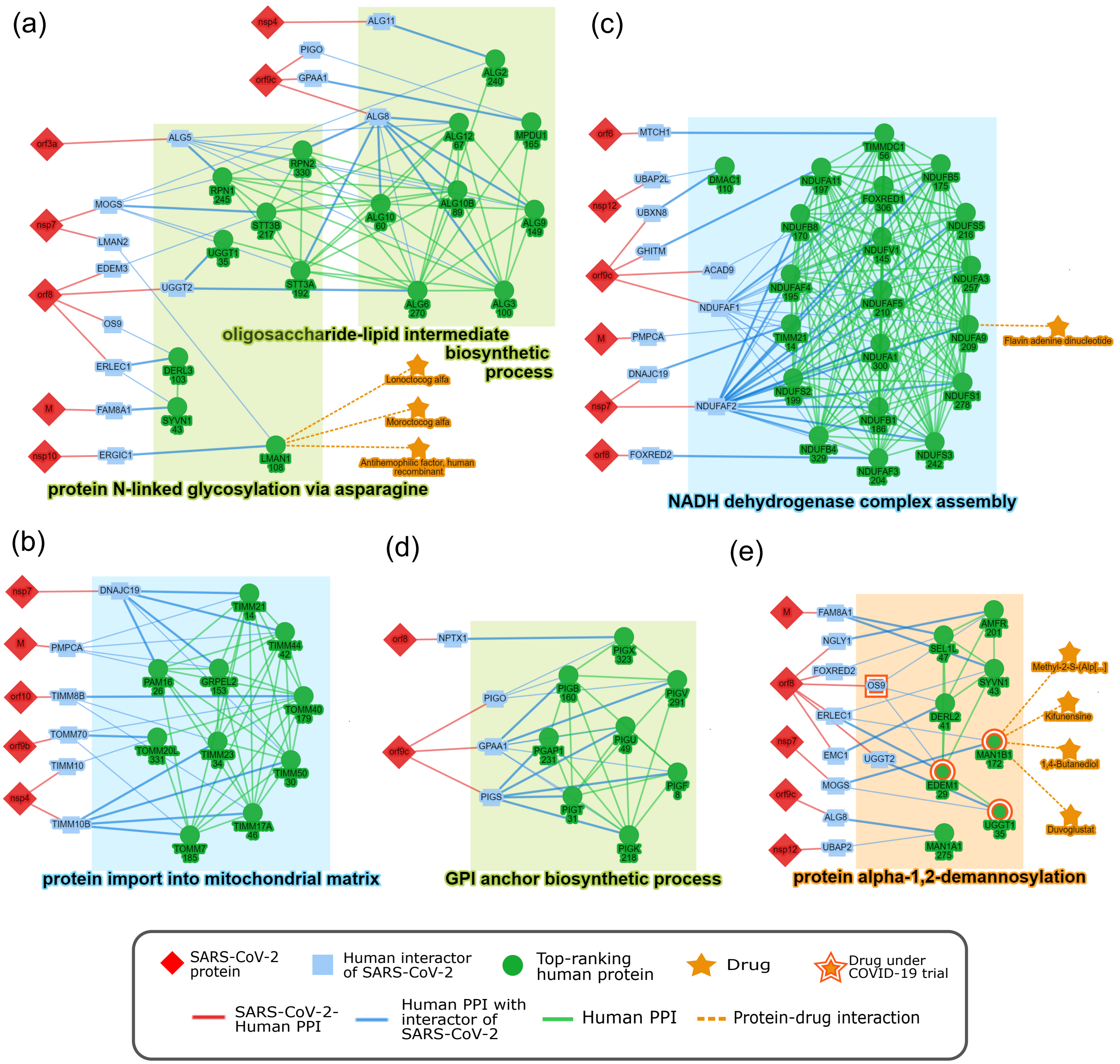

Figure S7. Networks of the top-ranking proteins for RL (green nodes) that are annotated tothe enriched terms (a) "oligosaccharide-lipid intermediate biological process" and "protein N-linked glycosylation via asparagine," (b) "GPI anchor biosynthetic process," (c) "NADH dehydrogenase complex assembly," (d) "protein import into mitochondrial matrix," or (e) "protein alpha-1,2-demannosylation." The number below the name of a green protein is its rank computed by the RL. A rectangle encompasses proteins (top-ranking or SARS-CoV-2 interactors) annotated to the respective term. Proteins discussed in the text are highlighted with a red border. For each top-ranking protein, we also display its connections with neighboring SARS-CoV-2 interactors. We removed STRING edges with weight < 700 to simplify the visualization.

widely used by viruses, including coronaviruses, to promote viral replication [32].

Evaluation of the Method for Simplifying Functional Enrichment Results

The main goal of simplifying the result from enrichment analysis was to filter out the redundant terms, i.e., terms that have highly overlapping annotated proteins. To evaluate how our simplification method ("Methods") performs in terms of reducing overlap , we analyzed the enrichment results from each protein set (i.e., top-ranking proteins from RL, top-ranking proteins from SVM, and human interactors of SARS-CoV-2 proteins) in two ways. We performed each analysis for the set of terms before simplification and for the set of terms after simplification. We show the results RL as an exemplar.

First, for each enriched term t, we computed the largest Jaccard index between t and every other enriched terms. We plot-

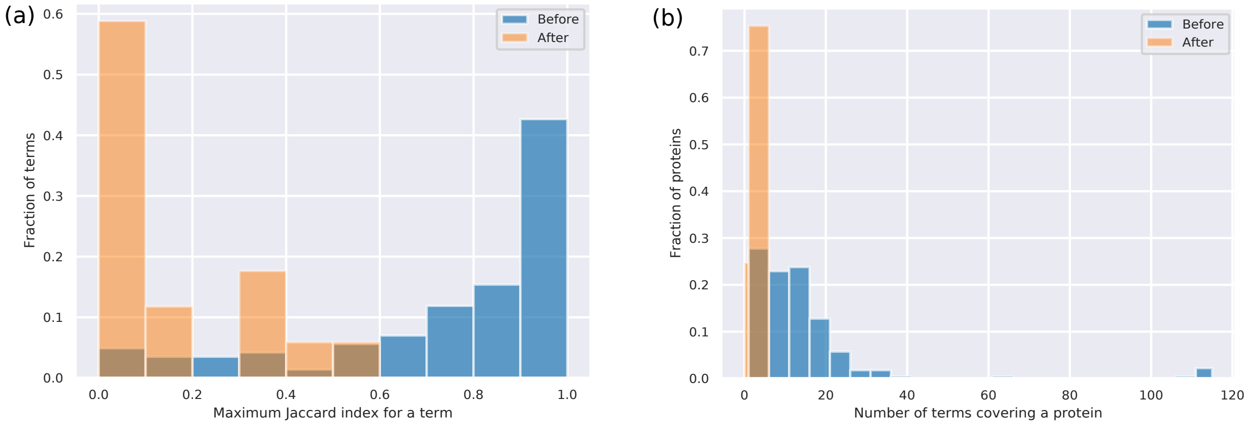

Figure S8. Results of simplification of enriched terms. (a) Distribution of the maximum Jaccard index values for the enriched GO biological processes in the top-ranking RL proteins, both before and after simplification. The x-axis corresponds to the maximum Jaccard index and the y-axis to the fraction of terms that have a maximum Jaccard index in a particular range. (b) Distribution of the number of enriched enriched GO biological processes containing each protein, considering the top-ranking RL proteins, both before and after simplification. The thin bar at 0 indicates the fraction of proteins in the protein universe left uncovered after simplification.

ted the distributions of these quantities. We expected the Jaccard indices to be large in the 'before' set and small in the 'after' set of terms. Indeed, we observed that our results exhibited these trends for each protein set. We present the result from top-ranking RL proteins in Figure S8(a).

For the top-ranking RL proteins, there were 144 and 18 enriched terms before and after simplification respectively. The maximum Jaccard index was ≥ 0.5 for 82% of the terms before simplification. This percentage reduced to 5.8% of the terms after simplification. Moreover, after simplification, for 88% of the terms, the maximum Jaccard index was < 0.4, which indicates a considerable improvement in overlap.

Second, we considered the set of proteins annotated by at least one enriched term before simplification. There were 227 such proteins from the top-ranking RL results and GO biological processes. For each such protein, we computed the number of enriched terms that contained the protein and plotted the distributions of these counts. With the same set of proteins, we repeated the analysis after simplification. We observed that before simplification, 72% of proteins were annotated by more than 5 enriched terms. This percentage reduced to zero after simplification. Moreover, the percentage of proteins annotated by five or fewer terms increased dramatically after simplification. We observed that before simplification 6.6%, 8.8%, 6.1%, 2.2%, and 3.9% of proteins were covered by 1, 2, 3, 4, and 5 terms, respectively. In contrast, these values were 55.1%, 16.3%, 3.5%, 0.4%, and 0% after simplification. In addition, 24.6% of proteins were not covered by any terms after simplification. We recomputed the GO terms enriched in thee proteins. These terms were closely related to glycosylation, a process which we have already found to be relevant in the context of SARS-CoV-2 in our list of simplified enriched terms.

Overall, these results confirmed the efficacy of our approach in computing non-redundant enriched terms.

References

1. Mostafavi S, Ray D, Warde-Farley D, Grouios C, Morris Q. GeneMANIA: a Real-Time Multiple Association Network Integration Algorithm for Predicting Gene Function. Genome Biology 2008;9(Suppl 1):S4. http://dx.doi.org/10.1186/gb-2008-9-s1-s4.
2. Zhu X, Ghahramani Z, Lafferty JD. Semi-supervised learning using Gaussian fields and harmonic functions. In: The Twentieth International Conference on Machine Learning, August 21-24, 2003, Washington, DC USA; 2003. p. 912–919.
3. Murali TM, Dyer MD, Badger D, Tyler BM, Katze MG. Network-based prediction and analysis of HIV dependency factors. PLoS computational biology 2011 9;7(9):e1002164+. http://dx.doi.org/10.1371/journal.pcbi.1002164.
4. Gligorijević V, Barot M, Bonneau R. deepNF: Deep Network Fusion for Protein Function Prediction. Bioinformatics 2018;34(22):3873–3881. https://doi.org/10.1093/bioinformatics/bty440.
5. Fouss F, Francoisse K, Yen L, Pirotte A, Saerens M. An experimental investigation of kernels on graphs for collaborative recommendation and semisupervised classification. Neural Networks 2012;31:53 – 72. http://www.sciencedirect.com/science/article/pii/S0893608012000822.
6. Avrachenkov K, Chebotarev P, Mishenin A. Semi-supervised learning with regularized Laplacian. Optimization Methods and Software 2017;32(2):222–236. https://doi.org/10.1080/10556788.2016.1193176.
7. Qi Y, Suhail Y, Lin Yy, Boeke JD, Bader JS. Finding friends and enemies in an enemies-only network: A graph diffusion kernel for predicting novel genetic interactions and co-complex membership from yeast genetic interactions. Genome Research 2008;18(12):1991–2004. http://genome.cshlp.org/content/18/12/1991.abstract.
8. Vandin F, Upfal E, Raphael BJ. Algorithms for detecting significantly mutated pathways in cancer. J Comput Biol 2011 Mar;18(3):507–522.
9. Yu G, Wang LG, Han Y, He QY. ClusterProfiler: An R pack-

age for comparing biological themes among gene clusters. OMICS A Journal of Integrative Biology 2012;16(5):284–287.
10. Supek, F and Bošnjak, M and Škunca, N and Tomislav, Š . REVIGO Summarizes and Visualizes Long Lists of Gene Ontology Terms. PLoS One 2011 July;6(7):e21800.
11. Merico D, Isserlin R, Stueker O, Emili A, Bader GD. Enrichment map: a network-based method for gene-set enrichment visualization and interpretation. PLoS ONE 2010 Nov;5(11):e13984.
12. Lu Y, Rosenfeld R, Simon I, Nau GJ, Bar-Joseph Z. A probabilistic generative model for GO enrichment analysis. Nucl Acids Res 2008 Oct;36(17):e109+.
13. Bauer S, Gagneur J, Robinson PN. GOing Bayesian: model-based gene set analysis of genome-scale data. Nucleic Acids Research 2010;38(11):3523–3532.
14. Law J, Akers K, Tasnina N, Della-Santina C, Deutsch S, Kshirsagar M, et al., Supporting data for "Interpretable Network Propagation with Application to Expanding the Repertoire of Human Proteins that Interact with SARS-CoV-2". GigaScience Database; 2021. http://dx.doi.org/10.5524/100941.
15. Kamitani W, Huang C, Narayanan K, Lokugamage KG, Makino S. A two-pronged strategy to suppress host protein synthesis by SARS coronavirus Nsp1 protein. Nat Struct Mol Biol 2009 Nov;16(11):1134–1140.
16. Bojkova D, Klann K, Koch B, Widera M, Krause D, Ciesek S, et al. Proteomics of SARS-CoV-2-infected host cells reveals therapy targets. Nature 2020 May;.
17. Breitling J, Aebi M. N-linked protein glycosylation in the endoplasmic reticulum. Cold Spring Harb Perspect Biol 2013 Aug;5(8):a013359.
18. Vigerust DJ, Shepherd VL. Virus glycosylation: role in virulence and immune interactions. Trends Microbiol 2007 May;15(5):211–218.
19. Walls AC, Tortorici MA, Frenz B, Snijder J, Li W, Rey FA, et al. Glycan shield and epitope masking of a coronavirus spike protein observed by cryo-electron microscopy. Nat Struct Mol Biol 2016 Oct;23(10):899–905.
20. Watanabe Y, Berndsen ZT, Raghwani J, Seabright GE, Allen JD, Pybus OG, et al. Vulnerabilities in coronavirus glycan shields despite extensive glycosylation. Nat Commun 2020 May;11(1):2688.
21. Watanabe Y, Allen JD, Wrapp D, McLellan JS, Crispin M. Site-specific analysis of the SARS-CoV-2 glycan shield. bioRxiv 2020;https://www.biorxiv.org/content/early/2020/03/28/2020.03.26.010322.
22. Williams SJ, Goddard-Borger ED. α-glucosidase inhibitors as host-directed antiviral agents with potential for the treatment of COVID-19. Biochem Soc Trans 2020 Jun;.
23. Metzner C, Salmons B, Günzburg WH, Dangerfield JA. Rafts, anchors and viruses–a role for glycosylphosphatidylinositol anchored proteins in the modification of enveloped viruses and viral vectors. Virology 2008 Dec;382(2):125–131.
24. Lu Y, Liu DX, Tam JP. Lipid rafts are involved in SARS-CoV entry into Vero E6 cells. Biochem Biophys Res Commun 2008 May;369(2):344–349.
25. Sanchez EL, Lagunoff M. Viral activation of cellular metabolism. Virology 2015 May;479-480:609–618.
26. Wei J, Alfajaro MM, Hanna RE, DeWeirdt PC, Strine MS, Lu-Culligan WJ, et al. Genome-wide CRISPR screen reveals host genes that regulate SARS-CoV-2 infection. bioRxiv 2020;https://www.biorxiv.org/content/early/2020/06/17/2020.06.16.155101.
27. Bettigole SE, Glimcher LH. Endoplasmic reticulum stress in immunity. Annu Rev Immunol 2015;33:107–138.
28. Wu Y, Swulius MT, Moremen KW, Sifers RN. Elucidation of the molecular logic by which misfolded alpha 1-antitrypsin is preferentially selected for degradation. Proc Natl Acad Sci USA 2003 Jul;100(14):8229–8234.
29. Huang PN, Jheng JR, Arnold JJ, Wang JR, Cameron CE, Shih SR. UGGT1 enhances enterovirus 71 pathogenicity by promoting viral RNA synthesis and viral replication. PLoS Pathog 2017 May;13(5):e1006375.
30. Yoshida Y, Tanaka K. Lectin-like ERAD players in ER and cytosol. Biochim Biophys Acta 2010 Feb;1800(2):172–180.
31. Reggiori F, Monastyrska I, Verheije MH, Calì T, Ulasli M, Bianchi S, et al. Coronaviruses Hijack the LC3-I-positive EDEMosomes, ER-derived vesicles exporting short-lived ERAD regulators, for replication. Cell Host Microbe 2010 Jun;7(6):500–508.
32. Wolff G, Melia CE, Snijder EJ, Bárcena M. Double-Membrane Vesicles as Platforms for Viral Replication. Trends Microbiol 2020 Jun;.
