# Supplementary material for: Interpretable network propagation with application to expanding the repertoire of human proteins that interact with SARS-CoV-2
Source: Gigascience. 2021 Dec 29;10(12):giab082. doi: 10.1093/gigascience/giab082 (PMC8716363; doi:10.1093/gigascience/giab082)
Supplement: giab082_Response_to_Reviewer_Comments_Original_Submission [file giab082_response_to_reviewer_comments_original_submission.pdf]

Author's Response To Reviewer Comments

Close

Response to Reviewers
Interpretable Network Propagation with Application to Expanding the Repertoire of Human Proteins that Interact with SARS-CoV-2

We thank the reviewers for their insightful comments and suggestions. We made a few changes to the manuscript and added several analyses to the supplement. We highlight these changes in the manuscript using a different color.

The changes in the main manuscript include restructuring “Analysis” to give more importance to our main contributions on provenance tracing:
1. We shortened the subsection “Prioritization of Potential SARS-CoV-2 Interactors” by moving the cross validation results to the supplementary results. Consequently, this subsection contains the evaluations of predictions made by RL in terms of overlap with other datasets and functional enrichment. We retained these analyses in the main text since they confirm that our predictions are biologically meaningful and relevant to the virus.
2. We reordered the section “Tracing the Provenance of Top-Ranking Proteins” by moving the analysis of alpha below the main results for our provenance tracing approach.

The analyses we added are as follows:
1. Results for Random Walk with Restarts (RWR) in the sections on cross-validation (CV), parameter selection, and overlap among algorithms.
2. Comparison to PPI networks (BioGRID, BioGRID-Y2H, HI-union) for the effective diffusion results.
3. Overlap of our top-ranking proteins with two more experimentally determined sets
a. Differential protein abundance in SARS-CoV-2–infected iAT2 cells.
b. Differential gene expression in upper airway samples in SARS– CoV-2–infected patients

Finally, we divided the Supplementary Information into three parts: Supplementary Methods, Supplementary Results, and Supplementary Figures.

We address each reviewer’s comments in detail below.

Reviewer 1
The article provides information, useful and verifiable; presenting an adequate methodological approach applied to the study of the most important public health problem that now urges us. However, unlike what the authors argue in some sections, I do not consider it very original, as it is only a refinement of what has been done in propagation studies in networks for some time, but without a doubt its results are valuable, which more than justifies its publication. On the other hand, the mathematical description of the algorithm is quite clear, much clearer than what is described in the text of the document, which I suggest to review, as it is difficult in some sections.

We appreciate the reviewer’s viewpoint. We hope that the reviewer finds that our restructuring of the text and edits have improved the clarity of the presentation.

Reviewer 2
The authors designed a network propagation framework with two novel components and applied it to predict human proteins that directly or indirectly interact with SARS-CoV-2 proteins. There are several major problems:
1. There have been several similar studies which identified covid-19 infection-related human genes based on a random walk model in a virus-human protein interaction network (PMID: 32685484). The authors need to compare with previous works.

Thank you for this suggestion. Since the cited manuscript uses RWR as the primary algorithmic

component, we also executed RWR using the same source nodes (i.e., human proteins that interact with SARS-CoV-2 proteins), and found that it produced similar results to RL. We added a citation to this specific paper suggested by the reviewer in a paragraph on related work in “Discussion”.

2. The authors used network-based method to predict SARS-CoV-2 interaction protein. But there is plenty of gene expression data. The authors should compare the network genes with expression genes, such as PMID: 33505977 and 33519902.

We appreciate the recommendation to compare network-based predictions with differential gene expression data. We have added a paragraph to “Analyses” discussing the overlap between our top-ranking predictions and experimental data sets identifying differential protein abundance and gene expression in response to SARS-CoV-2 infection. We have updated Figure 2(a) with the results for protein abundance and added Supplementary Figure S8 for the overlap with differential gene expression data. We have also cited the relevant papers that published the original experimental data.

3. Within the network methods, there are network embedding (PMID: 31455874), network diffusion (PMID: 30069494), random walk with restart (PMID: 28787010, 28932904), shortest path (PMID: 28076954, 27434024, 26209080, 28974058, 23762832, 24056857, 22496748) and guilt-by-association (doi: 10.1016/j.neucom.2015.09.136). These methods should be introduced.

Thank you for this idea. Please note that the first seven papers that we have already cited in our manuscript are among the earliest in the field of network biology (published between 2003 and 2008) that use ideas in network diffusion, random walks, and guilt by association. We also cite an early review in the field that covers these topics, including shortest paths. In addition, the first paper we cite in “Analyses” is a more recent review that covers the topics mentioned by the reviewer. Our perspective is that it is important to cite such papers that may have influenced the field.

In this work, we compared a specific, representative set of network methods, including network embedding (deepNF), network diffusion (RL, GeneMANIA, and RWR; note that RWR is in fact a network diffusion method), and guilt-by-association (Local). We chose not to compare with shortest path approaches since network propagation methods implicitly consider all paths in the network, with parameters (e.g., the restart parameter for RWR) controlling for the relative importance of short vs long paths.

4. The supplementary materials are different from the descriptions of File S1 … Figure S4. Please check them carefully.

Thank you for pointing out this error. We have checked and corrected the supplementary files. Please note that there are only three files now.

Reviewer 3
In their manuscript, Law and colleagues use network propagation to predict potential SARS-CoV-2-interacting human proteins. On the methods front, they propose a means to (1) track down the prioritization contributions of individual seed proteins experimentally documented to interact with the virus; (2) select the main parameter of their network propagation method of choice (regularized Laplacian - RL) in an unbiased manner by relating it to the expected shortest path length of the random walk.

Overall, the individual sections of the paper are clear and well-written. However, taken together, the two main analyses somewhat lack a unifying narrative and feel disconnected from each other. While the methodological contributions of the paper seem interesting, the core choice of methods in the application to SARS-CoV-2 seems inadequately justified. Below are my two major concerns pertaining to each part of the analysis.

We very much appreciate the reviewer’s careful inspection of our work and considerable effort put into their review. We hope that the substantial restructuring of the manuscript and additional analyses and rationale we have provided address the concerns raised by the reviewer.

Major comments
1) In general, in the first (prioritization) part of the analysis, it is not clear to me what the actual goal is. RL is benchmarked against several types of network propagation-based and "traditional" prioritization

approaches, then SVM is taken as a representative of the latter group. Proteins are then prioritized between these two methods and validated in silico by their overlap with literature and their functional annotations. As it stands, the point of discussing and comparing different methods is not clear. What makes RL stand out is not directly evident the way these results are presented. In fact, the entire first part of the results, in my view, does not make a sufficient case for the use of network propagation as the prioritization method of choice. It rather looks like other off-the-shelf methods perform comparably (Fig. 2a), and yield similar biological insights (Fig 2c). It seems to me that if comparison of methods was performed, the authors should at least focus on the unique insights provided by RL (e.g. in terms of GO terms), if any, compared to SVM.

We acknowledge the reviewer's point. Our main goal was to apply the RL and the provenance tracing method we developed to predict potential interactors of SARS-CoV-2. Rather than use RL directly, we first compared it to many different approaches to solve the same problem. Based on the reviewer's comments, we have statistically compared the distributions of AUROC, AUPRC, and precision at 0.3 recall of all pairs of algorithms in "Comparison of Cross-Validation Results" in the Supplementary Results. RWR and deepNF had slightly higher AUPRC and precision at 0.3 recall than RL for the 1:5 and 1:10 positive:negative ratios but not for the 1:1 ratio. Nevertheless, we selected RL (and SVM) for subsequent analyses as we explain in the main manuscript:

"To decide which methods to select for subsequent analyses,we compared them using 5-fold cross validation ("Comparison of Cross-Validation Results" in the supplementary text and Figure S1). RL, random walk with restarts (RWR) [23], and deepNF [26] had the highest values of area under the precision-recall curve followed by SVM and logistic regression. RL achieved marginally worse values of area under the precision-recall curve than RWR and deepNF. We selected one network propagation method (RL) and one supervised classifier (SVM) for the following reasons. We preferred RL over deepNF because the provenance tracing method we developed for RL enabled its results to be more easily interpreted than those for deepNF. Since RL and RWR produced highly similar predictions with a very high Spearman's correlation for the ranking of all proteins ("Overlap among algorithms" in the supplementary text and Figure S2), we selected RL as representative of the two methods. We chose SVM among the two off-the-shelf classifiers since it also had very good performance in cross-validation."

This reasoning also addresses the question that the reviewer raises about the choice of RL in the next comment. We acknowledge that using RWR instead of RL may have yielded similar results for provenance tracing. We intend to perform this analysis in a subsequent project.

2) Provenance tracing explored in the second part is very interesting as a premise, and the fact that it is relatively uninvestigated makes it an appealing topic. As the authors note, it is based on a simple principle: once the regularized Laplacian kernel is calculated, the values of the kernel matrix are row-sorted for each protein. While this is still, in a sense, a steady-state view, and the name "provenance tracing" inspires in the reader's mind a sequence of connections between seeds and targets, I think the authors did a good job showcasing how it simplifies the subnetworks related to each biological process of interest, improving interpretability. I am convinced by its utility in that sense. What I have been having trouble convincing myself about is the particular choice of RL, some claims around the novelty of the analytical results, and why \alpha matters in the first place. To elaborate:

2a) The rationale presented for putting emphasis on \alpha seems not so well-justified to me. The authors state that they looked for a different way to determine alpha than looking at AUROC/AUPRC values since they varied little over a wide range of alpha. Does this not mean that the choice of alpha does not impact the prediction results in the case of RL? I guess I don't fully understand the point of the derivation other than a purely mathematical exercise (whose precedents seem to exist - see below). This also bears asking why RL was chosen in the first place. It sounds in the paper as if RL is the only choice of kernel that can be utilized this way to compute the contribution of the seed nodes, whereas many other types of kernels should work in a similar manner (see Fouss et al. cited by the authors as [18]). It seems that for provenance tracing, any type of kernel could work as the authors simply use the values corresponding to the seeds at the steady state kernel matrix to represent the contribution of each seed on the candidate protein's score. For example, RL is indeed similar to RWR, a widely used propagation method in biology (see Köhler, Sebastian, et al. "Walking the interactome for prioritization of candidate disease genes." The American Journal of Human Genetics 82.4 (2008): 949-958), in terms of the form of its kernel. deepNF, one of the methods considered in the benchmark that does comparably or better than RL, is also based on RWR. Could the authors comment on the above points?

We agree with the reviewer that the choice of alpha does not impact the predictions in the case of RL. It

was important for us to have tested different values of alpha before reaching this conclusion. Although varying alpha did not have a major effect on the predictions for RL in this particular application, it may have a larger impact for other prediction problems.

We also agree that the RL is not the only kernel to which this type of provenance tracing is applicable. We mention this point at two places in the manuscript:
(i) Abstract: “We examine how our provenance tracing method can be generalized to a broad class of network-based algorithms.”
(ii) Discussion: “Our strategy for tracing provenance extends to any algorithm that makes predictions using a linear combination of evidence such as logistic regression and GeneMania [61]. In particular, it is applicable to the large number of random-walk-based methods that have been developed for predicting disease genes or annotations to GO terms [62, 63, 64, 65].”

Although deepNF is based on RWR, it uses an autoencoder to learn a low-dimensional embedding of the input network and an SVM with an RBF kernel trained on this embedding to solve the node labeling problem. Developing a provenance tracing strategy for deepNF is non-trivial and will require a substantial research effort.

2b) The authors take a mean-field approach relating the teleportation/damping parameter (\alpha) to the expected value of path lengths given a network. The authors then use the actual network to determine the median path length between seeds and candidates and find the \alpha that corresponds to this value from their precomputed lookup table. In a way, we can see this parameter selection process as the tuning of the random walk according to the network at hand. Regarding the analytical results on average path lengths in random walks/diffusion processes, similar results seem to exist:
Yazdani, Majid, and Andrei Popescu-Belis. "A random walk framework to compute textual semantic similarity: a unified model for three benchmark tasks." 2010 IEEE Fourth International Conference on Semantic Computing. IEEE, 2010.
Ghosh, Rumi, et al. "Non-conservative diffusion and its application to social network analysis." arXiv preprint arXiv:1102.4639 (2011).
Ghosh, Rumi, and Kristina Lerman. "Rethinking centrality: the role of dynamical processes in social network analysis." arXiv preprint arXiv:1209.4616 (2012).
Stojmirović, Aleksandar, and Yi-Kuo Yu. "Information flow in interaction networks." Journal of Computational Biology 14.8 (2007): 1115-1143.
Masuda, Naoki, Mason A. Porter, and Renaud Lambiotte. "Random walks and diffusion on networks." Physics reports 716 (2017): 1-58.
Could the authors elaborate on the difference of their approach and scope?

We appreciate the referee’s attention to this aspect of our work, which encourages us to expand on the motivation and novelty of our analysis.

The use of diffusion and random walks (broadly, “stochastic processes”) as tools in network analysis is extensive. However, a dimension that is not always emphasized in such analyses is the distinction between processes that evolve in discrete time (eg, “random walks” that evolve in “steps” or “iterations”) versus those that evolve in continuous time (eg, “diffusion” that evolves via “fluid flow”). These two kinds of processes require different analytic tools. Discrete-time processes (as exemplified by PageRank and random-walk-with-restarts) are more common, and their analysis is often simpler. However, the Regularized Laplacian (RL) that we rely on corresponds to the solution of a continuous-time process (eg, it can be described in terms of a fluid flow). The tools for analyzing such processes are based on continuous-time Markov chains (CTMCs), ie, systems of differential equations.

As noted by the referee, our work tunes the mean path length of a time-bounded continuous time random walk to match average path lengths in biological processes. To do so, the analytic challenge we overcome comes from the fact that path lengths take on discrete values, while the random walk evolves in continuous time, and the time bound is a random variable.

We note that even specialized references do not contain discussion of expected path lengths in the CTMC corresponding to a Regularized Laplacian. For example, the question is not discussed in the recent, comprehensive text “Algorithms and Models for Network and Link Analysis,” (Fouss et al). Before writing up our analysis, we searched the literature and contacted experts in the field of stochastic processes to discern if this problem had previously been solved. We found no evidence that it had been addressed before.

With respect to the papers pointed to by the referee, each does indeed contain consideration of path lengths, but most are concerned only with models based on discrete time. None of the papers considers our question specifically. Thus the results of those papers cannot be used to answer the question we address.

Specifically:
Yazdani, Majid, and Andrei Popescu-Belis. "A random walk framework to compute textual semantic similarity: a unified model for three benchmark tasks." 2010 IEEE Fourth International Conference on Semantic Computing. IEEE, 2010.

This paper uses "hitting time" and "commute time" as measures of walk length. Although these terms involve the word “time”, here time is understood to be a discrete quantity. For example, hitting time (i,j) is the mean number of steps in a discrete time random walk starting at i, up to the first visit to node j.

Ghosh, Rumi, et al. "Non-conservative diffusion and its application to social network analysis." arXiv preprint arXiv:1102.4639 (2011).

This paper studies "non-conservative" diffusion, which can also be described as epidemic modeling. That model is fundamentally different from a random walk. This paper too is only concerned with discrete time processes.

Ghosh, Rumi, and Kristina Lerman. "Rethinking centrality: the role of dynamical processes in social network analysis." arXiv preprint arXiv:1209.4616 (2012).

This paper, a precursor to the Ghosh et al paper above, is also concerned with discrete time random walks. Furthermore, the path lengths that it analyzes are paths in a non-conservative process, that is, paths of infection in an epidemic.

Stojmirović, Aleksandar, and Yi-Kuo Yu. "Information flow in interaction networks." Journal of Computational Biology 14.8 (2007): 1115-1143.

This paper includes discussion of a diffusion process and average path lengths. However the diffusion process is specific to this paper (not a commonly-used process like the RL) and path lengths are observed empirically (ie, no mathematical expression for path length is derived).

Masuda, Naoki, Mason A. Porter, and Renaud Lambiotte. "Random walks and diffusion on networks." Physics reports 716 (2017): 1-58.

This paper, in contrast to those above, extensively covers both discrete and continuous time stochastic processes. However, the specific question we ask, namely, the mean path length in the RL model, is not addressed in this paper. This underscores that our problem is not just one of computing the probability of reaching a given node in a given amount of time (which this paper does address) but specifically of how many nodes the walker will pass through in a random amount of continuous time.

Minor comments
Aside from the above major concerns, below are my minor points and comments to help the authors improve their work:

3) In the Discussion: "We were surprised to see that the top-contributing sources were invariably direct neighbours of the top-ranking predictions in the STRING network. A partial explanation for this trend may be the fact that as many as 5,331 proteins in the STRING network were direct neighbors of at least one source protein, even when we considered only interactions with weight at least 0.9 (the STRING database deems edges with such weights to be of "very high quality"). Thus, the structure of the STRING network and central location of sources within it may cause the RL both to give high ranks only to direct neighbors of sources and to channel propagation primarily along these direct connections."
To recap, this part of the discussion is related to the fact that, even though global exploration by using teleports is favored over local neighbor-hopping by setting \alpha to a high value, the top contributions still come from directly connected seeds. To me, this is one of the most interesting findings in the paper that might also call into question the widespread use of diffusion-based prioritization on PPI networks if

it really is the case that, no matter how globally explored the network is, it is still the seeds directly neighboring the prioritized proteins that affect the prioritization the most. One question related to that is whether or not the same holds for proteins that were lowly ranked by RL, i.e. is RL (and potentially other random-walk based methods) capturing chiefly "local" contributions from seeds for proteins at the bottom of the list as well? I feel that this can be explored further by (1) trying PPI networks with different densities and degree distributions than STRING such as strictly experimental binary PPIs such as those derived from Y2H assays (e.g. Luck, Katja, et al. "A reference map of the human binary protein interactome." Nature 580.7803 (2020): 402-408.); (2) testing whether the number of seeds itself (in this case around 300 if I followed the methods correctly) is a contributing factor to this phenomenon where, as the authors noted in the discussion, the direct interactions of these seed proteins cover the majority of the network, "saturating" the random walk process in a sense. Would we see the same results if there were, say, only 30 seed proteins instead of 300? In general, I would request the authors to think a little bit to delve a little further into this, without disrupting the flow of the paper as it is now.

Thank you for these suggestions. We found the first idea to be appealing. We repeated our experiments with three PPI networks: BioGRID, BioGRID with Y2H assays only, and the high-quality HI-union network from the paper the reviewer suggested. We originally hypothesized that the effective diffusion would be higher for the top-ranking proteins of these networks, but surprisingly, the influence from direct neighbors was even greater for these networks, resulting in lower effective diffusion values (see Figures 3(f), S9, and Table 1). We discuss these results in the Analysis section.

We believe these points merit further investigation and plan to continue this research in a future paper.

4) Figure 1 seems too generic. Perhaps include some more details such as what the nodes are (red, virus, blue human proteins, etc.), node sizes, etc.

We have added a legend to the figure on the node colors and gradients.

5) Figure 2a - I could not find anywhere if the AUROC/AUPRC values are statistically significantly different between different methods. The median values are compared but it seems by looking at the error bars that the difference is perhaps not statistically significant with respect to the other methods.

We tested for statistical significance between the algorithms and added a discussion in the supplementary results section in “Comparison of Cross-Validation Results.”

6) Figure 2a caption: precision at 0.1 recall or 0.3 recall (latter one used in the text, former used in the caption)

We corrected the caption to “precision at 0.3 recall”.

7) Figure 2C - top-ranked, meaning top 332 or top 1000?

Figure 2C (now B) shows the top 332. We updated the text to clarify this difference.

8) The provenance tracing part of the analysis: an intuitive definition of \alpha would be helpful at the beginning of this section. Currently, it is introduced without such an explanation as to what it does, such as the damping or "teleportation" parameter equivalent in pagerank.

At the beginning of the section “Tracing the Provenance of Top-Ranking Proteins,” we attempt to give an intuitive idea of the effect of \alpha: “Hence for larger values of \alpha, the influence of the sources is diffused more broadly across the network.”

9) The flow of this results section should be revisited. The authors lead with a discussion on the sensitivity analysis of \alpha, which, in my opinion, is of secondary importance to the provenance tracing aspect. The most important part of the paper thus gets buried further down into the results section.

We agree with the reviewer’s suggestion. We have reorganized the results section so that provenance tracing comes earlier.

10) Figure 3A, figure order (comes after Figs 3B-E) in the text

We updated the figure order.

11) "The GO biological process "protein folding in endoplasmic reticulum" was also enriched in the top-ranking proteins (p-value 4.32 10-9 for RL and 0.28 for interactors of SARS-CoV-2)."
This sentence reads as if the ER related GO terms were identified through two independent processes, where in reality it was the GO term enrichment on the top-prioritized proteins that was done first and "protein folding on ER" was identified as a process of interest, and then provenance tracing was performed on this biological process because it was implicated by the enrichment analysis in the first place. Minor point but one pertains to the flow of the text nevertheless: I think it would be helpful to remind the reader the order of events that led to these results, i.e. first the identification of salient pathways of GO terms, and then a detailed x-ray of these pathways through provenance tracing.

We added the following sentence to the paragraph just before the section “The Role of Endoplasmic Reticulum Stress, HSPA5, and Anti-Clotting Drugs”:

“We remind the reader that we computed functions enriched in the top-ranking proteins, performed the provenance analysis independently, and then integrated the results in the protein networks we visualized.”

12) Typo: "we compute the precise contribution of each source's contribution to the score of u."

We have corrected it.

Close
