# Supplementary material for: Interpretable network propagation with application to expanding the repertoire of human proteins that interact with SARS-CoV-2
Source: Gigascience. 2021 Dec 29;10(12):giab082. doi: 10.1093/gigascience/giab082 (PMC8716363; doi:10.1093/gigascience/giab082)
Supplement: giab082_Response_to_Reviewer_Comments_Revision_1 [file giab082_response_to_reviewer_comments_revision_1.pdf]

Author's Response To Reviewer Comments

Close

Dear Scott,
We have made all the edits requested by you, including referring to our GigaDB dataset and registering our software with biotools and SciCrunch. I hope you find the paper acceptable for publication.
Murali

Close
