# Supplementary material for: Interpretable network propagation with application to expanding the repertoire of human proteins that interact with SARS-CoV-2
Source: Gigascience. 2021 Dec 29;10(12):giab082. doi: 10.1093/gigascience/giab082 (PMC8716363; doi:10.1093/gigascience/giab082)
Supplement: giab082_Reviewer_3_Report_Original_Submission — Arda Halu -- 6/14/2021 Reviewed [file giab082_reviewer_3_report_original_submission.pdf]

Reviewer Report

Title: Interpretable Network Propagation with Application to Expanding the Repertoire of Human Proteins that Interact with SARS-CoV-2

Version: Original Submission **Date:** 6/14/2021

Reviewer name: Arda Halu

Reviewer Comments to Author:

In their manuscript, Law and colleagues use network propagation to predict potential SARS-CoV-2-interacting human proteins. On the methods front, they propose a means to (1) track down the prioritization contributions of individual seed proteins experimentally documented to interact with the virus; (2) select the main parameter of their network propagation method of choice (regularized Laplacian - RL) in an unbiased manner by relating it to the expected shortest path length of the random walk.

Overall, the individual sections of the paper are clear and well-written. However, taken together, the two main analyses somewhat lack a unifying narrative and feel disconnected from each other. While the methodological contributions of the paper seem interesting, the core choice of methods in the application to SARS-CoV-2 seems inadequately justified. Below are my two major concerns pertaining to each part of the analysis.

1) In general, in the first (prioritization) part of the analysis, it is not clear to me what the actual goal is. RL is benchmarked against several types of network propagation-based and "traditional" prioritization approaches, then SVM is taken as a representative of the latter group. Proteins are then prioritized between these two methods and validated in silico by their overlap with literature and their functional annotations. As it stands, the point of discussing and comparing different methods is not clear. What makes RL stand out is not directly evident the way these results are presented. In fact, the entire first part of the results, in my view, does not make a sufficient case for the use of network propagation as the prioritization method of choice. It rather looks like other off-the-shelf methods perform comparably (Fig. 2a), and yield similar biological insights (Fig 2c). It seems to me that if comparison of methods was performed, the authors should at least focus on the unique insights provided by RL (e.g. in terms of GO terms), if any, compared to SVM.

2) Provenance tracing explored in the second part is very interesting as a premise, and the fact that it is relatively uninvestigated makes it an appealing topic. As the authors note, it is based on a simple principle: once the regularized Laplacian kernel is calculated, the values of the kernel matrix are row-sorted for each protein. While this is still, in a sense, a steady-state view, and the name "provenance tracing" inspires in the reader's mind a sequence of connections between seeds and targets, I think the authors did a good job showcasing how it simplifies the subnetworks related to each biological process of interest, improving interpretability. I am convinced by its utility in that sense. What I have been having trouble convincing myself about is the particular choice of RL, some claims around the novelty of the analytical results, and why \alpha matters in the first place. To elaborate:

2a) The rationale presented for putting emphasis on \alpha seems not so well-justified to me. The

authors state that they looked for a different way to determine alpha than looking at AUROC/AUPRC values since they varied little over a wide range of alpha. Does this not mean that the choice of alpha does not impact the prediction results in the case of RL? I guess I don't fully understand the point of the derivation other than a purely mathematical exercise (whose precedents seem to exist - see below). This also bears asking why RL was chosen in the first place. It sounds in the paper as if RL is the only choice of kernel that can be utilized this way to compute the contribution of the seed nodes, whereas many other types of kernels should work in a similar manner (see Fouss et al. cited by the authors as [18]). It seems that for provenance tracing, any type of kernel could work as the authors simply use the values corresponding to the seeds at the steady state kernel matrix to represent the contribution of each seed on the candidate protein's score. For example, RL is indeed similar to RWR, a widely used propagation method in biology (see KÃ¶hler, Sebastian, et al. "Walking the interactome for prioritization of candidate disease genes." The American Journal of Human Genetics 82.4 (2008): 949-958), in terms of the form of its kernel. deepNF, one of the methods considered in the benchmark what does comparably or better than RL, is also based on RWR. Could the authors comment on the above points?

2b) The authors take a mean-field approach relating teleportation/damping parameter (\alpha) to the expected value of path lengths given a network. The authors then use the actual network to determine the median path length between seeds and candidates and find the \alpha that corresponds to this value from their precomputed lookup table. In a way, we can see this parameter selection process as the tuning of the random walk according to the network at hand. Regarding the analytical results on average path lengths in random walks/diffusion processes, similar results seem to exist:

Yazdani, Majid, and Andrei Popescu-Belis. "A random walk framework to compute textual semantic similarity: a unified model for three benchmark tasks." 2010 IEEE Fourth International Conference on Semantic Computing. IEEE, 2010.

Ghosh, Rumi, et al. "Non-conservative diffusion and its application to social network analysis." arXiv preprint arXiv:1102.4639 (2011).

Ghosh, Rumi, and Kristina Lerman. "Rethinking centrality: the role of dynamical processes in social network analysis." arXiv preprint arXiv:1209.4616 (2012).

StojmiroviÄ‡, Aleksandar, and Yi-Kuo Yu. "Information flow in interaction networks." Journal of Computational Biology 14.8 (2007): 1115-1143.

Masuda, Naoki, Mason A. Porter, and Renaud Lambiotte. "Random walks and diffusion on networks." Physics reports 716 (2017): 1-58.

Could the authors elaborate on the difference of their approach and scope?

Aside from the above major concerns, below are my minor points and comments to help the authors improve their work:

3) In the Discussion: "We were surprised to see that the top-contributing sources were invariably direct neighbours of the top-ranking predictions in the STRING network. A partial explanation for this trend may be the fact that as many as 5,331 proteins in the STRING network were direct neighbors of at least one source protein, even when we considered only interactions with weight at least 0.9 (the STRING database deems edges with such weights to be of "very high quality"). Thus, the structure of the STRING network and central location of sources within it may cause the RL both to give high ranks only to direct neighbors of sources and to channel propagation primarily along these direct connections."

To recap, this part of the discussion is related to the fact that, even though global exploration by using

teleports is favored over local neighbor-hopping by setting \alpha to a high value, the top contributions still come from directly connected seeds. To me, this is one of the most interesting findings in the paper that might also call into question the widespread use of diffusion-based prioritization on PPI networks if it really is the case that, no matter how globally explored the network is, it is still the seeds directly neighboring the prioritized proteins that affect the prioritization the most. One question related to that is whether or not the same holds for proteins that were lowly ranked by RL, i.e. is RL (and potentially other random-walk based methods) capturing chiefly "local" contributions from seeds for proteins at the bottom of the list as well? I feel that this can be explored further by (1) trying PPI networks with different densities and degree distributions than STRING such as strictly experimental binary PPIs such as those derived from Y2H assays (e.g. Luck, Katja, et al. "A reference map of the human binary protein interactome." Nature 580.7803 (2020): 402-408.); (2) testing whether the number of seeds itself (in this case around 300 if I followed the methods correctly) is a contributing factor to this phenomenon where, as the authors noted in the discussion, the direct interactions of these seed proteins cover the majority of the network, "saturating" the random walk process in a sense. Would we see the same results if there were, say, only 30 seed proteins instead of 300? In general, I would request the authors to think a little bit to delve a little further into this, without disrupting the flow of the paper as it is now.

4) Figure 1 seems too generic. Perhaps include some more details such as what the nodes are (red, virus, blue human proteins, etc.), node sizes, etc.

5) Figure 2a - I could not find anywhere if the AUROC/AUPRC values are statistically significantly different between different methods. The median values are compared but it seems by looking at the error bars that the difference is perhaps not statistically significant with respect to the other methods.

6) Figure 2a caption: precision at 0.1 recall or 0.3 recall (latter one used in the text, former used in the caption)

7) Figure 2C - top-ranked, meaning top 332 or top 1000?

8) The provenance tracing part of the analysis: an intuitive definition of \alpha would be helpful at the beginning of this section. Currently, it is introduced without such an explanation as to what it does, such as the damping or "teleportation" parameter equivalent in pagerank.

9) The flow of this results section should be revisited. The authors lead with a discussion on the sensitivity analysis of \alpha, which, in my opinion, is of secondary importance to the provenance tracing aspect. The most important part of the paper thus gets buried further down into the results section.

10) Figure 3A, figure order (comes after Figs 3B-E) in the text

11) "The GO biological process "protein folding in endoplasmic reticulum" was also enriched in the top-ranking proteins (p-value 4.32 10-9 for RL and 0.28 for interactors of SARS-CoV-2)."

This sentence reads as if the ER related GO terms were identified through two independent processes, where in reality it was the GO term enrichment on the top-prioritized proteins that was done first and "protein folding on ER" was identified as a process of interest, and then provenance tracing was performed on this biological process because it was implicated by the enrichment analysis in the first place. Minor point but one pertains to the flow of the text nevertheless: I think it would be helpful to remind the reader the order of events that led to these results, i.e. first the identification of salient pathways of GO terms, and then a detailed x-ray of these pathways through provenance tracing.

12) Typo: "we compute the precise contribution of each source's contribution to the score of u."

Methods

Are the methods appropriate to the aims of the study, are they well described, and are necessary controls included? Choose an item.

Conclusions

Are the conclusions adequately supported by the data shown? Choose an item.

Reporting Standards

Does the manuscript adhere to the journal's guidelines on minimum standards of reporting? Choose an item.

Choose an item.

Statistics

Are you able to assess all statistics in the manuscript, including the appropriateness of statistical tests used? Choose an item.

Quality of Written English

Please indicate the quality of language in the manuscript: Choose an item.

Declaration of Competing Interests

Please complete a declaration of competing interests, considering the following questions:

- Have you in the past five years received reimbursements, fees, funding, or salary from an organisation that may in any way gain or lose financially from the publication of this manuscript, either now or in the future?
- Do you hold any stocks or shares in an organisation that may in any way gain or lose financially from the publication of this manuscript, either now or in the future?
- Do you hold or are you currently applying for any patents relating to the content of the manuscript?
- Have you received reimbursements, fees, funding, or salary from an organization that holds or has applied for patents relating to the content of the manuscript?
- Do you have any other financial competing interests?
- Do you have any non-financial competing interests in relation to this paper?

If you can answer no to all of the above, write 'I declare that I have no competing interests' below. If your reply is yes to any, please give details below.

I declare that I have no competing interests

I agree to the open peer review policy of the journal. I understand that my name will be included on my report to the authors and, if the manuscript is accepted for publication, my named report including any attachments I upload will be posted on the website along with the authors' responses. I agree for my report to be made available under an Open Access Creative Commons CC-BY license (http://creativecommons.org/licenses/by/4.0/). I understand that any comments which I do not wish to be included in my named report can be included as confidential comments to the editors, which will not be published.

Choose an item.

To further support our reviewers, we have joined with Publons, where you can gain additional credit to further highlight your hard work (see: https://publons.com/journal/530/gigascience). On publication of this paper, your review will be automatically added to Publons, you can then choose whether or not to claim your Publons credit. I understand this statement.

Yes Choose an item.
